# Synthesis of Furan and Benzene Derivatives Containing Unsaturated Substituents with Electron-Withdrawing Groups, and Analysis of Their Antimicrobial Activity

**DOI:** 10.3390/molecules31183350

**Published:** 2026-09-21

**Authors:** Mikhail V. Kalyaev, Irina A. Boyarskaya, Alexander Yu. Ivanov, Dar’ya V. Spiridonova, Kristina E. Borovkova, Sofia A. Brevnova, Artem O. Taraskin, Karina S. Gromozdova, Aleksander V. Vasilyev

**Affiliations:** 1 Department of Organic Chemistry, Institute of Chemistry, Saint Petersburg State University, Universitetskaya nab., 7/9, Saint Petersburg 199034, Russia; kalyaev2017@yandex.ru (M.V.K.); iralbo@yahoo.com (I.A.B.); 2 Department of General, Analytical and Applied Chemistry, Ufa State Petroleum Technical University, ul. Kosmonavtov 1, Ufa 450064, Russia; 3 Center for Magnetic Resonance, Research Park, Saint Petersburg State University, Universitetskiy pr., 26, Petrodvoretz, Saint Petersburg 198504, Russia; alexander.ivanov@spbu.ru; 4 Research Center for X-ray Diffraction Studies, Research Park, Saint Petersburg State University, Universitetskiy pr. 26, Petrodvoretz, Saint Petersburg 198504, Russia; daria.spiridonova@spbu.ru; 5 Research and Manufacturing Company «Home of Pharmacy», Zavodskaya st., 3-245, Kuzmolovskiy t.s., Leningrad Oblast, Saint Petersburg 188663, Russia; borovkova.ke@doclinika.ru (K.E.B.); brevnova.sa@doclinika.ru (S.A.B.); taraskin.ao@doclinika.ru (A.O.T.); gromozdova.ks@doclinika.ru (K.S.G.); 6 Department of Chemistry, Saint Petersburg State Forest Technical University, Institutsky per., 5, Saint Petersburg 194021, Russia

**Keywords:** furans, conjugated butadienes, carbocations, triflic acid, antimicrobial activity

## Abstract

Furan-2-yl substituted ethenes and conjugated butadienes containing two geminal electron-withdrawing groups, as well as phenylbutadienes with two electron acceptors, were synthesized from the corresponding aldehydes and various β-dicarbonyl compounds via aldol condensation. Reactions of these ethenylfurans and methyl 3-(benzofuran-2-yl)propenoate with arenes in triflic acid (TfOH) yield arylated 2,3-dihydrofurans and 2,3-dihydrobenzofurans, representing a novel synthetic approach to these dihydro derivatives. In contrast, analogous reactions of furanyl- and phenylbutadienes result in carbon–carbon bond cleavage through retro-aldol and related acid-promoted processes. We propose plausible multistep mechanisms and cationic intermediates for these transformations. The synthesized furans and related compounds, at a concentration of 64 μg/mL, exhibit moderate antimicrobial activity against the yeast-like fungus *Candida albicans*.

## 1. Introduction

Furans are important compounds in chemistry, biology, medicine, materials science, and many other fields of science and technology. Many practically useful compounds and materials, such as polymers [1,2,3,4], semiconductors for organic photovoltaics [5,6], and biologically active compounds and drugs [7,8,9,10,11,12,13,14], have been obtained from furan derivatives. Much attention has focused on furan synthesis [15,16,17,18] and the analysis of their chemical reactions [19,20,21,22,23]. A key aspect of furan chemistry is producing furfural and 5-hydroxymethylfurfural (5-HMF) from renewable lignocellulosic biomass and converting them into valuable compounds [24,25,26,27,28,29,30,31,32,33].

**Scheme 1:**
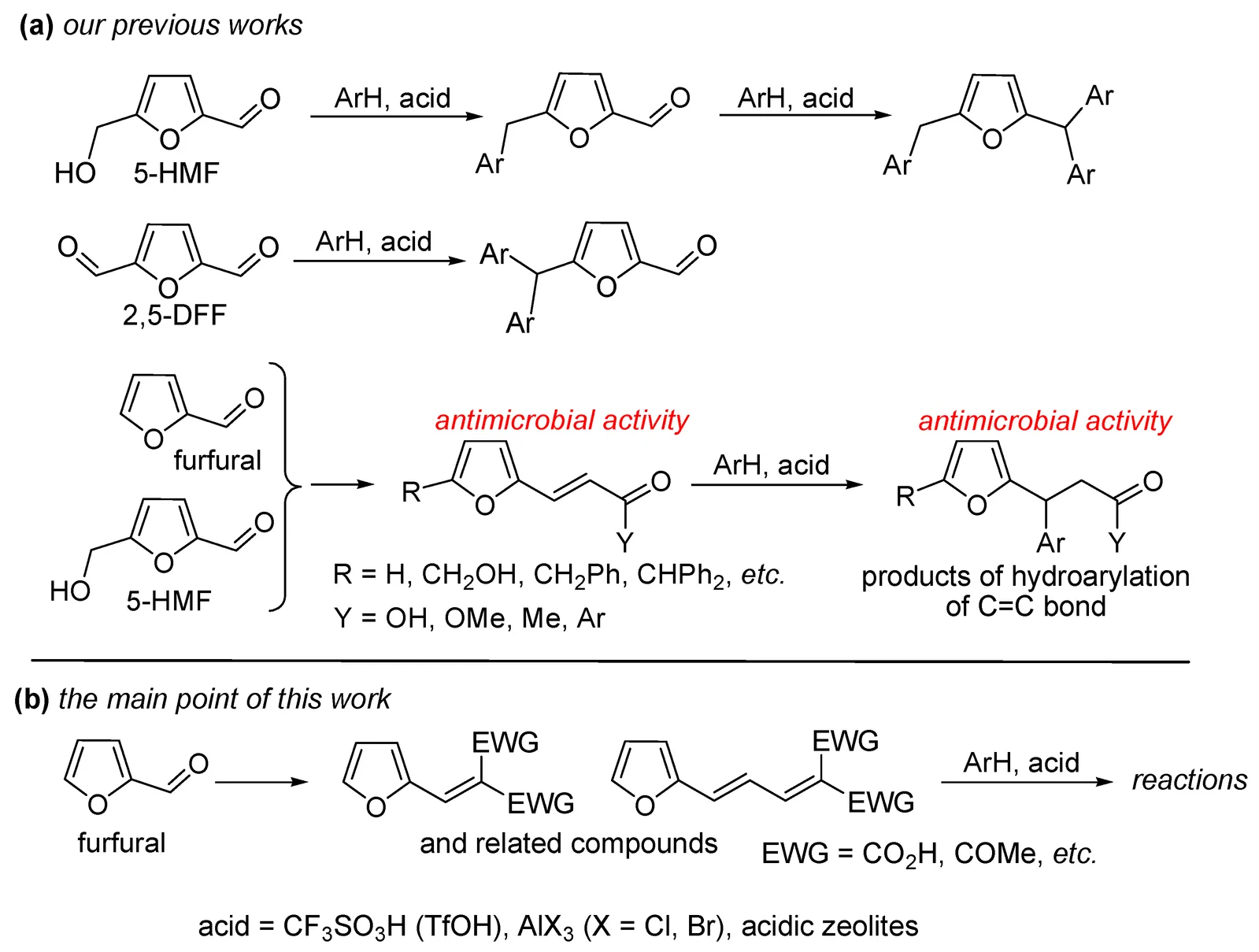
The results of our previous studies [34,35,36] and the main outcome of this work.

In our research, we have shown that electrophilic activation is an effective method for the synthesis of new furan derivatives. Thus, 5-hydroxymethylfurfural (5-HMF) and 2,5-diformylfurfural (2,5-DFF) underwent Friedel–Crafts reactions with arenes in the presence of strong Brønsted or Lewis acids, or acidic zeolites, to give the corresponding arylated hydroxymethyl and formyl group products (Scheme 1a) [34]. In the same reactions, furanyl-substituted propenoic acids and enones gave rise to products of hydroarylation of a carbon–carbon double bond (Scheme 1a) [35,36]. The obtained compounds showed antimicrobial activity [35,36,37].

Continuing our work on electrophilic activation of furan derivatives, we conducted the present study on the synthesis and biological activity of novel compounds (Scheme 1b). The main goals of this work included the synthesis of furans containing unsaturated substituents with two electron-withdrawing groups and related compounds; analysis of their reactions with arenes under the action of strong Brønsted acids [triflic acid TfOH (CF_3_SO_3_H) and sulfuric acid H_2_SO_4_], Lewis acid (AlCl_3_), and acidic zeolites; investigation of reaction cationic intermediates by means of NMR and DFT calculations; and analysis of the biological properties of the obtained compounds.

## 2. Results and Discussion

**Scheme 2:**
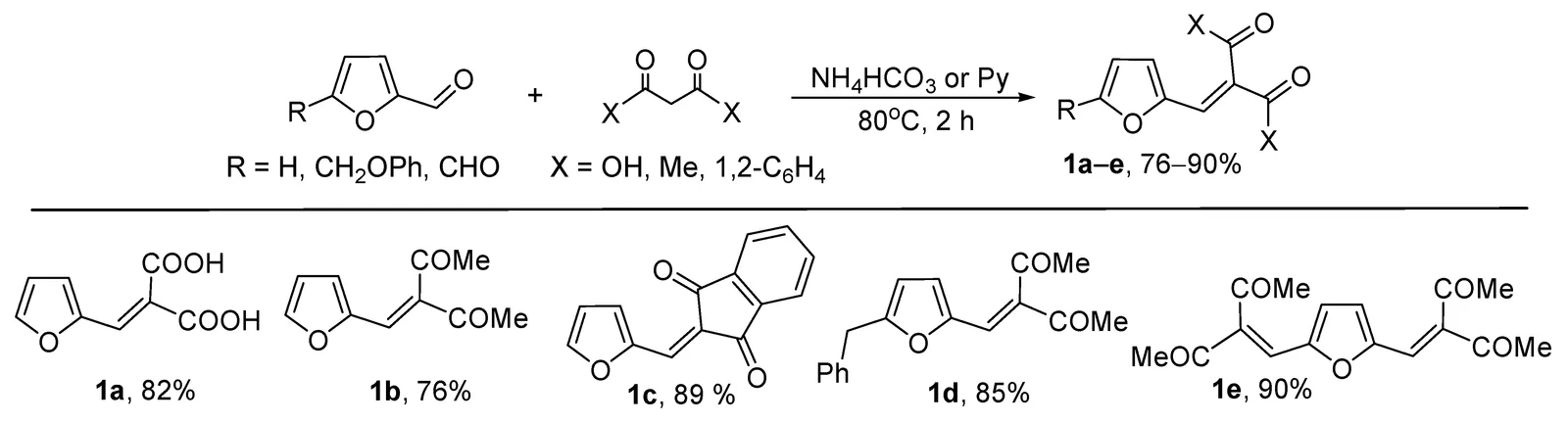
Synthesis of furans **1a**–**e** containing ethenyl substituents with two geminal electron acceptor groups.

**Scheme 3:**
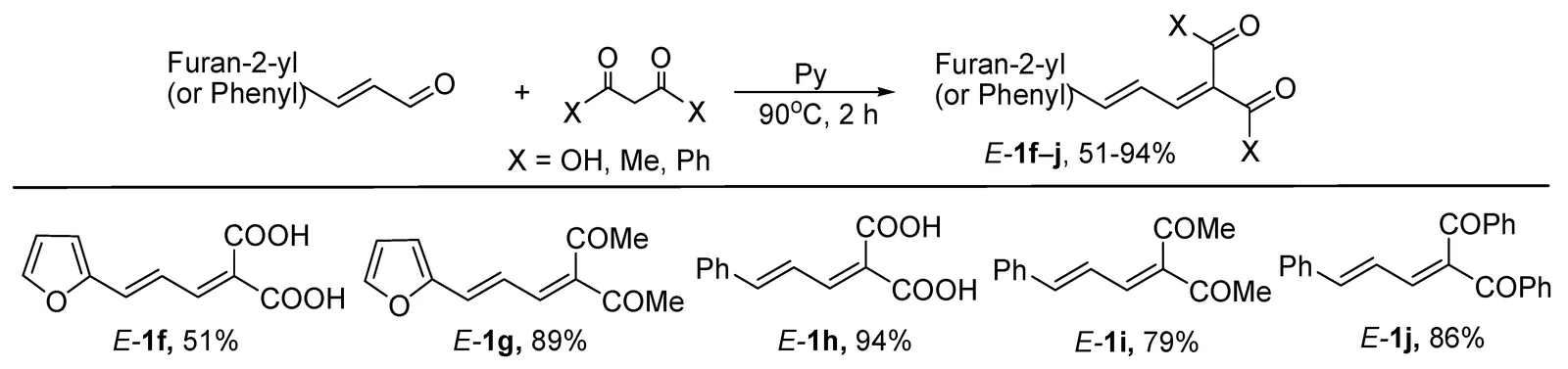
Synthesis of compounds *E*-**1f**–**j** containing butadienyl substituents with two geminal electron acceptor groups.

Furans **1a**–**e** containing ethenyl substituents with two geminal electron-withdrawing groups were synthesized by Knoevenagel condensation of furfural (for **1a**–**c**), 5-phenylmethylfufural (for **1d**), and 2,5-diformylfuran (for **1e**) with the corresponding β-dicarbonyl compounds (Scheme 2) using a known procedure (see Section 3). Compounds E-**1f–j** containing butadienyl substituents with two electron acceptors were obtained using the same method from 3-(furan-2-yl)propenal and cinnamaldehyde (Scheme 3).

**Scheme 4:**
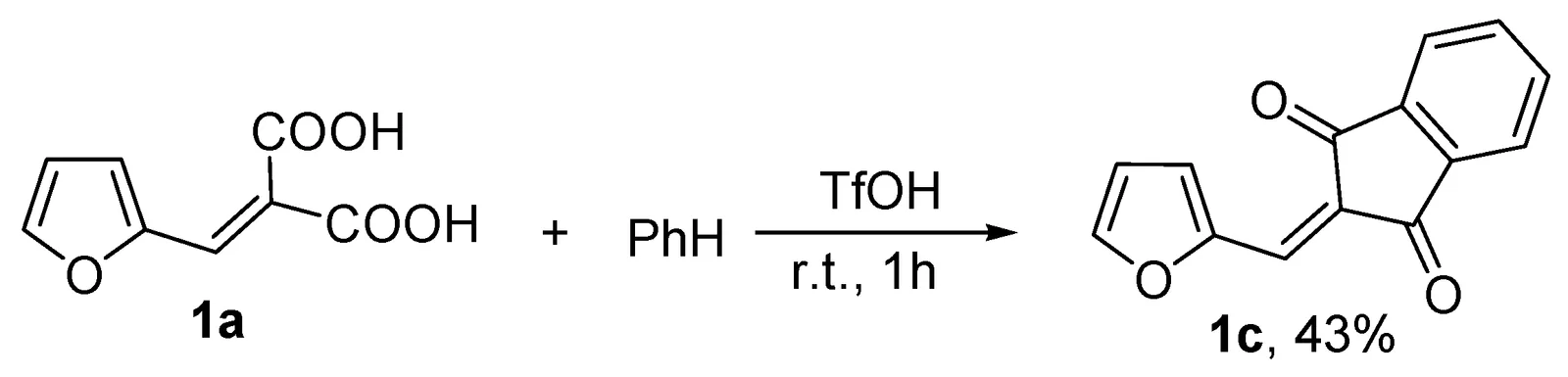
Reaction of furan **1a** with benzene in TfOH leading to acylation product **1c**.

We then investigated the reactions of compounds **1a**–**j** with arenes under electrophilic activation. The reaction of furan dicarboxylic acid **1a** with benzene in Brønsted superacid TfOH at room temperature for 1 h gave rise to Friedel–Crafts acylation product **1c** in a yield of 43% (Scheme 4). This method of formation of compound **1c** (Scheme 4) is less effective compared to the Knoevenagel condensation used in Scheme 2, an alternative way of synthesizing compound **1c**. However, reactions of compound **1a** with other electron-donating arenes (toluene, o-, m, p-xylenes, and anisole) under the same conditions led to complex mixtures of oligomeric materials.

The reaction of furan diketone **1b** with benzene afforded 2,3-dihydrofuran **2a** (Table 1). The highest yield of 70% for compound **2a** was achieved in TfOH at 80 °C for 0.25 h (entry 6). Under alternative reaction temperatures and times in this acid, yields of **2a** were lower (entries 4, 5, 7, and 8). Other acids, including H_2_SO_4_ (entries 1 and 2) and AlCl_3_ (entry 3), were found not to be suitable for this transformation.

**Scheme 5:**
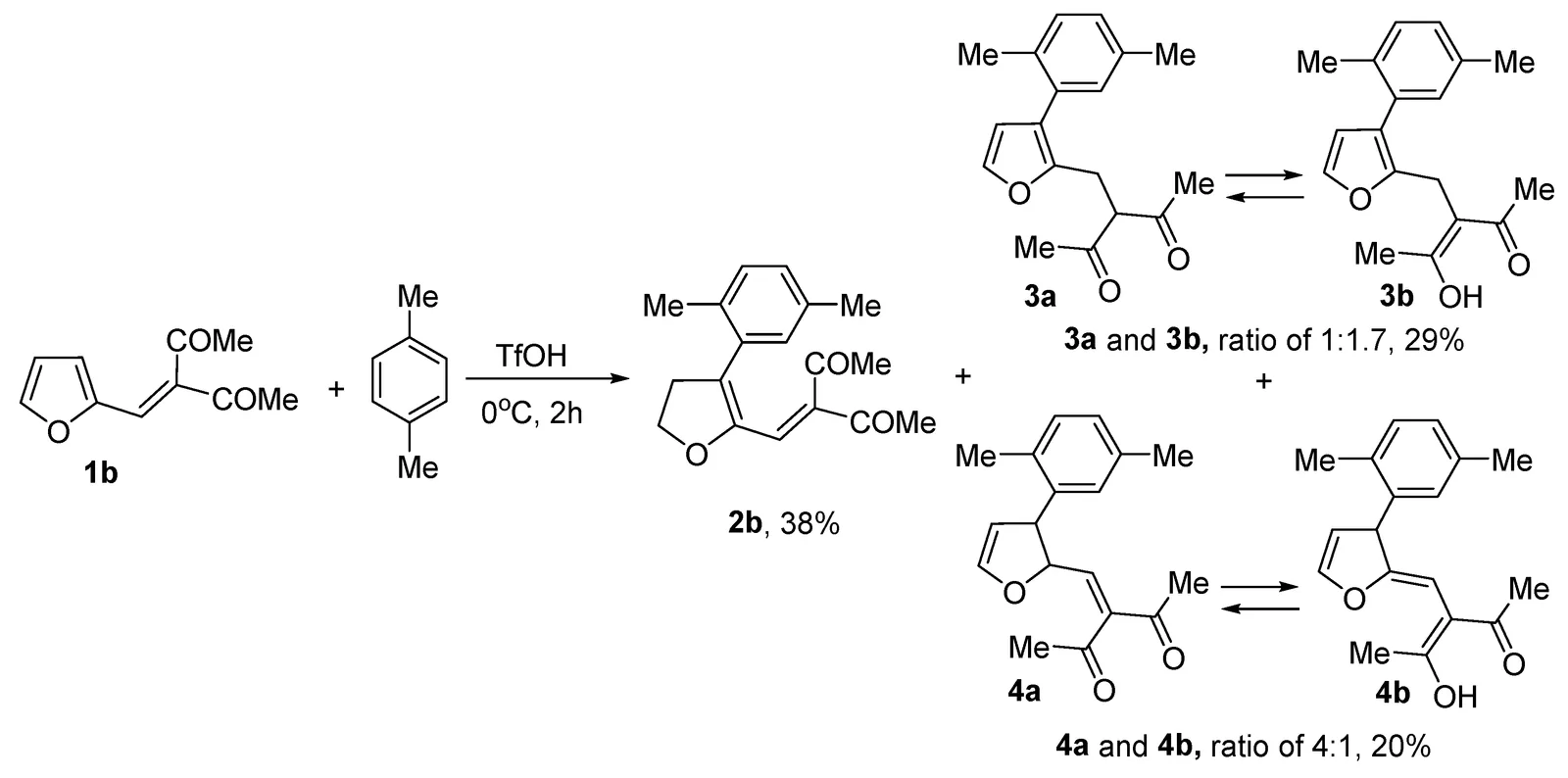
Reaction of furan **1b** with *p*-xylene in TfOH leading to compounds **2b**, **3a**,**b**, and **4a**,**b**.

The same reaction of furan **1b** with more donating *p*-xylene in TfOH led to 2,3-dihydrofuran **2b**, furans **3a**,**b**, and another type of 2,3-dihydrofurans **4a**,**b** (Scheme 5). Compounds **3a**,**b** and **4a**,**b** exist as equilibrium pairs of β-diketones and their enolic forms in CDCl_3_ solutions, according to NMR data.

**Scheme 6:**
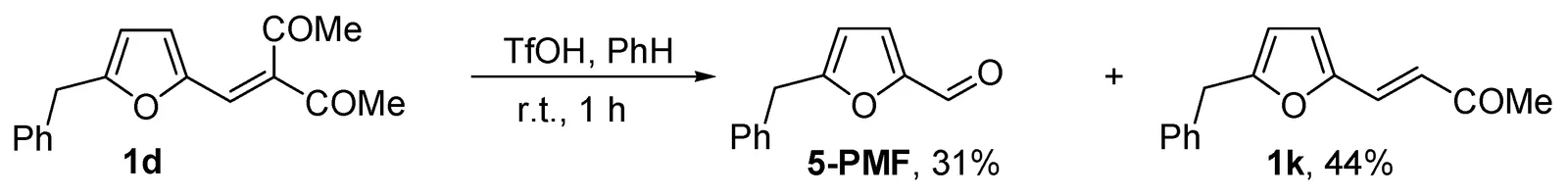
Transformation of furan **1d** in TfOH leading to 5-phenylmethylfufural (**5-PMF**) and enone **1k**.

Unfortunately, reactions of compound **1b** with other arenes (*o*-, *m*-xylenes, anisole, and veratrole), as well as reactions of furans **1c**–**e** with benzene and xylenes in the presence of TfOH or AlCl_3_ at 0 °C or room temperature, resulted in the formation of oligomeric compounds. One reason is retro-aldol degradation and related carbon–carbon bond cleavage in starting furans **1** in strong Brønsted and Lewis acids. Thus, compound **1d**, reacted with benzene in TfOH at room temperature for 1 h, gave products of carbon–carbon bond destruction: 5-phenylmethylfurfural (5-PMF) and enone **1k** (Scheme 6). The latter has been transformed into oligomers under these reaction conditions in the Brønsted superacid TfOH, as was shown by ourselves previously [36].

**Scheme 7:**
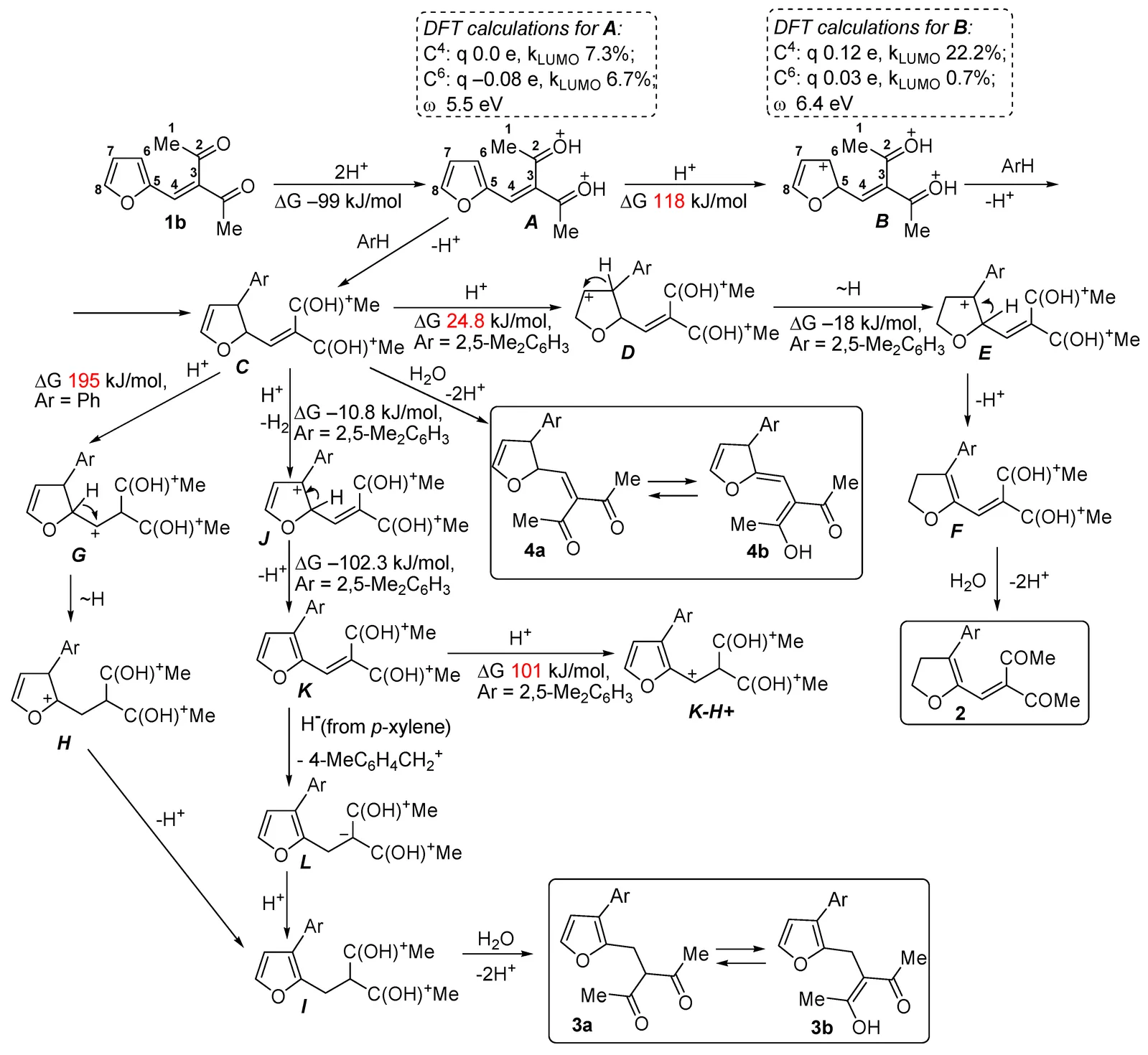
Plausible reaction mechanism of the transformation of furan **1b** into compounds **2**–**4** with data of DFT calculations of Gibbs energies ΔG of some reactions and selected electronic (charge on the carbons C^4^ and C^6^), orbital (contribution of atomic orbitals of the carbons C^4^ and C^6^ into LUMO, k_LUMO_) and electrophilic (global electrophilicity index ω) characteristics of species ***A*** and ***B***.

We have proposed a plausible reaction mechanism for the transformation of furan **1b** into compounds **2**–**4** using data from DFT calculations of Gibbs energies ΔG of some reactions, along with electronic and orbital characteristics (charge distribution, HOMO/LUMO energies, contribution of atomic orbitals to the LUMO, and global electrophilicity index ω [38]) of species ***A*** and ***B*** (see Scheme 7 and Supplementary Materials for the detailed DFT calculations). Without transition-state calculations, the proposed reaction pathway is supported by thermodynamic analysis, but the calculations do not establish the corresponding activation barriers or kinetic preference.

Initially, both carbonyl oxygen atoms are protonated in TfOH, leading to dication ***A***. The formation of the latter is thermodynamically favorable; the Gibbs energy of protonation **1b**→***A*** is negative ΔG −99 kJ/mol (see Scheme 7). A consequent protonation of the C=C bond in the furan ring of species ***A***, giving trication ***B***, is extremely unfavorable, with a value of ΔG 118 kJ/mol, and the formation of species ***B*** is very unlikely. Most likely, dication ***A*** is electrophilic enough, with a rather high electrophilicity index ω of 5.5 eV [39], to react with arene, affording species ***C***. According to DFT calculations, dication ***A*** has almost no charge at carbon C^6^, which means that the electrophilicity of this carbon is mainly characterized by the orbital factor (k_LUMO_ 6.7%), rather than charge one. The electrophilic center in dication ***A*** is carbon C^6^, rather than C^4^, although the latter has similar electronic (charge −0.08 e) and orbital (k_LUMO_ 6.7%) properties. See also Scheme 7 for the comparison of the calculated properties of trication ***B***.

Thus, species ***C*** is the secondary key reactive intermediate and can undergo different transformations depending on the reaction conditions and the electronic donor–acceptor properties of the aryl substituent in its structure. Hydrolysis of this species furnishes keto-enol tautomers **4a** and **4b**. Another reaction pathway is its protonation at the α-position of the 2,3-dihydrofuran moiety with the formation of species ***D***, which undergoes a hydride shift to the more stable benzyl type cation ***E***; deprotonation of the latter results in cation ***F***, and final hydrolysis affords compound **2**. Calculations of Gibbs energies for the sequence ***C***→***D***→***E*** show that the first reaction ***C***→***D*** with ΔG 24.8 kJ/mol is not favorable but possible. However, the next step ***D***→***E*** has a negative value of ΔG −18 kJ/mol, and further deprotonation stages ***E***→***F***→**2** are usually thermodynamically favorable.

One more pathway for cation ***C*** is the formation of keto-enol tautomers **3a** and **3b**, which contain a formally reduced side-chain C=C bond. Protonation of this bond in species ***C***, to give trication ***G***, is unlikely because of the very high Gibbs energy of this process (ΔG 195 kJ; see Scheme 7). Therefore, the reaction pathway through cations ***G***, ***H***, and ***I*** should be excluded. An alternative route may involve generating benzyl type cation ***J*** by hydride-ion extraction from cation ***C***, which may occur in superacids [40], followed by deprotonation of species ***J*** to give cation ***K***. Calculated Gibbs energies for these reactions ***C***→***J***→***K*** are negative (Scheme 7), indicating thermodynamic favorability. However, analogously, in the transformations ***A***→***B*** and ***C***→***G***, protonation of species ***K*** with the formation of species ***K-H^+^*** is very unfavorable, with a value of ΔG 101 kJ/mol. This suggests another pathway for transforming ***K*** into target compounds **3a** and **3b**. Species ***K*** may be electrophilic enough to abstract a hydride ion from *p*-xylene molecules, leading to species ***L***; this type of ionic hydrogenation is also characteristic of superacid-promoted processes [39]. Protonation of species ***L*** affords cation ***I***, and hydrolysis of the latter furnishes keto-enol tautomers **3a** and **3b**.

2,3-Dihydrofurans are especially important compounds because of their biological activity. They occur in nature and are used in organic synthesis; see selected research papers [41,42,43,44,45] and reviews [46,47] on their synthesis and properties.

**Scheme 8:**
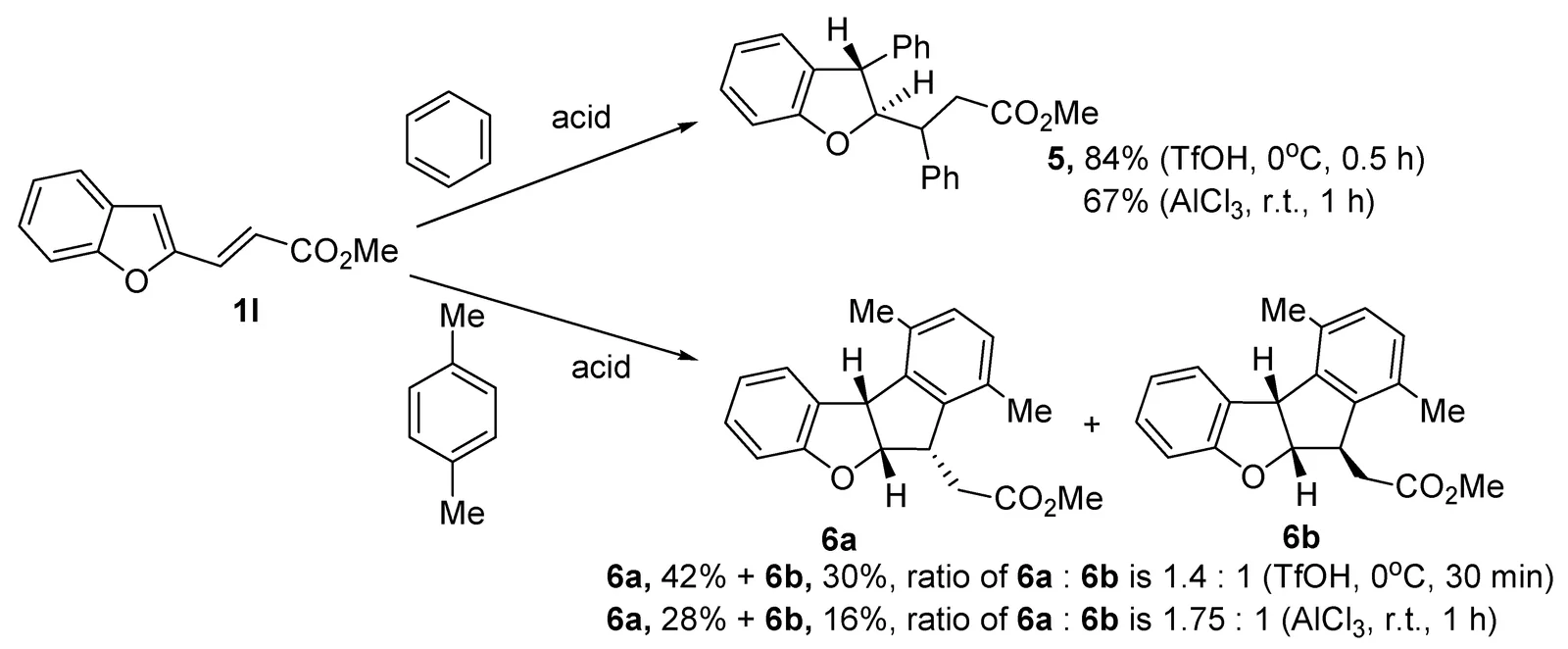
Reaction of benzofuran **1l** with benzene and *p*-xylene under the action of TfOH or AlCl_3_ leading to compounds **5** and **6a**,**b** (absolute configuration for one of the enantiomers is shown).

We also carried out similar reactions of benzofuran **1l**, containing an ethenyl substituent conjugated with an acceptor ester group, with benzene and *p*-xylene in the presence of TfOH or AlCl_3_ (Scheme 8). Reaction with benzene afforded 2,3-dihydrobenzofuran **5** as the product of *bis-*hydrophenylation of the C=C bonds both in the side chain and furan ring. Reaction with *p*-xylene led to the pair of diastereomeric indano-dihydrobenzofurans **6a** and **6b**.

**Scheme 9:**
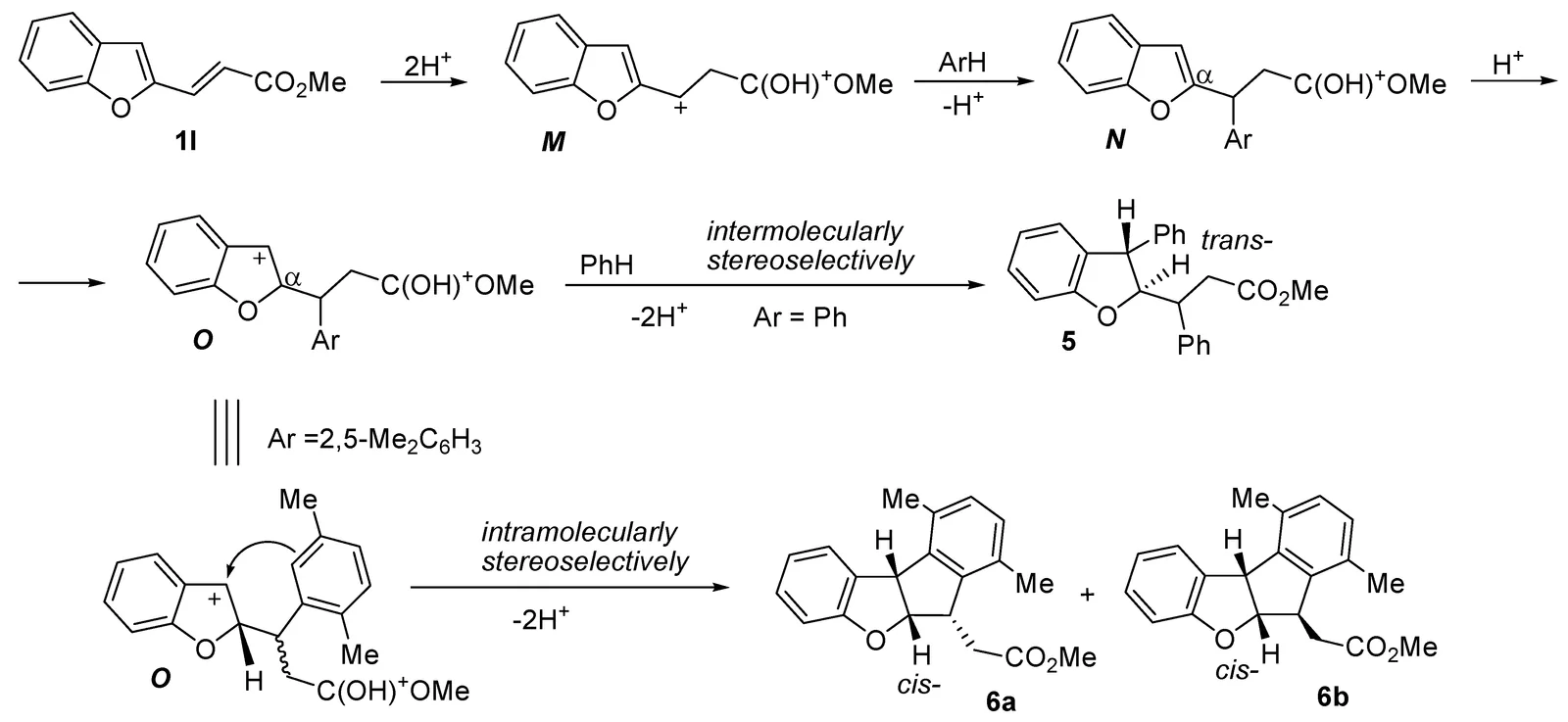
Plausible reaction mechanism of the transformation of benzofuran **1l** into compounds **5** and **6a,b** (absolute configuration for one of the enantiomers is shown).

A plausible reaction mechanism for the formation of compounds **5** and **6a**,**b** from benzofuran **1l** is presented in Scheme 9. Protonation of the carbonyl oxygen and the side-chain C=C bond of compound **1l** gives rise to dication ***M***. The latter interacts with arene, forming species ***N***, which is protonated at the α-carbon of the benzofuran ring, leading to cation ***O***. This species reacts with one more benzene molecule, yielding compound ***5***. With the more nucleophilic *p*-xylene, cyclization occurs, and isomers **6a** and **6b** are formed.

The stereochemical structure of compounds **5** and **6a**,**b** was determined by NOESY correlations (see spectra in Supplementary Materials). The stereoselective formation of 2,3-dihydrobenzofurans **5** and **6a**,**b** is likely explained mainly by steric factors. Thus, during the formation of compound **5**, the benzene molecule attacks intermolecularly cation ***O*** from its less sterically hindered plane, namely, from the side of hydrogen at the α-carbon, rather than from the bulky substituent, which gives trans-orientation of bulky substituents in positions 2 and 3 in dihydrobenzofuran **5** (Scheme 9). In contrast, cyclization in species ***O*** containing a *p*-xylyl substituent proceeds via formation of the less-strained structures **6a**,**b**, with *cis*-orientation of the hydrogen atoms at the joint C–C bond between the indane and dihydrobenzofuran subunits. Moderate diastereoselectivity in the formation of compound **6a** may also be explained by spatial factors (see the **6a**:**6b** ratios in Scheme 8).

Analogous to 2,3-dihydrofurans, 2,3-dihydrobenzofurans also occur widely in nature; nowadays, great attention is paid to the synthesis and investigation of the biological properties of these compounds (see recent reviews on this issue [48,49,50,51,52,53]).

We then studied reactions of electron-deficient butadienyl substituted furans *E*-**1f**,**g** and benzenes *E*-**1h**–**j**. Furans *E*-**1f**,**g** reacted with arenes in the presence of various acids, such as TfOH, AlCl_3_, and zeolites, to give complex mixtures of oligomeric materials. This may be due to retro-aldol and other carbon–carbon bond-cleavage reactions in the starting furans under strong acidic conditions; the resulting degradation products led to oligomers (see a similar reaction in Scheme 6).

Benzene derivatives *E*-**1h**–**j** gave better results. Transformations of compounds *E*-**1h**–**j** in TfOH were complex. Reactions with benzene resulted in mixtures of products **7**–**10** depending on temperature and time (Table 2). Compounds **7**–**9** were obtained at room temperature for 12–24 h in high general yields of 82–96% (entries 1, 2, and 5). At higher temperatures, all reaction products degraded to diphenylethanal **10**, which exists in enolic form (entries 3, 6, and 8), or to oligomers (entry 4).

The same reactions of compounds *E*-**1h**–**j** with benzene under the action of H_2_SO_4_ or AlCl_3_ did not proceed at room temperature but gave oligomeric compounds at 80 °C.

Zeolite-promoted reactions with benzene afforded two products, **11** and **12**, in a roughly 1:1 ratio and moderate overall yields of 47–71% (Table 3).

The data obtained (Table 2 and Table 3) reveal that compounds *E*-**1h**–**j** in TfOH undergo cleavage of various carbon–carbon bonds in their structures. One such retro-aldol reaction product is cinnamaldehyde, which further reacts with benzene in TfOH, leading to compounds **7**–**10**. Exactly the same compound **7** was obtained in AlCl_3_-promoted Friedel–Crafts reactions of cinnamaldehyde with benzene in the work [54]. In addition, the work [54] postulated the generation of precursors to compounds **8**–**12**. The formation of compounds **7**–**12** in our study proceeds in accordance with the multistep cationic mechanism described in [54].

**Scheme 10:**
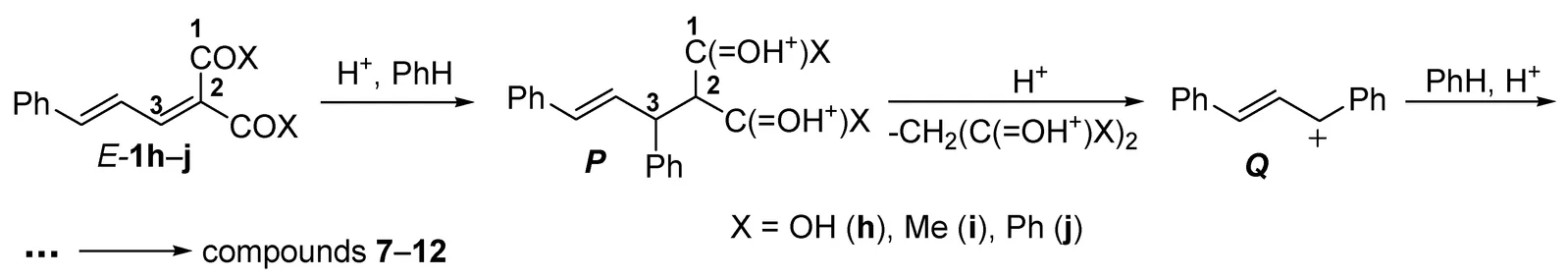
Plausible reaction mechanism of the transformation of phenylbutadienes *E*-**1h**–**j** into compounds **7**–**12**.

The first steps of the plausible reaction mechanism for the transformation of phenylbutadienes *E*-**1h**–**j** into compounds **7**–**12** are given in Scheme 10. Reaction of compounds *E*-**1h**–**j** with benzene furnishes cation ***P*** as a product of hydrophenylation of the C^2^=C^3^ bond. The next step is a cleavage of the single carbon–carbon bond C^2^–C^3^ by its protonation, with elimination of the diprotonated form of the β-dicarbonyl compound CH_2_(C(=OH^+^)X)_2_ and the formation of cation ***Q***. The latter is the key reaction intermediate in the electrophilic transformation of cinnamaldehyde, as it has been described in the work [54], and the next reaction stages leading to compounds **7**–**12** are very similar to the described reaction mechanism in the paper [54].

To estimate the reactivity of compound *E*-**1h**, we performed an NMR study of its protonated form in deuterated sulfuric acid (D_2_SO_4_). It was found that a stable *O,O*-dideutarated (diprotonated) form **Ah–2D** was formed in D_2_SO_4_ (Table 4; see NMR spectra in Supplementary Materials). As was mentioned above, compound *E*-**1h** did not react with benzene in H_2_SO_4_ at room temperature. This indicates that cation **Ah–2D** (or the corresponding diprotonated form *E*-**1h** in H_2_SO_4_) is a weak electrophile and must be further protonated onto the C=C bond of the butadiene system to react with nucleophiles (benzene molecules or acid counteranions). H_2_SO_4_ is not acidic enough for that. This protonation occurs in stronger acids, such as the Brønsted superacid TfOH or acidic zeolites (Table 2 and Table 3).

Comparison of ^13^C NMR spectra for compound *E*-**1h** and its cation ***Ah–2D*** shows large downfield shifts of signals for carbons C^1^(C^1^′) (Δδ 10.2–11.4 ppm), C^3^ (Δδ 26.6 ppm) and C^5^ (Δδ 28.6 ppm) in the cation (Table 4). In ^1^H NMR, the same downfield shifts were observed for the aromatic and butadiene protons H^3^ (Δδ 1.62 ppm), H^4^ (Δδ 0.86 ppm), and H^5^ (Δδ 0.71 ppm). The NMR data support protonation and are consistent with substantial positive-charge delocalization in species ***Ah–2D***.

At the final stage of this study, the antimicrobial activity of the starting compounds **1** and products of their transformations **2**–**12** was investigated against the bacteria *Escherichia coli* ATCC 25922 and *Staphylococcus aureus* ATCC 6538-P and the yeast-like fungi *Candida albicans* (ATCC 10231) (Table 5; see details in Supplementary Materials). Individual substances **1**–**2**, **5**, and **7**–**10** and mixtures **3a** + **3b**, **6a** + **6b**, and **11** + **12** were studied in biological tests. All tested compounds suppressed the growth of the yeast-like fungus *Candida albicans* at concentrations from 32 μg/mL to 128 μg/mL; for most, the minimum inhibitory concentration (MIC) was 64 μg/mL. For the *Staphylococcus aureus* and *Escherichia coli* strains, MICs for most compounds were 128 μg/mL. Compounds **5**–**6** and **8**–**12** did not demonstrate antimicrobial activity against *Staphylococcus aureus* and *Escherichia coli* strains.

Summarizing our data on the antimicrobial activity of furan derivatives with a specific structure (see our works [35,36] and Scheme 1a), one may conclude that the main biological property of these furans is antifungal activity against the yeast-like fungus *Candida albicans*.

## 3. Experimental Part

General information. The NMR spectra of solutions of compounds were recorded on Bruker-400 or Bruker-500 spectrometer at 25 °C at 400 and 500 MHz for ^1^H and 100 and 125 MHz for ^13^C NMR spectra, respectively. The residual proton-solvent peaks CDCl_3_ (δ 7.26 ppm) and DMSO-d_6_ (δ 2.50 ppm) for ^1^H NMR spectra and the carbon signals of CDCl_3_ (δ 77.0 ppm) and DMSO-d_6_ (δ 39.52 ppm) for ^13^C NMR spectra were used as references. NMR spectra in the deuterosulfuric acid D_2_SO_4_ were referenced to the signal of CH_2_Cl_2_ added as internal standard: δ 5.30 ppm for ^1^H NMR spectra and δ 53.52 ppm for ^13^C NMR spectra. HRMS was carried out on instruments Bruker maXis HRMS-ESI-QTOF and Varian 902-MS MALDI Mass Spectrometer. GC-MS spectra were taken on Shimadzu GC-MS QP-2010 SE machine. The preparative reactions were monitored by thin-layer chromatography carried out on silica gel plates (Alugram SIL G/UV-254), using UV light for detection. Column chromatography was performed on Merk silica gel 60 using mixtures of ethyl acetate and hexane as an eluent.

Zeolites CBV-500 and CBV-720 were purchased from the company Zeolyst International.

**X-Ray study.** A suitable crystal of compound **11** was studied using Rigaku «XtaLAB Synergy-S» diffractometer (monochromated Cu Kα radiation, λ= 1.54184 Å) with CrysAlisPro software. The temperature was kept at 100 K throughout the experiment. Empirical absorption correction was applied in CrysAlisPro (Agilent Technologies, 2014, Santa Clara, CA, USA) program complex using spherical harmonics, implemented in SCALE3 ABSPACK scaling algorithm. The structures were solved by SHELXT program [55], using least squares minimization in anisotropic (for non-hydrogen atoms) approximation and refined with the SHELXL package [56] incorporated in the Olex2 program package [57]. The hydrogen atoms were introduced to the geometrically calculated positions and refined by attaching themselves to the corresponding parent atoms. Supporting crystallographic data for this paper have been deposited at the Cambridge Crystallographic Data Centre (CCDC number 2577879 for **11**) and can be obtained free of charge via www.ccdc.cam.ac.uk/data_request/cif (accessed on 31 July 2026).

**DFT calculations.** All computations were carried out at the DFT/HF hybrid level of theory using hybrid exchange functional B3LYP by using GAUSSIAN 09 program packages [58]. The geometry optimizations were performed using the 6-311+G(2d,2p) basis set (standard 6-311G basis set added with polarization (d,p) and diffuse functions). Optimizations were performed on all degrees of freedom and solvent-phase optimized structures were verified as true minima with no imaginary frequencies. The Hessian matrix was calculated analytically for the optimized structures in order to prove the location of correct minima and to estimate the thermodynamic parameters. Solvent-phase calculations used the Polarizable Continuum Model (PCM, solvent = water).

**Study of biological activity.** Minimum inhibitory concentrations of compounds against *Escherichia coli* ATCC 25922, *Staphylococcus aureus* ATCC 6538-P, and *Candida albicans* ATCC 10231 were determined using broth microdilution as described in ISO 20776-1:2019 [59] and ISO 16256:2021 [60] (see details in Supplementary Materials).

**Procedures for the synthesis of compounds.**

General procedure for the synthesis of compounds **1a**–**f** by the Knoevenagel condensation of aldehydes with β-dicarbonyl compounds [61,62]. A mixture of aldehyde (10 mmol) and β-dicarbonyl compound (10 mmol) in pyridine (10 mL) was stirred at 90 °C for 2 h for the synthesis of compounds **1b**–**e**,**g**–**j**, or a mixture of the same amounts of aldehyde and β-dicarbonyl compound in THF (10 mL) with NH_4_HCO_3_ (79 mg, 1 mmol) was stirred at 50 °C for 2 h for the synthesis of compounds **1a**,**f**. The reaction mixture was then poured into water (50 mL), and concentrated aqueous hydrochloric acid (HCl) was added dropwise to pH 4–5. The mixture was extracted with chloroform (3 × 50 mL). The combined extracts were washed with water (3 × 50 mL) and dried over Na_2_SO_4_. The solvent was evaporated at reduced pressure to obtain the target compound.

General procedure for the reaction of compounds **1** with arenes in Brønsted acids, TfOH or H_2_SO_4_. Synthesis of compounds **2**–**6** and **7**–**10**. Compound **1** (0.37 mmol) was added to a mixture of arene (1.1 mmol) and acid [TfOH (0.5 mL, 16 eq.) or H_2_SO_4_ (0.94 mL, 16 eq.)]. The reaction mixture was stirred at various temperatures and times, as is indicated in Table 1 and Table 3 and Scheme 4, Scheme 5, Scheme 6 and Scheme 8. The reaction mixture was then poured into water (50 mL) and extracted with chloroform (3 × 50 mL). The combined extracts were washed with water (3 × 50 mL) and dried over Na_2_SO_4_. The solvent was removed at reduced pressure. The resulting residue was subjected to column chromatography on silica gel using a mixture of ethyl acetate and hexane as an eluent.

**General procedure for the reaction of compounds 1 with arenes under the action of AlCl_3_.** S**ynthesis of compounds 5 and 6.** Compound **1** (0.37 mmol) was added to a suspension of AlCl_3_ (267 mg, 2 mmol, 5.5 eq.) in arene (2 mL) at room temperature. The reaction mixture was stirred at room temperature for 1 h, then poured into water (50 mL) and extracted with chloroform (3 × 50 mL). The combined extracts were washed with water (3 × 50 mL) and dried with Na_2_SO_4_. The solvent was distilled at reduced pressure. The resulting residue was subjected to column chromatography using a mixture of ethyl acetate and hexane as an eluent.

General procedure for the reaction of compounds 1 with arenes under the action of acidic zeolites (CBV-500 or CBV-720). Synthesis of compounds 11 and 12. A mixture of compound 1 (0.37 mmol), benzene (5 mL) and 200 mg of zeolites (CBV-500 or CBV-720), which was preliminary pre-calcined for 3 h at 450 °C, was heated in a high-pressure glass tube at 150° C for 100 h with intensive stirring. Then the reaction mixture was cooled down to room temperature. Zeolite was filtered off on a glass filter. To extract the reaction products from the zeolite, it was washed several times with MeOH (4 × 20 mL). Then the organic phases were combined, and the solvents were removed under reduced pressure. The resulting residue was subjected to column chromatography using a mixture of ethyl acetate and hexane as an eluent.

**Characterization of compounds.** Synthesis and properties of *E*-4-(5-(phenylmethyl)furan-2-yl)but-3-en-2one **1k** [36] and methyl *E*-3-(benzofuran-2-yl)propenoate **1l** [35] were described by ourselves previously.

**2-[(Furan-2-yl)methylidene]malonic acid (1a)** [61] was obtained from furfural and malonic acid in yield of 82%. Light orange solid. M.p. 80–82 °C. ^1^H NMR (400 MHz, (CD_3_)_2_SO): δ = 6.65 br.s (1H_hetarom_.), 6.98 br.s (1H_hetarom._), 7.31 br.s (1H, CH), 7.85 br.s (1H_hetarom._). ^13^C NMR (100 MHz, (CD_3_)_2_SO): δ = 112.8, 117.8, 123.7, 125.1, 146.7, 148.6, 165.2, 167.2.

**3-[(Furan-2-yl)methylidene]pentane-2,4-dione(1b)** [61] was obtained from furfural and pentan2,4-dione in yield of 76%. Light orange oil. ^1^H NMR (400 MHz, CDCl_3_): δ = 2.36 s (3H, Me), 2.43 s (3H, Me), 6.51 dd (1H_hetarom._, *J* = 3.4, 1.8 Hz), 6.78 d (1H_hetarom._, *J* = 3.4 Hz), 7.16 s (1H, CH), 7.55 d (1H_hetarom._, *J* = 1.8 Hz). ^13^C NMR (100 MHz, CDCl_3_): δ = 26.2, 31.6, 113.1, 118.4, 125.1, 138.5, 146.7, 149.0, 196.0, 204.5.

**2-(Furan-2-ylmethylene)indane-1,3-dione (1c)** [62] was obtained from furfural and indan1,3-dione in yield of 89%. It was also obtained from compound **1a** and benzene in TfOH at room temperature for 1 h in yield of 43%. Light orange solid. M.p. 208–210 °C. ^1^H NMR (400 MHz, CDCl_3_): δ = 6.72–6.73 m (1H), 7.76–7.81 m (4H), 7.96–7.98 m (2H), 8.58–8.59 m (1H). ^13^C NMR (100 MHz, CDCl_3_): δ = 114.8, 123.1, 123.3, 124.9, 129.4, 135.0, 135.3, 140.6, 142.5, 149.2, 151.5, 189.2, 190.2.

**3-{[(5-Phenylmethyl)furan-2-yl]methylidene}pentane-2,4-dione (1d)** was obtained from 5-phenylmethylfurfural and pentane-2,4-dione in yield of 85%. Light orange oil. ^1^H NMR (400 MHz, CDCl_3_): δ = 2.25 s (3H, Me), 2.31 s (3H, Me), 3.95 s (2H, CH_2_), 6.15 d (1H_hetarom._, *J* = 3.4 Hz), 6.68 d (1H_hetarom._, *J* = 3.3 Hz) 7.07 s (1H, CH), 7.19–7.21 m (2H_arom._), 7.23–7.26 m (1H_arom._), 7.28–7.32 m (2H_arom._). ^13^C NMR (100 MHz, CDCl_3_): δ = 26.1, 31.2, 34.9, 110.1, 119.9, 125.1, 127.0, 128.8, 128.9, 136.7, 137.3, 148.0, 160.1, 195.8, 204.6. HRMS, *m*/*z* calculated for C_17_H_16_O_3_ [M+H]: 269.1172. Found: 269.1172.

**2,5-Bis [2,2-di(methylcarbonyl)ethen-1-yl]furan (1e)** was obtained from 2,5-diformylfuran and pentane-2,4-dione in yield of 90%. Light orange solid. M.p. 103–105 °C. ^1^H NMR (400 MHz, CDCl_3_): δ = 2.37 s (6H, Me), 2.44 s (6H, Me), 6.81 s (2H_hetarom._), 7.06 s (2H, CH). ^13^C NMR (100 MHz, CDCl_3_): δ = 26.4, 31.3, 120.1, 124.0, 140.6, 152.1, 195.7, 203.8. HRMS, *m*/*z* calculated for C_16_H_16_O_5_ [M+Na]: 311.1250. Found: 311.1254.

**2-[(3-Furan-2-yl)prop-2-en-1-ylidene]malonic acid (1f)** [63] was obtained from 3-(furan-2-yl)propenal and malonic acid in yield of 51%. Light orange solid. M.p. 190–192 °C. ^1^HNMR (400 MHz, (CD_3_)_2_SO): δ = 6.60 dd (1H_hetarom.,_ *J* = 3.2, 1.9 Hz), 6.76 d (1H_hetarom.,_ *J* = 3.2 Hz), 7.00 dd (1H, CH, *J* = 15.3, 11.6 Hz), 7.11 d (1H, CH=, *J* = 15.3 Hz), 7.41 d (1H, CH=, *J* = 11.6 Hz), 7.78 br.s (1H_hetarom._). ^13^C NMR (100 MHz, (CD_3_)_2_SO): δ = 112.8, 114.4, 121.0, 125.9, 130.1, 142.6, 145.4, 151.4, 166.0, 166.7.

**3-[(2*E*)-3-(Furan-2-yl)prop-2-en-1-ylidene]pentane-2,4-dione (1g)** [64] was obtained from 3-(furan-2-yl)propenal and pentane-2,4-dione in yield of 89%. Light orange oil. ^1^H NMR (400 MHz, CDCl_3_): δ = 2.35 s (3H, Me), 2.38 s (3H, Me), 6.64 dd (1H_hetarom.,_ *J* = 3.4, 1.8 Hz), 6.55 d (1H_hetarom.,_ *J* = 3.4 Hz),6.80 d (1H, CH, *J* = 15.1 Hz), 6.94 dd (1H, CH, *J* = 15.1, 11.6 Hz), 7.12 d (1H, CH, *J* = 11.6 Hz), 7.45 d (1H_hetarom.,_ *J* = 1.8 Hz). ^13^C NMR (100 MHz, CDCl_3_): δ = 26.1, 31.5, 112.4, 113.9, 121.3, 130.7, 141.0, 142.2, 144.6, 151.7, 196.8, 202.8.

**2-(3-Phenylprop-2-en-1-ylidene)malonic acid (1h)** [65] was obtained from cinnamaldehyde and malonic acid in yield of 94%. Light orange solid. M.p. 185–187 °C. ^1^H NMR (400 MHz, (CD_3_)_2_SO): δ = 7.23 m (2H_arom._), 7.28–7.45 m (4H_arom._), 7.53–7.55 m (2H_arom._). ^13^C NMR (100 MHz, (CD_3_)_2_SO): δ = 123.3, 126.6, 127.5, 129.0, 129.7, 135.4, 142.8, 143.6, 166.0, 166.7.

**3-((2*E*)-3-Phenylprop-2-en-1-ylidene)pentane-2,4-dione (1i)** [66] was obtained from cinnamaldehyde and pentane-2,4-dione in yield of 94%. Light orange solid. M.p. 94–96 °C. ^1^H NMR (400 MHz, (CDCl_3_): δ = 2.41 s (3H, Me), 2.42 s (3H, Me), 7.04–7.23 m (3H_arom._), 7.34–7.39 m (3H), 7.48–7.50 m (2H). ^13^C NMR (100 MHz, (CDCl_3_): δ = 26.4, 31.8, 123.5, 127.9, 128.9, 130.0, 135.5, 141.5, 142.9, 145.1, 197.2, 203.0.

**1,3-Diphenyl-2-((2*E*)-3-phenylprop-2-en-1-ylidene)propane-1,3-dione (1j)** [67] was obtained from cinnamaldehyde and 1,3-diphenylpropane-1,3-dione in yield of 94%. Light orange solid. M.p. 94–96 °C. ^1^H NMR (400 MHz, CDCl_3_): δ = 6.99–7.12 m (2H), 7.30–7.32 m (3H), 7.38–7.45 m (7H), 7.51–7.55 m (2H), 7.81–7.83 m (2H), 7.92–7.94 m (2H). ^13^C NMR (100 MHz, CDCl_3_): δ = 123.7, 127.8, 128.6, 128.8, 128.9, 129.2, 129.3, 129.9, 132.5, 133.6, 135.6, 137.5, 137.8, 139.1, 144.7, 145.9, 194.4, 196.5.

**3-[(3-Phenyl-4,5-dihydrofuran-2-yl)methylidene]pentane-2,4-dione (2a)** was obtained from compound **1b** and benzene in TfOH at 80 °C for 0.25 h in yield of 70% (see Table 1). Light orange oil. ^1^H NMR (400 MHz, CDCl_3_): δ = 2.37 s (3H, Me), 2.55 s (3H, Me), 3.06 t (2H, CH_2_, *J* = 7.4 Hz), 3.34 t (2H, CH_2_, *J* = 7.4 Hz), 6.30 s (1H, CH=), 7.48 t (2H_arom._, *J* = 7.8 Hz), 7.59 t (1H_arom._, *J* = 7.8 Hz), 7.99 d (2H_arom._, *J* = 7.6 Hz). ^13^C NMR (100 MHz, CDCl_3_): δ = 14.4, 22.2, 29.2, 36.7, 106.1, 122.2, 128.1, 128.7, 133.3, 136.7, 152.7, 157.2, 194.3, 198.3. GC-MS, *m*/*z*, (I_rel._, %): 256 (35) [M]^+^, 241 (100), 213 (8), 195 (3), 177 (3), 157 (5), 151 (32). HRMS, *m*/*z* calculated for C_16_H_16_O_3_ [M+Ag]: 363.0145. Found: 363.0150.

3-{[3-(2,5-Dimethylphenyl)-4,5-dihydrofuran-2-yl]methylidene}pentane-2,4-dione (2b), 3-{[3-(2,5-dimethylphenyl)furan-2-yl]methylidene}pentane-2,4-dione (3a) and 4-hydroxy-3-{[3-(2,5-dimethylphenyl)furan-2-yl]methylidene}pent-3-en-2-one (3b), 3-{[3-(2,5-dimethylphenyl)-2,3-dihydrofuran-2-yl]methylidene}pentane-2,4-dione (4a) and 4-hydroxy-3-{[3-(2,5-dimethylphenyl)furan-2(3*H*)-ylidene]methyl}pent-3-en-2-one (4b) were obtained from compound 1b and *p*-xylene in TfOH at 0 °C for 2 h in yields of 38% (2b), 29% (3a and 3b in a ratio of 1:2.5), 20% (4a and 4b in a ratio of 4:1) (see Scheme 5).

**Compound 2b.** Yield 38%. Light orange oil. ^1^H NMR (400 MHz, CDCl_3_): δ = 2.36 s (6H, Me), 2.44 s (3H, Me), 2.54 s (3H, Me), 3.00 t (2H, CH_2_, *J* = 7.3 Hz), 3.23 t (2H, CH_2_, *J* = 7.3 Hz), 6.26 s (1H, CH=), 7.13–7.15 m (1H_arom._), 7.18–7.20 m (1H_arom._), 7.44 br.s (1H_arom._). ^13^C NMR (100 MHz, CDCl_3_): δ = 21.0, 21.1, 22.5, 29.3, 29.9, 39.5, 106.2, 122.2, 129.2, 132.1, 132.4, 135.2, 135.4, 137.5, 152.8, 157.3, 194.4, 202.5. GC-MS, *m*/*z*, (I_rel._, %): 284 (40) [M]^+^, 241 (100), 185 (72), 172 (26), 135 (22). HRMS, *m*/*z* calculated for C_18_H_20_O_3_ [M+Ag]: 391.0458. Found: 391.0464.

**Compound 3a**. ^1^H NMR (400 MHz, CDCl_3_, selected signals from the spectrum of the mixture of compounds **3a** and **3b**): δ = 3.26 d (2H, CH_2_, *J* = 7.4 Hz), 4.13 t (1H, CH, *J* = 7.4 Hz), 6.14 d (1H, *J* = 3.2 Hz), 6.39 d (1H, *J* = 3.2 Hz).

**Compound 3b**. ^1^H NMR (400 MHz, CDCl_3_, selected signals from the spectrum of the mixture of compounds **3a** and **3b**): Δ = 3.66 s (2H, CH_2_), 6.14 d (1H, *J* = 3.2 Hz), 6.39 d (1H, *J* = 3.2 Hz), 16.19 s (1H, OH).

**For the mixture of compounds 3a and 3b.** Light orange oily mixture of isomers. ^1^H NMR (400 MHz, CDCl_3_, for the mixture of compounds **3a** and **3b**): δ = 2.21 s (12H, Me), 2.35 s (6H, Me), 2.41 s (3H, Me), 2.42 (3H, Me), 7.00 br.s (1H_arom._), 7.02 br.s (1H_arom._), 7.10 br.s (1H_arom._), 7.12 br.s (1H_arom._), 7.42 br.s (1H_arom._), 7.46 br.s (1H_arom._). ^13^C NMR (100 MHz, CDCl_3_, for the mixture of compounds **3a** and **3b**): δ = 21.1, 21.2, 21.5, 21.6, 23.3, 26.9, 27.0, 29.5, 29.8, 67.2, 106.7, 107.6, 108.8, 109.3, 109.4, 127.2, 127.3, 128.2, 128.3, 130.0, 131.2, 131.3, 131.4, 135.5, 135.6, 150.8, 152.9, 153.0, 153.1, 192.0, 203.2. GC-MS, *m*/*z*, (I_rel._, %): 284 (24) [M]^+^, 241 (100), 185 (60), 172 (14), 135 (18). HRMS (for the mixture of compounds **3a** and **3b**), *m*/*z* calculated for C_18_H_20_O_3_ [M+Ag]: 391.0458. Found: 391.0464.

**Compound 4a**. ^1^H NMR (400 MHz, CDCl_3_, selected signals from the spectrum of the mixture of compounds **4a** and **4b**): δ = 3.03 dd (1H, CH, *J* = 4.6, 2.8 Hz), 4.19 d (1H, CH, *J* = 4.6 Hz), 4.32–4.34 m (1H, CH), 6.34 dd (1H, CH, *J* = 5.9, 1.8 Hz), 6.79 br.s (1H_arom._), 6.95–6.97 m (1H_arom._), 7.50–7.51 m (1H_arom._).

**Compound 4b**. ^1^H NMR (400 MHz, CDCl_3_, selected signals from the spectrum of the mixture of compounds **4a** and **4b**): δ = 3.18 d (1H, CH, *J* =3.2 Hz), 4.21–4.22 m (1H, CH), 6.49–6.50 m (1H, CH), 6.83 br.s (1H_arom._), 6.99–7.01 m (1H_arom._), 7.69–7.71 m (1H_arom._), 17.22 s (1H, OH).

**For the mixture of compounds 4a and 4b.** Light orange oily mixture of isomers. ^1^H NMR (400 MHz, CDCl_3_, for the mixture of compounds): δ =1.95 s (3H, Me), 2.07 s (3H, Me), 2.22 s (3H, Me), 2.27 s (3H, Me), 2.30 s (3H, Me), 7.04–7.07 m (2H_arom._). ^13^C NMR (100 MHz, CDCl_3_, for the mixture of compounds): δ = 19.1, 21.0, 29.7, 29.8, 46.1, 51.0, 55.1, 56.9, 66.0, 107.3, 127.0, 127.6, 128.3, 130.7, 130.9, 131.8, 132.4, 132.9, 133.7, 136.4, 136.9, 138.0, 138.9, 164.8, 166.6, 203.9, 204.5, 208.2, 208.4. HRMS (for mixture of compounds), *m*/*z* calculated for C_18_H_20_O_3_ [M+Ag]: 391.0458. Found: 391.0464.

**Methyl 3-phenyl-3-(*trans-*3-phenyl-2,3-dihydro-1-benzofuran-2-yl)propanoate (5)** was obtained from compound **1l** and benzene in TfOH at 0 °C for 0.5 h in yield of 84% or with AlCl_3_ at room temperature for 1 h in yield of 67% (Scheme 8). Light orange oil. ^1^H NMR (400 MHz, CDCl_3_): δ = 2.74 dd (1H, CH_2_, *J* = 15.7, 9.3 Hz), 3.15 dd (1H, CH_2_, *J* = 15.7, 9.3 Hz), 3.43–3.49 m (1H, CH), 3.57 s (3H, Me), 4.24 d (1H, CH, *J* = 5.1 Hz), 4.75 (1H, CH, *J* = 9.2, 5.4 Hz), 6.59–6.60 m (2H_arom._),6.83–6.93 m (3H_arom._), 7.10–7.11 m (3H_arom._), 7.18–7.20 m (3H_arom._), 7.26–7.29 m (3H_arom._). ^13^C NMR (100 MHz, CDCl_3_): δ = 38.3, 47.7, 51.7, 52.7, 94.1, 109.9, 121.2, 125.7, 126.7, 127.6, 127.8, 128.6, 128.8, 128.9, 130.2, 139.6, 143.5, 159.3, 172.6. HRMS, *m*/*z* calculated for C_24_H_22_O_3_[M+H]: 359.1642. Found: 359.1638.

Methyl 2-((5a***S(R)***,6***R(S)***,10b***S(R)***)-7,10-dimethyl-5a,10b-dihydro-6***H***-indeno [2,1-***b***]benzofuran-6-yl)acetate(6a) and methyl 2-((5a***S(R)***,6***S(R)***,10b***S(R)***)-7,10-dimethyl-5a,10b-dihydro-6***H***-indeno [2,1-***b***]benzofuran-6-yl)acetate (6b) were obtained from compound 1l and *p*-xylene in TfOH at 0 °C for 0.5 h in yields of 42% (6a) and 30% (6b); or with AlCl_3_ at room temperature for 1 h in yields of 28% (6a) and 16% (6b) (Scheme 8).

**Compound 6a**. ^1^H NMR (400 MHz, CDCl_3_, selected signals from the spectrum of the mixture of compounds **6a** and **6b**): δ = 2.31 s (3H, Me), 2.31–2.37 m (1H, CH_2_), 2.48 s (3H, Me), 2.78 dd (1H, CH_2_, *J* = 12.7, 2.6 Hz), 3.71 s (3H, Me), 4.04 dd (1H, CH, *J* = 8.7, 2.6 Hz), 4.99 d (1H, CH, *J* = 5.6 Hz), 5.36 dd (1H, CH, *J* = 5.6, 0.5 Hz), 6.74–6.76 m (1H_arom._), 6.95–6.97 m (1H_arom._), 7.06–7.09 m (1H_arom._), 7.49–7.50 m (1H_arom._). ^13^C NMR (100 MHz, CDCl_3_, selected signals from the spectrum of the mixture of compounds **6a** and **6b**): δ = 18.6, 20.4, 37.6, 47.1, 52.0, 52.1, 95.5, 109.9, 120.2, 126.3, 131.7, 132.2, 140.6, 141.6, 160.0, 172.2. GC-MS, *m*/*z*, (I_rel._, %): 308 (4) [M]^+^, 277 (14), 263 (22), 249 (36), 235 (100).

**Compound 6b**. ^1^H NMR (400 MHz, CDCl_3_, selected signals from the spectrum of the mixture of compounds **6a** and **6b**): δ = 2.14 dd (1H, CH_2_, *J* = 13.4, 9.8 Hz), 2.26 s (3H, Me), 2.55–2.58 m (1H, CH_2_), 2.59 s (3H, Me), 3.80 s (3H, Me), 4.19–4.23 m (1H, CH), 5.07 d (1H, CH, *J* = 7.2 Hz), 5.70 t (1H, CH, *J* = 7.0 Hz), 6.77–6.78 m (1H_arom._), 6.81–6.86 m (1H_arom._), 6.94 br.s (1H_arom._), 7.03–7.05 m (1H_arom._), 7.12–7.15 m (1H_arom._), 7.35–7.37 m (1H_arom._). ^13^C NMR (100 MHz, CDCl_3_, selected signals from the spectrum of the mixture of compounds **6a** and **6b**): δ = 18.7, 20.2, 34.6, 45.7, 51.6, 51.8, 86.6, 110.0, 121.1, 125.0, 128.8, 131.6, 131.7, 140.8, 141.7, 160.2, 173.3. GC-MS, *m*/*z*, (I_rel._, %): 308 (2) [M]^+^, 277 (16), 263 (17), 249 (30), 235 (100).

**For the mixture of compounds 6a and 6b.** Light orange oily mixture of isomers. ^1^H NMR (400 MHz, CDCl_3_, for the mixture of compounds): δ = 6.81–6.86 m (2H_arom._), 6.94 br.s (2H_arom._). ^13^C NMR (100 MHz, CDCl_3_, for the mixture of compounds): δ = 128.5, 129.6. HRMS (for the mixture of compounds), *m*/*z* calculated for C_20_H_20_O_3_ [M+H]: 309.1485. Found: 309.1483.

1,3-Diphenylindene (7), 1-phenylindene (8), 3-phenylindanone (9), 2,2-diphenylethanal (existing in enolic form as 2,2-diphenylethen-1-ol) (10) were obtained from compound 1h-j and benzene in TfOH (see the reaction temperature, time, and yields of the compounds in Table 2).

**1,3-Diphenylindene (7)** [54]. Light orange solid. M.P. 85–87 °C. ^1^H NMR (500 MHz, CDCl_3_): δ = 4.72 d (1H, CH, *J* = 1.7 Hz), 6.65 d (1H, =CH, *J* = 2.2 Hz), 7.18–7.25 m (4H_arom._), 7.28–7.34 m (4H_arom._), 7.39–7.42 m (1H_arom._), 7.46–7.49 m (2H_arom._), 7.60–7.61 m (1H_arom._), 7.66–7.68 m (2H_arom._). ^13^C NMR (125 MHz, CDCl_3_): δ = 55.5, 120.7, 124.5, 125.7, 126.8, 127.0, 127.9, 128.0, 128.1, 128.8, 128.9, 135.8, 136.5, 139.7, 143.3, 144.8, 149.4.

**1-Phenylindene (8)** [68]. Light orange oil. ^1^H NMR (400 MHz, CDCl_3_): δ = 3.52 d (2H, CH_2_, *J* = 1.8 Hz), 6.59 t (1H, =CH, *J* = 1.8 Hz), 7.27–7.29 m (1H_arom._), 7.32–7.35 m (1H_arom._), 7.37–7.40 m (1H_arom._), 7.45–7.48 m (2H_arom._), 7.54–7.56 m (1H_arom._), 7.60–7.62 m (3H_arom._). ^13^C NMR (100 MHz, CDCl_3_): δ = 38.3, 120.5, 124.3, 125.0, 126.3, 127.7, 127.9, 128.7, 131.1, 136.3, 144.0, 144.9, 145.3.

**3-Phenylindanone (9)** [69]. Light orange oil. ^1^H NMR (400 MHz, CDCl_3_): δ = 2.70 dd (1H, CH_2_, *J* = 3.9, 19.2 Hz), 3.24 dd (1H, CH_2_, *J* = 8.1, 19.2 Hz), 4.58 dd (1H, CH, *J* = 3.9, 8.1 Hz), 7.12–7.14 m (2H_arom._), 7.25–7.27 m (2H_arom._), 7.28–7.32 (3H_arom._), 7.40–7.44 m (1H_arom._), 7.56–7.59 (1H_arom._), 7.81–7.82 (1H_arom._). ^13^C NMR (100 MHz, CDCl_3_): δ = 44.6, 47.0, 123.6, 127.0, 127.1, 127.8, 128.0, 129.1, 135.2, 136.9, 143.8, 158.1, 206.2.

**2,2-Diphenylethanal (existing in enolic form as 2,2-diphenylethen-1-ol) (10)** [70]. Light orange oil. ^1^H NMR (400 MHz, CDCl_3_): δ = 6.87 s (1H, CH), 7.48–7.58 m (6H_arom._), 7.99–8.01 m (4H_arom._), 16.85 c (1H, OH). ^13^C NMR (100 MHz, CDCl_3_): δ = 93.3; 127.3; 128.8; 132.6; 135.8; 185.9.

***Cis-*1,3-diphenylindane (11) and 1,1-diphenylindane (12)** were obtained from compound **1h-j** and benzene with zeolite CBV-500 or CBV-720 (see the reaction temperature, time, and yields of the compounds in Table 3).

***Cis-*1,3-Diphenylindane (11)** [71]. ^1^H NMR (400 MHz, CDCl_3_, selected signals from the spectrum of the mixture of compounds **11** and **12**): δ = 2.15–2.24 m (1H, CH), 2.98–3.05 m (1H, CH), 4.38–4.43 m (2H, CH_2_). GC-MS, *m*/*z*, (I_rel._, %): 270 (60) [M]^+^, 255 (20), 192 (100), 178 (45), 165 (24).

**1,1-Diphenylindane (12)** [72]. ^1^H NMR (400 MHz, CDCl_3_, selected signals from the spectrum of the mixture of compounds **11** and **12**): δ = 2.66 t (2H, CH_2_, *J* = 7.2 Hz), 4.61 t (2H, CH_2_, *J* = 7.2 Hz). GC-MS, *m*/*z*, (I_rel._, %): 270 (45) [M]^+^, 255 (16), 192 (100), 178 (42), 165 (16).

**For the mixture of compounds 11 and 12.** White solid. M.p. of mixture of isomers 78–80 °C. ^1^H NMR (400 MHz, CDCl_3_, for the mixture of compounds): δ = 7.00–7.02 m (2H_arom._), 7.16–7.20 m (2H_arom._), 7.21–7.23 m (H_arom._), 7.26–7.29 m (4H_arom._), 7.31–7.40 (14H_arom._). ^13^C NMR (100 MHz, CDCl_3_, for the mixture of compounds): δ = 46.5, 48.0, 50.0, 50.8, 124.7, 125.2, 126.4, 126.6, 126.8, 127.2, 127.9, 128.52, 128.54, 128.6, 144.4, 145.4, 146.8, 147.2. HRMS (for the mixture of compounds), *m*/*z* calculated for C_21_H_18_ [M+Ag]: 377.0454. Found: 377.0460.

**Cation *Ah-2D*** generated at the protonation of compound **1h** in D_2_SO_4_. ^1^H NMR (400 MHz, D_2_SO_4_): δ = 7.60–7.61 m (2H_arom._), 7.72–7.74 m (1H_arom._), 7.89–7.91 (2H_arom._), 8.15 d (1H, CH, *J* = 14.4 Hz), 8.26 t (1H, CH, *J* = 13.4 Hz), 8.85 d (1H, CH, *J* = 12.2 Hz). ^13^C NMR (100 MHz, D_2_SO_4_): δ = 101.1, 125.6, 130.4, 130.5, 133.1, 134.5, 134.6, 137.4, 170.2, 171.7, 176.2, 178.1.

## 4. Conclusions

The series of furans and benzenes, containing ethenyl and conjugated butadienyl substituents with two geminal electron-withdrawing groups, has been synthesized. A novel method for synthesis of 2,3-dihydrofurans and 2,3-dihydrobenzofurans has been developed based on reactions of ethenyl-substituted furans with arenes in TfOH. The obtained compounds have shown moderate antimicrobial activity against the yeast-like fungi *Candida albicans*. In addition, these compounds suppress Escherichia coli and *Staphylococcus aureus*.

## Figures and Tables

**Table 1:** Reaction of furan **1b** with benzene under the action of Brønsted or Lewis acids leading to compound **2a**.

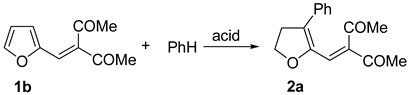				
Entry	Reaction Conditions			Yield of 2a, %
	Acid (Equiv.)	Temperature, °C	Time, h	
1	H_2_SO_4_(16)	r.t.	24	no reaction
2	H_2_SO_4_ (16)	60	0.25	oligomerization
3	AlCl_3_(5.5)	r.t.	1	oligomerization
4	TfOH (16)	r.t.	1	12
5	TfOH (16)	r.t.	12	61
6	TfOH (16)	80	0.25	70
7	TfOH (16)	80	1	44
8	TfOH (16)	80	2	9

**Table 2:** Reaction of compounds **1h–j** with benzene in TfOH leading to compounds **7–10**.

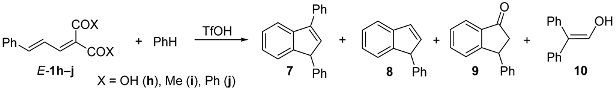							
Entry	Starting Compound *E*-1h–j	Reaction Conditions		Yields of Compounds 7–10, %			
1	*E*-**1h**	room temperature	12	40	32	24	-
2	*E*-**1h**	room temperature	24	37	31	14	-
3	*E*-**1h**	80	1	-	-	-	17
4	*E*-**1h**	80	2	oligomers			
5	*E*-**1i**	room temperature	12	45	30	19	-
6	*E*-**1i**	80	1	-	-	-	31
7	*E*-**1j**	room temperature	12	35	42	12	-
8	*E*-**1j**	80	1	-	-	-	58

**Table 3:** Reaction of compounds *E*-**1h**–**j** with benzene under the action of acidic zeolites CBV-500 or CBV-720 at 150 °C for 100 h leading to compounds **11** and **12**.

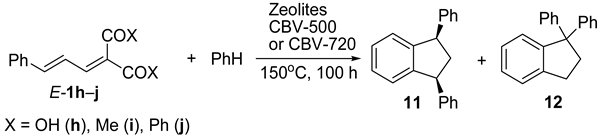			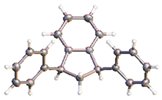 X-ray of **11**	
**Entry**	**Starting Compound** ***E*-1h–j**	**Zeolite**	**Yields of Compounds 11 and 12, %**	
			**11**	**12**
1	*E*-**1h**	CBV-500	28	29
2	*E*-**1h**	CBV-720	23	24
3	*E*-**1i**	CBV-500	35	36
4	*E*-**1i**	CBV-720	34	37
5	*E*-**1j**	CBV-500	32	34
6	*E*-**1j**	CBV-720	29	33

**Table 4:** ^1^H and ^13^C NMR data of starting compound *E*-**1h** and its dideuterated form ***Ah–2D*** generated in D_2_SO_4_.

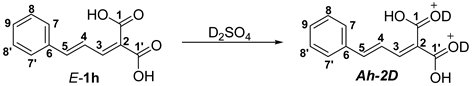						
Compound/Cation	^13^C NMR, δ, ppm					
	C^1^	C^1^′	C^2^	C^3^	C^4^	C^5^
**1h** in (CD_3_)_2_SO	166.7	166.0	126.6	143.6	123.3	142.8
***Ah–2D*** in D_2_SO_4_	178.1	176.2	101.1	170.2	125.6	171.4
Δδ = δ**_Ah_** − δ**_1h_**	11.4	10.2	−25.5	26.6	2.3	28.6
	C^6^	C^7^	C^7^′	C^8^	C^8^′	C^9^
**1h** in (CD_3_)_2_SO	135.4	127.5	127.5	129.0	129.0	129.7
***Ah–2D*** in D_2_SO_4_	133.1	134.6	134.5	130.5	130.4	137.4
Δδ = δ**_Ah_** − δ**_1h_**	−2.3	7.1	7.0	1.5	1.4	7.7
	^1^H NMR, Δ, ppm					
	H^3^	H^4^	H^5^	H^7,7^′*_ortho-_*	H^8,8^′*_meta-_*	H^9^*_para-_*
**1h** in (CD_3_)_2_SO	7.23	7.40	7.44	7.53	7.39	7.23
***Ah–2D*** in D_2_SO_4_	8.85	8.26	8.15	7.90	7.60	7.73
Δδ = δ**_Ah_** − δ**_1h_**	1.62	0.86	0.71	0.37	0.21	0.5

**Table 5:** Evaluation of the antimicrobial activity of the obtained compounds **1–12**.

Compound	Minimum Inhibitory Concentration (MIC), μg/mL		
	*Escherichia coli* ATCC 25922	*Staphylococcus aureus* ATCC 6538-P	*Candida albicans* ATCC 10231
**1a**	128	128	64
**1b**	128	128	64
**1c**	128	128	64
**1d**	128	256	64
**1e**	128	128	64
**1f**	128	128	64
**1g**	256	256	64
**1h**	128	128	128
**1i**	128	128	64
**1j**	128	256	64
**1l**	128	>256 (not found)	64
**2a**	128	256	64
**2b**	128	128	64
**3a + 3b**	128	128	64
**5**	>256 (not found)	>256 (not found)	64
**6a + 6b**	>256 (not found)	>256 (not found)	64
**7**	128	128	64
**8**	>256 (not found)	>256 (not found)	64
**9**	>256 (not found)	>256 (not found)	64
**10**	>256 (not found)	>256 (not found)	32
**11 +12**	>256 (not found)	>256 (not found)	64

## Data Availability

The original contributions presented in this study are included in the article/Supplementary Material. Further inquiries can be directed to the corresponding author.

## Supplementary Materials

The following supporting information can be downloaded at: https://www.mdpi.com/article/10.3390/molecules31183350/s1. It contains ^1^H, ^13^C, and ^19^F NMR spectra of compounds **1**–1**2**, and cation ***Ah***, X-ray data for compound **11**; data of DFT calculations of compound **1b**, and cations ***A****-**E***, ***G***, ***J***, ***K***, ***K***-***H^+^***, water H_2_O and H_3_O^+^; study of biological activity.
