# Supplementary material for: Synthesis of Furan and Benzene Derivatives Containing Unsaturated Substituents with Electron-Withdrawing Groups, and Analysis of Their Antimicrobial Activity
Source: Molecules. 2026 Sep 21;31(18):3350. doi: 10.3390/molecules31183350 (PMC13610081; doi:10.3390/molecules31183350)
Supplement: Supplementary file 1 [file molecules-31-03350-s001.zip › molecules-4537595-supplementary.pdf]

Synthesis of furan and benzene derivatives containing unsaturated substituents with electron-withdrawing groups, and analysis of their antimicrobial activity

Mikhail V. Kalyaev,[a,b] Irina A. Boyarskaya,[a] Alexander Yu. Ivanov,[c] Dar'ya V. Spiridonova,[d] Kristina E. Borovkova,[e] Sofia A. Brevnova,[e] Artem O. Taraskin,[e] Karina S. Gromozdova,[e] Aleksander V. Vasilyev*[a,f]

[a]Department of Organic Chemistry, Institute of Chemistry, Saint Petersburg State University, Universitetskaya nab., 7/9, Saint Petersburg, 199034, Russia
[b]Ufa State Petroleum Technical University, ul. Kosmonavtov 1, 450064, Ufa, Russia
[c]Center for Magnetic Resonance, Research Park, Saint Petersburg State University, Universitetskiy pr., 26, Saint Petersburg, Petrodvoretz, 198504, Russia
[d]Research Center for X-ray Diffraction Studies, Research Park, Saint Petersburg State University, Universitetskiy pr. 26, Saint Petersburg, Petrodvoretz, 198504, Russia
[e]Research and manufacturing company «Home of Pharmacy», Zavodskaya st., 3-245, Kuzmolovskiy t.s., Leningrad oblast, 188663, Russia
[f]Department of Chemistry, Saint Petersburg State Forest Technical University, Institutsky per., 5, Saint Petersburg, 194021, Russia

*Corresponding author: A.V. Vasilyev; e-mails: *aleksvasil@mail.ru; a.vasilyev@spbu.ru*

Contents

1. ^{1}H, ^{13}C, and ^{19}F NMR spectra of compounds **1**-1**2**, and cation **Ah** ………….………………S2

2. X-ray data for compound **11**…………………………………………………………S27

3. Data of DFT calculations of compound **1b**, and cations **A-E, G, J, K, K-H^{+},**

water H_2O and H_3O^{+}……………………………………………………………….S28

4. Study of biological activity…………………………………………………….…S58

1. ^{1}H, ^{13}C, and ^{19}F NMR spectra of compounds 1-12, and cation Ah

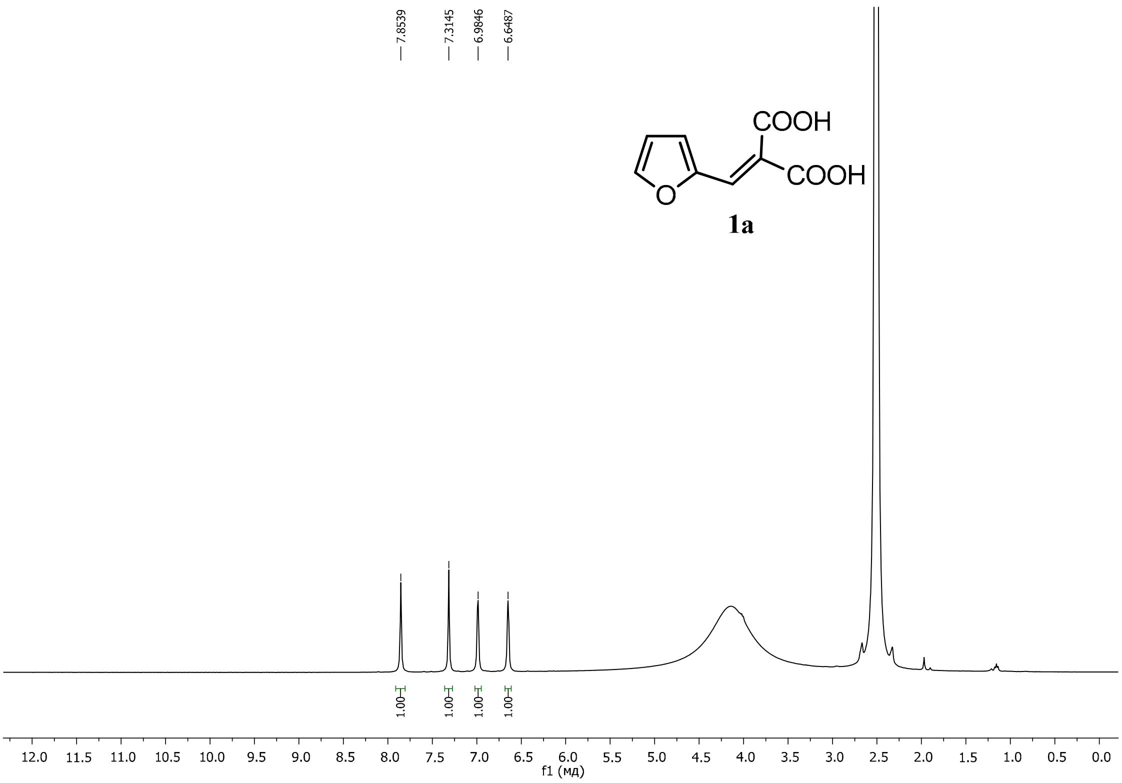

Figure S1. ^{1}H NMR spectrum of compound **1a** (400 MHz, $(CD_3)_2SO$).

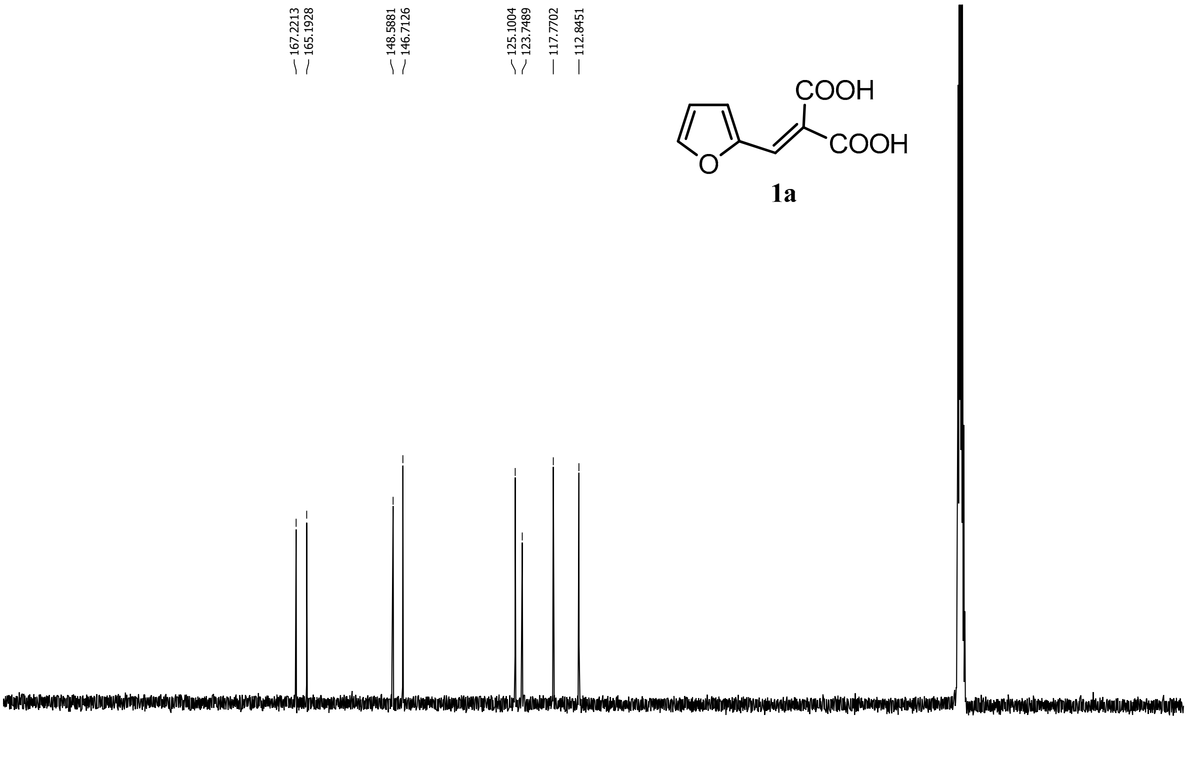

Figure S2. ^{13}C NMR spectrum of compound **1a** (100 MHz, $(CD_3)_2SO$).

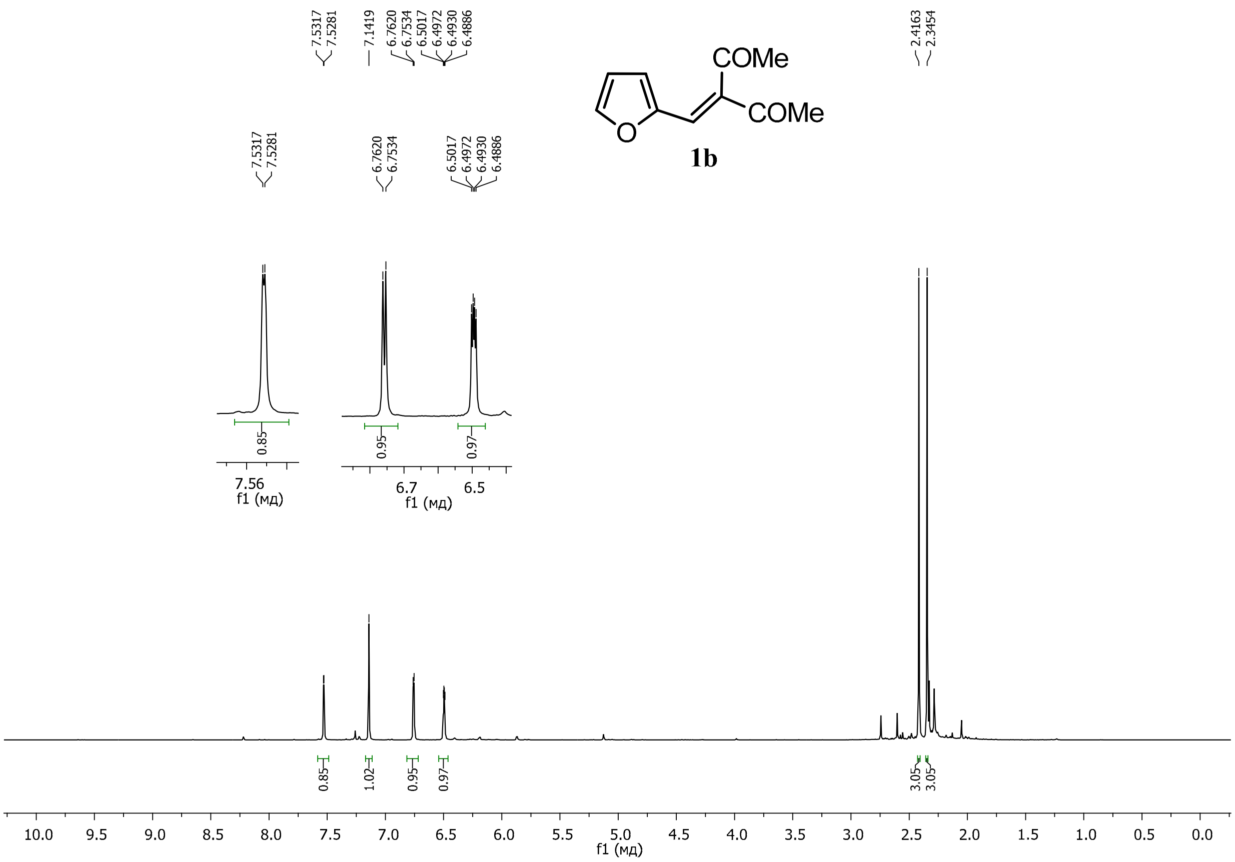

Figure S3. ^{1}H NMR spectrum of compound **1b** (400 MHz, $CDCl_3$).

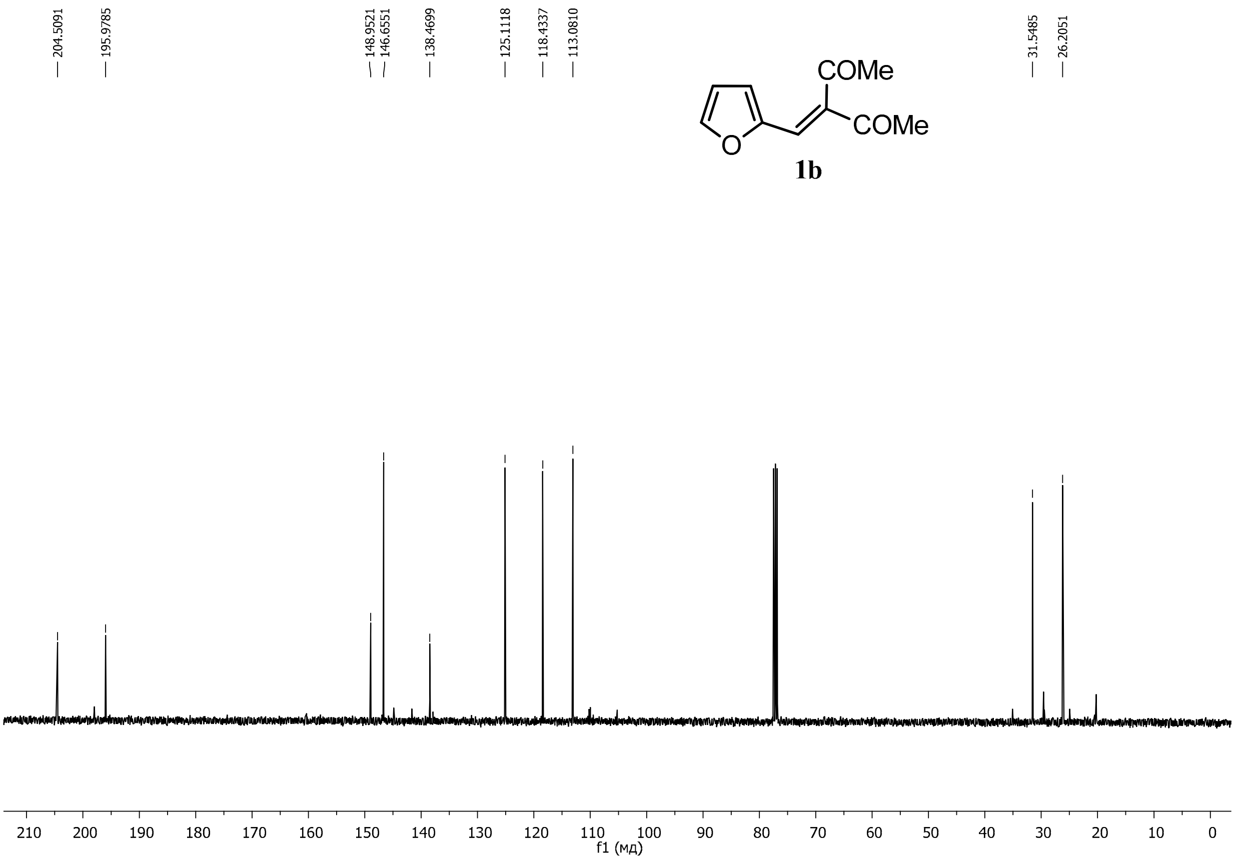

Figure S4. ^{13}C NMR spectrum of compound **1b** (100 MHz, $CDCl_3$).

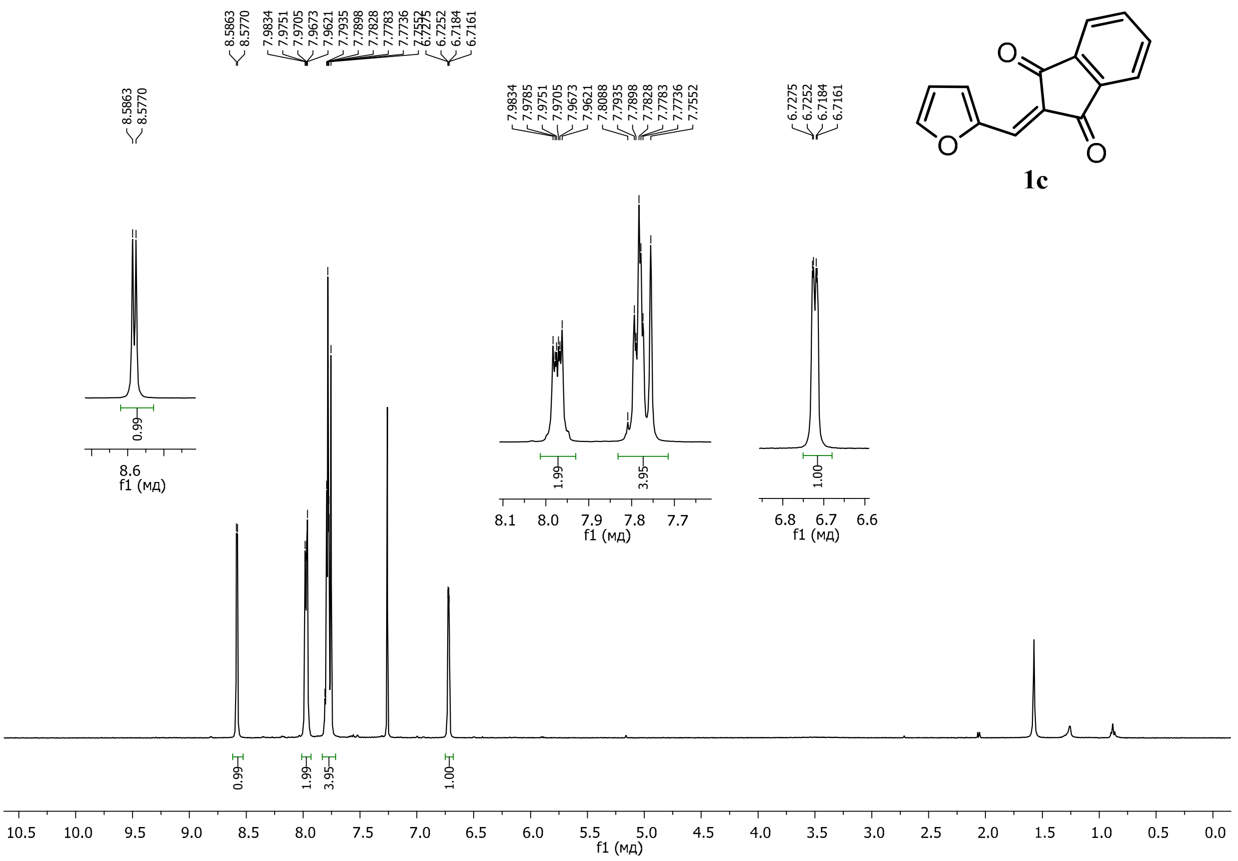

Figure S5. ^{1}H NMR spectrum of compound **1c** (400 MHz, $CDCl_3$).

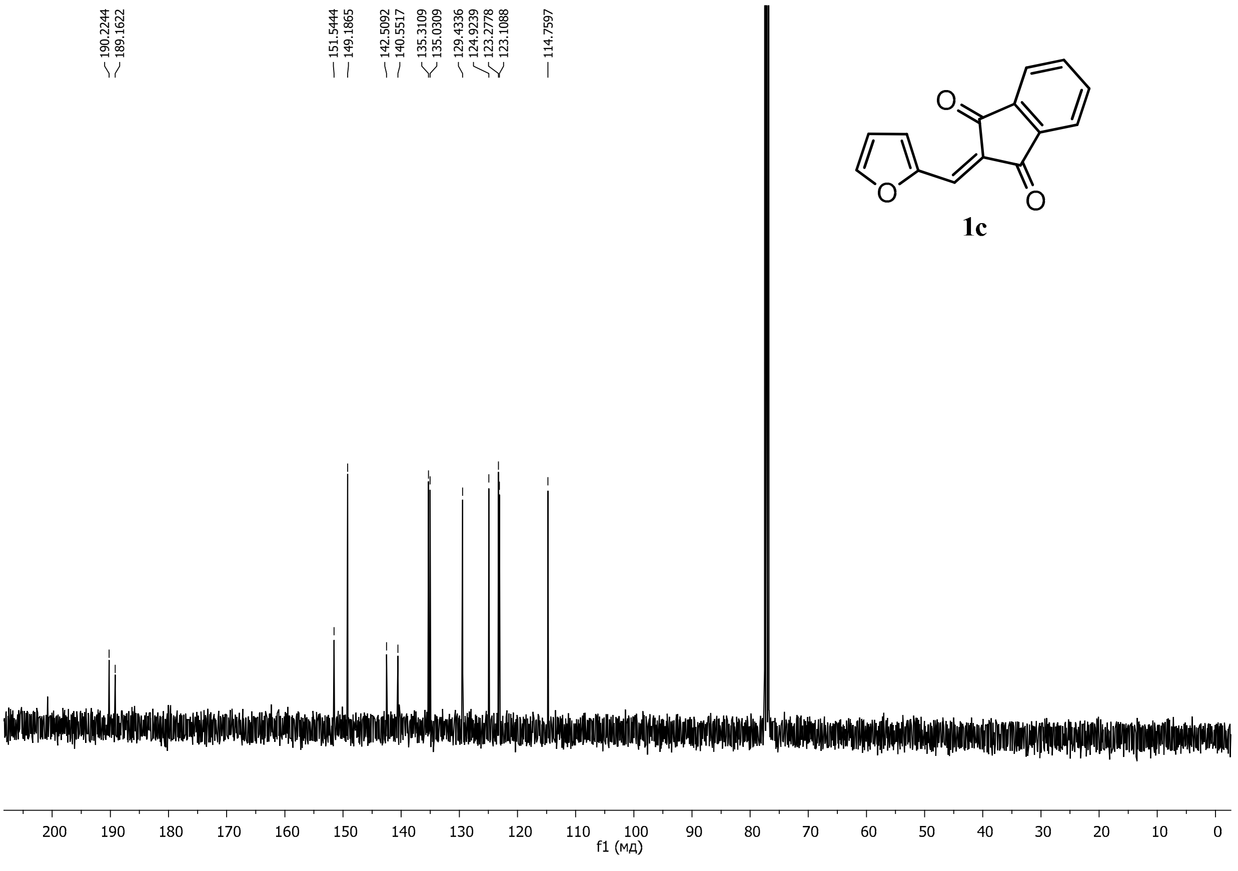

Figure S6. ^{13}C NMR spectrum of compound **1c** (100 MHz, $CDCl_3$).

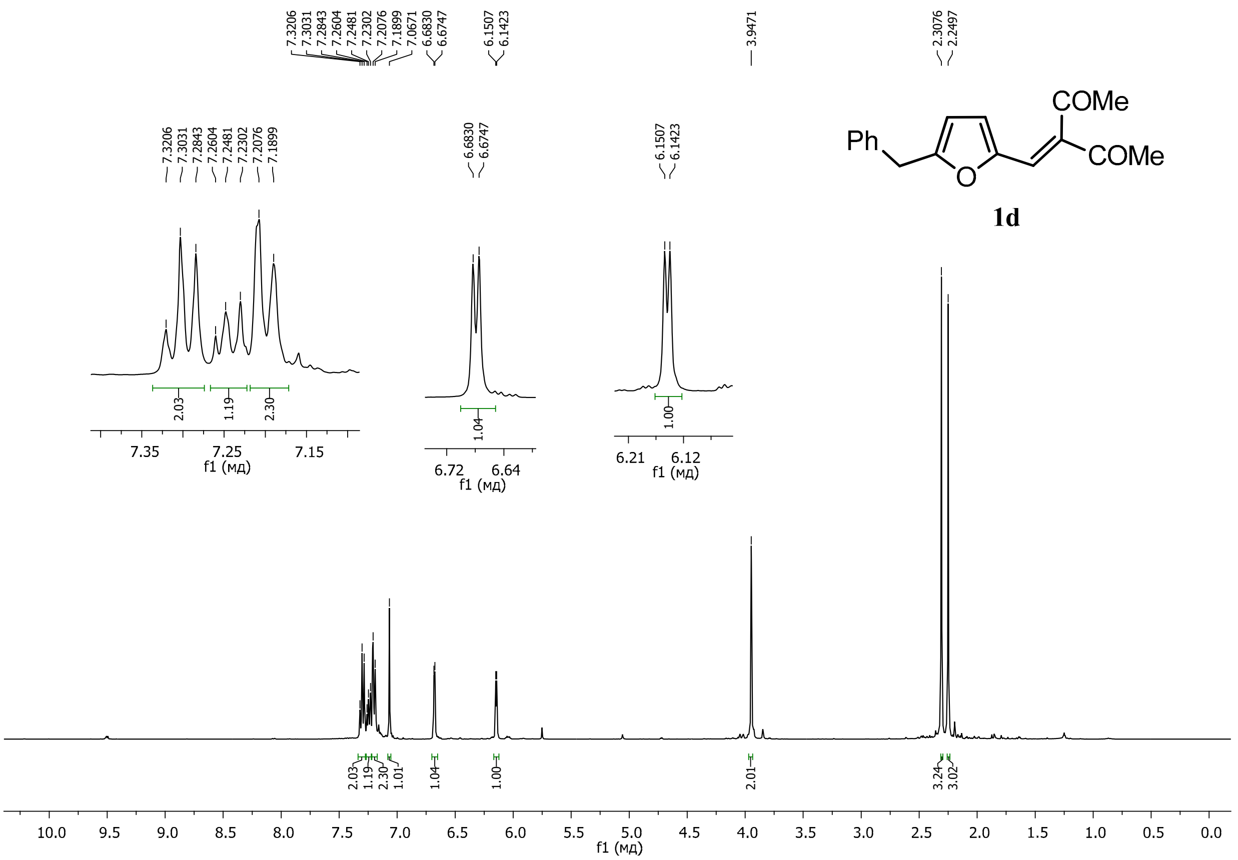

Figure S7. ^{1}H NMR spectrum of compound **1d** (400 MHz, $CDCl_3$).

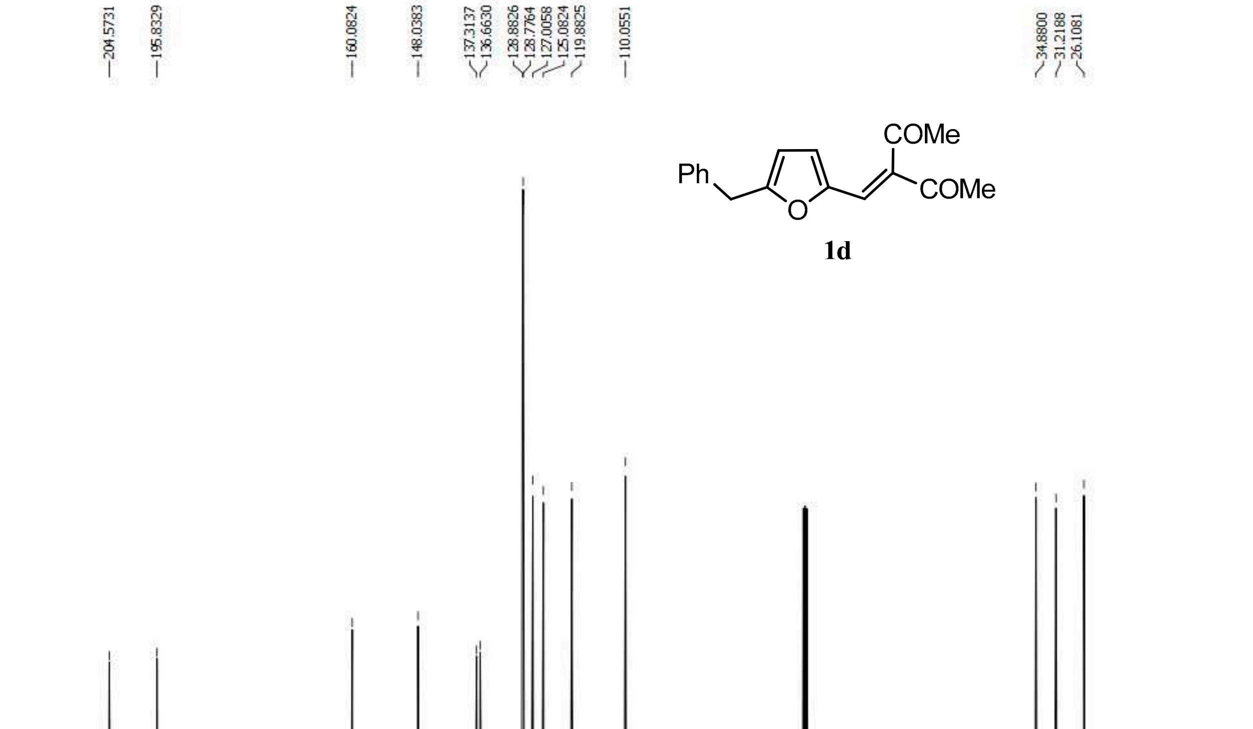

Figure S8. ^{13}C NMR spectrum of compound **1d** (100 MHz, $CDCl_3$).

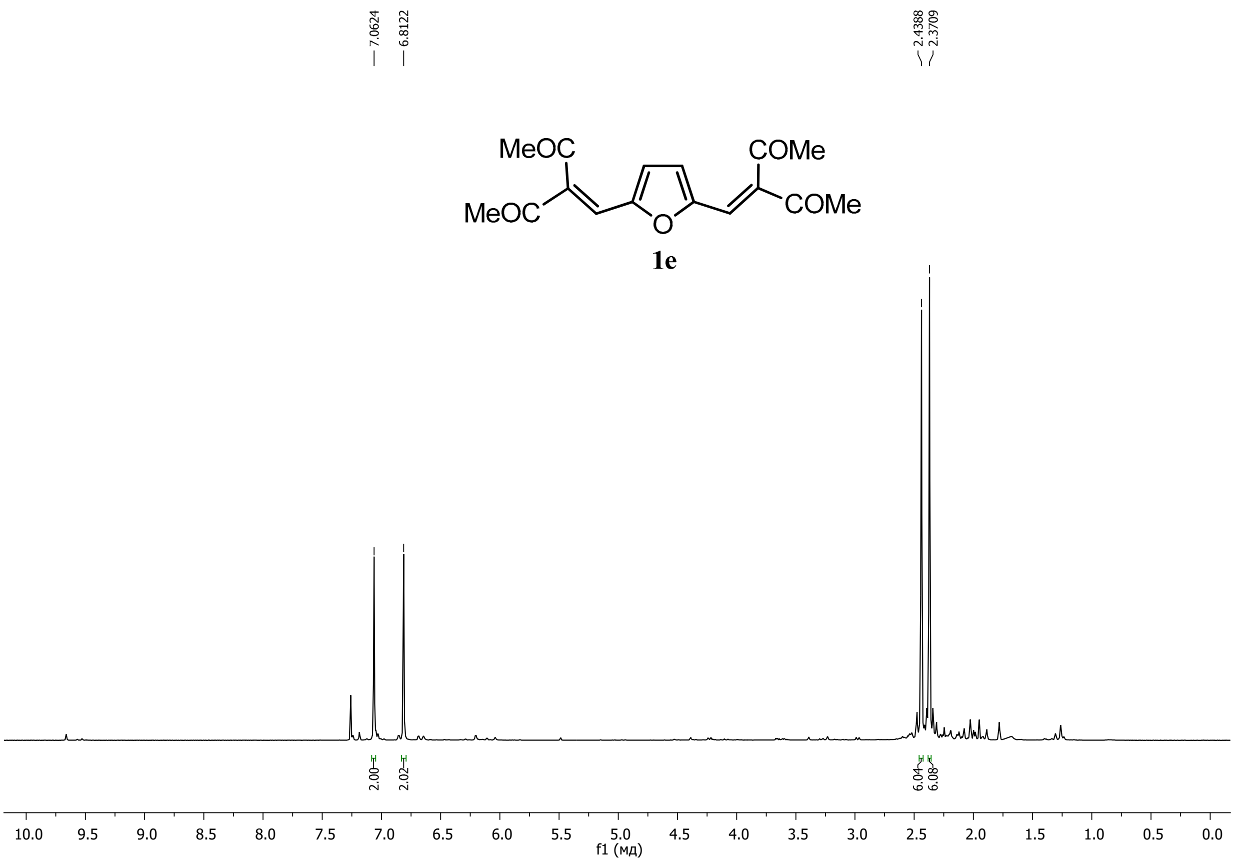

Figure S9. ^{1}H NMR spectrum of compound **1e** (400 MHz, $CDCl_3$).

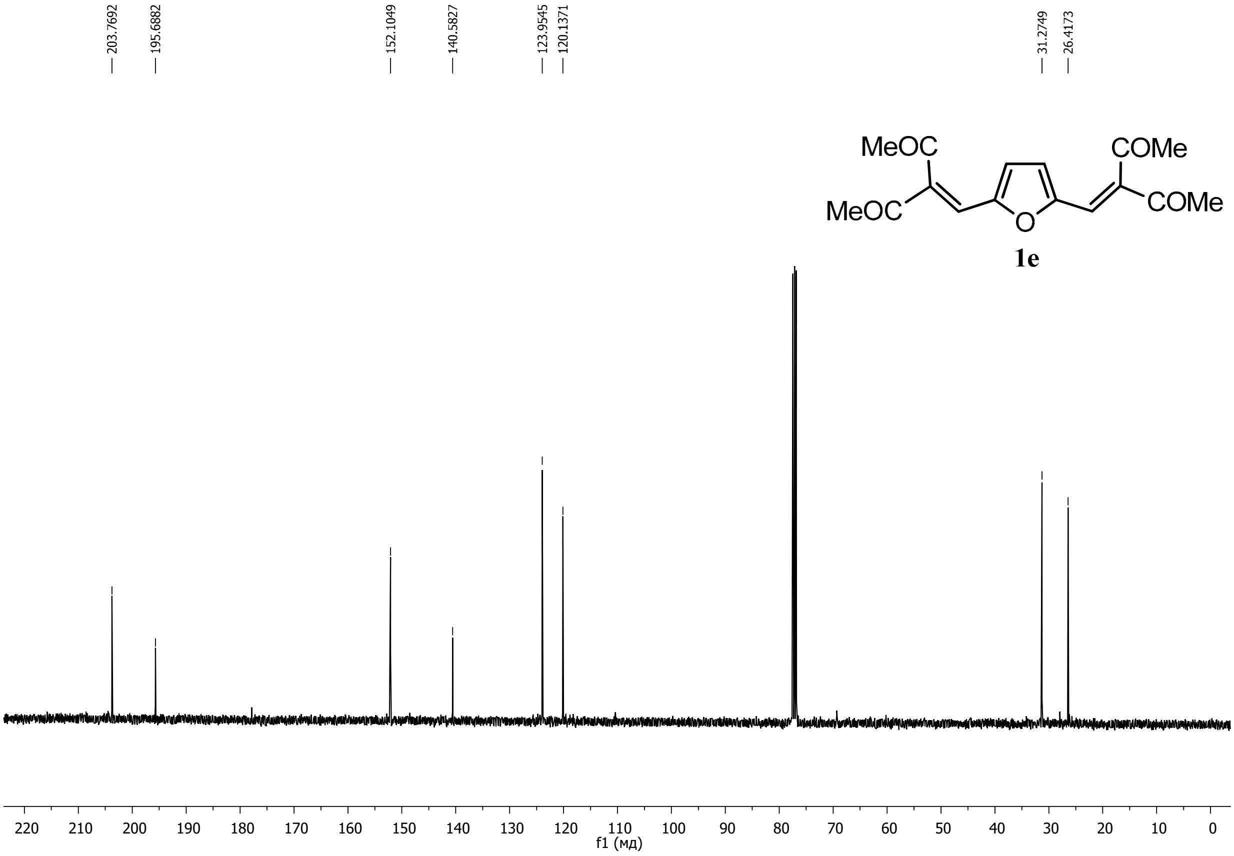

Figure S10. ^{13}C NMR spectrum of compound **1e** (100 MHz, $CDCl_3$).

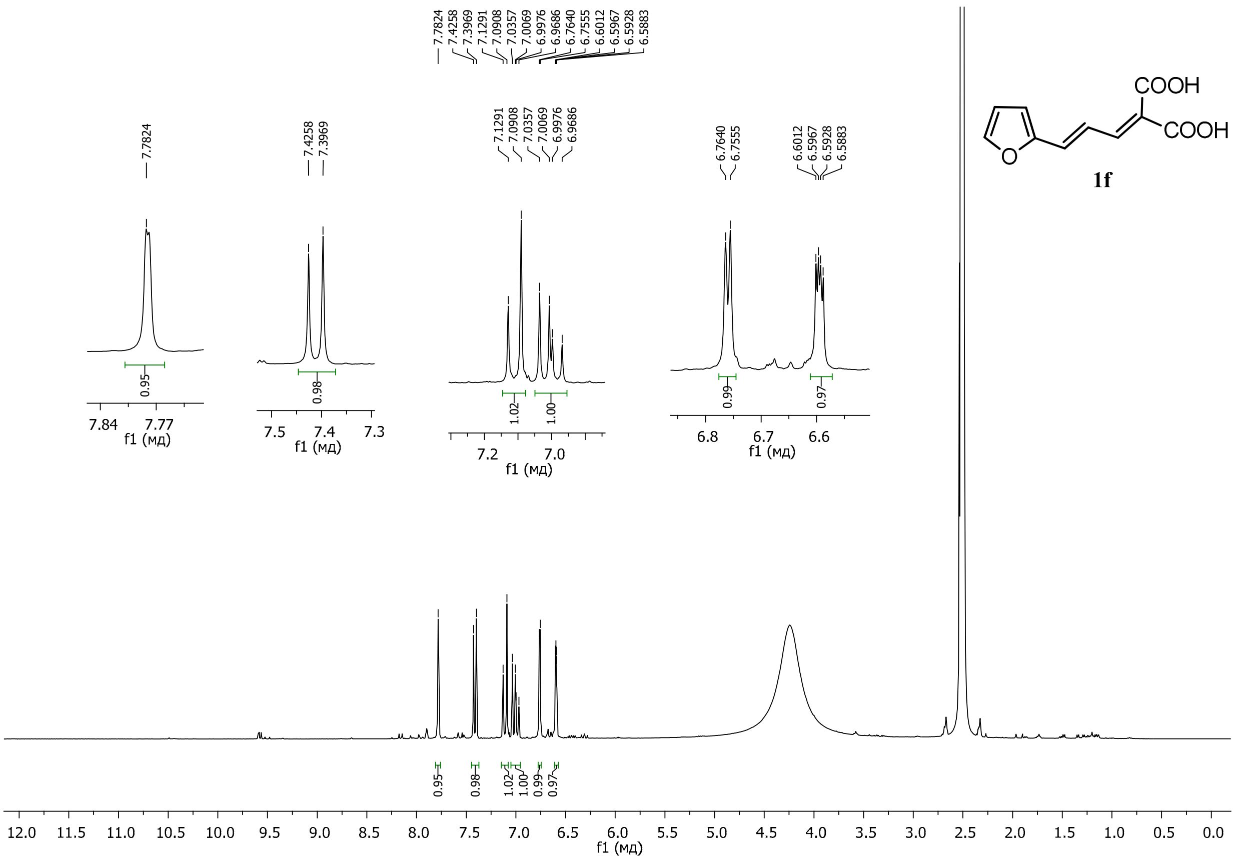

Figure S11. ^{1}H NMR spectrum of compound **1f** (400 MHz, $(CD_3)_2SO$).

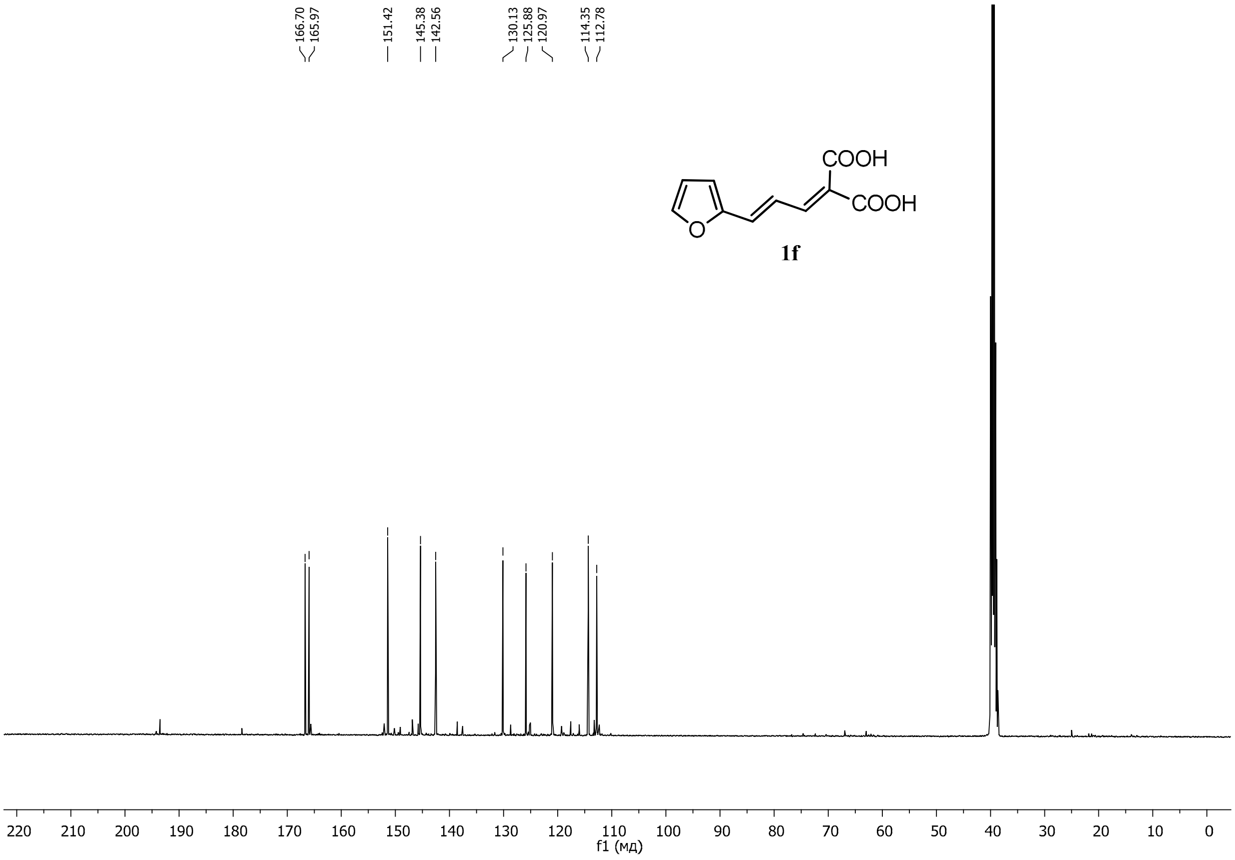

Figure S12. ^{13}C NMR spectrum of compound **1f** (100 MHz, $(CD_3)_2SO$).

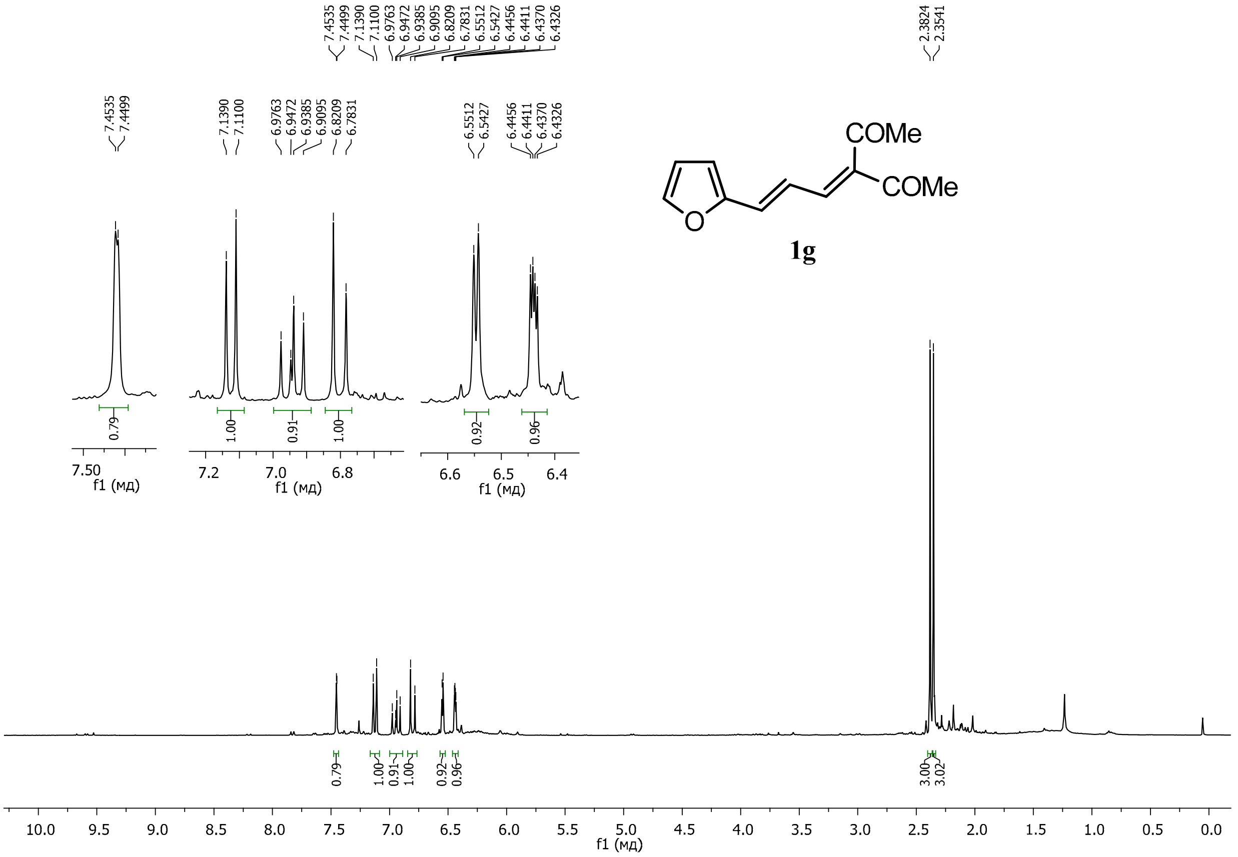

Figure S13. ^{1}H NMR spectrum of compound **1g** (400 MHz, ($CDCl_3$).

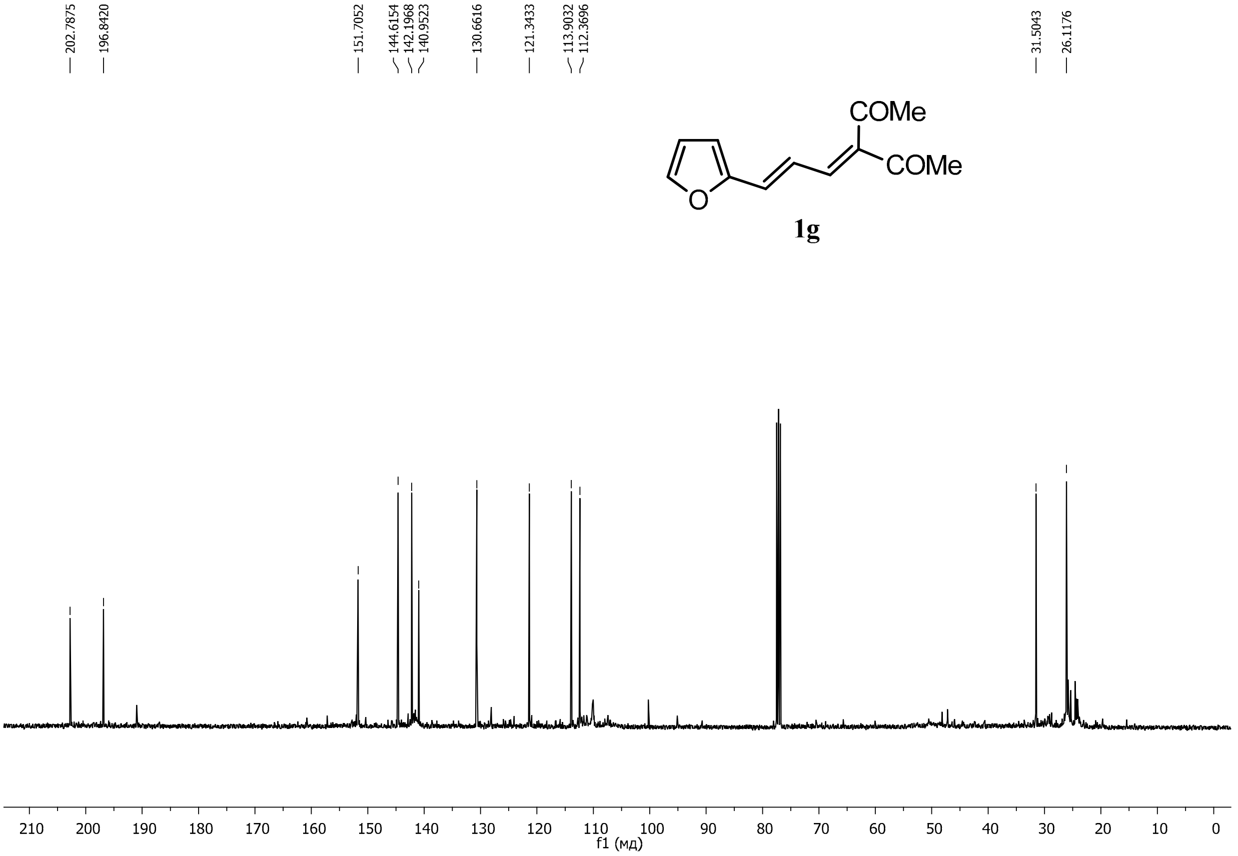

Figure S14. ^{13}C NMR spectrum of compound **1g** (100 MHz, ($CDCl_3$).

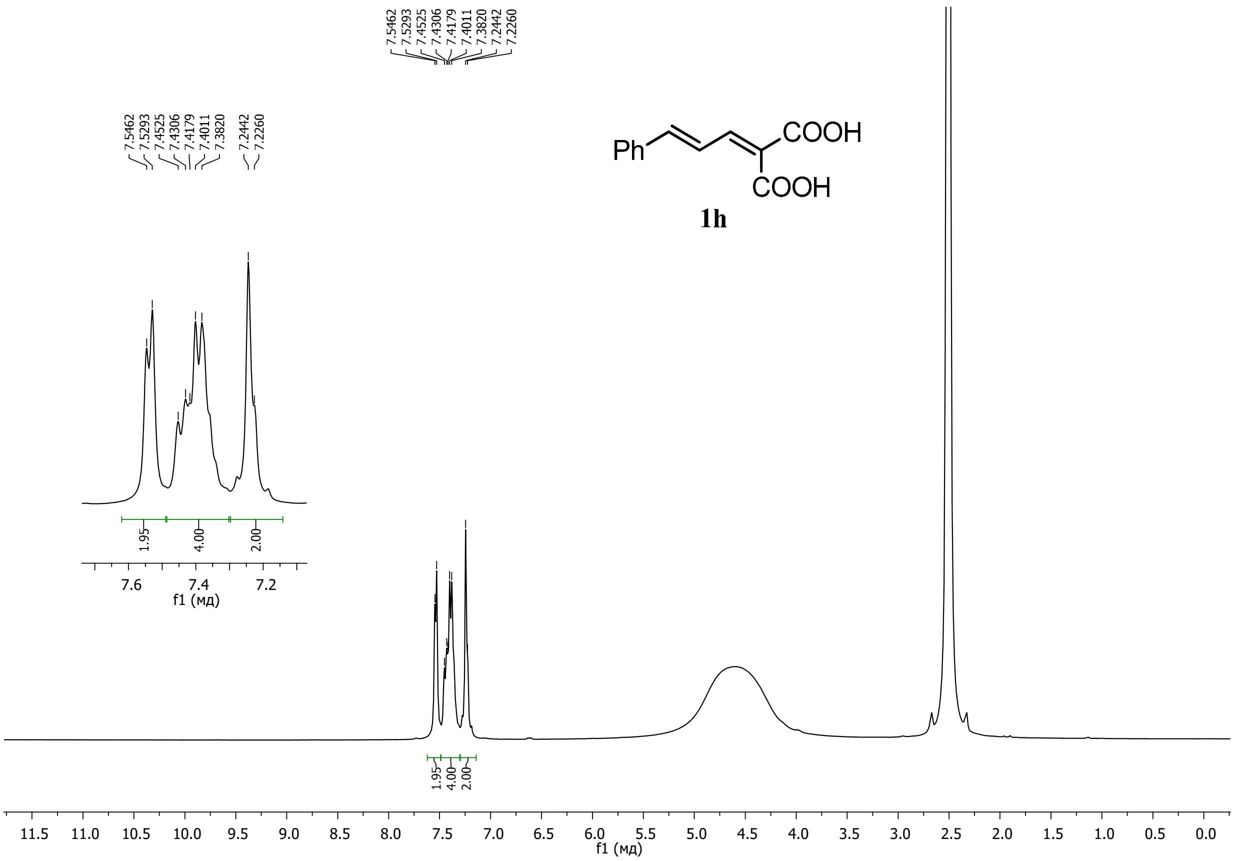

Figure S15. ^{1}H NMR spectrum of compound **1h** (400 MHz, $(CD_3)_2SO$).

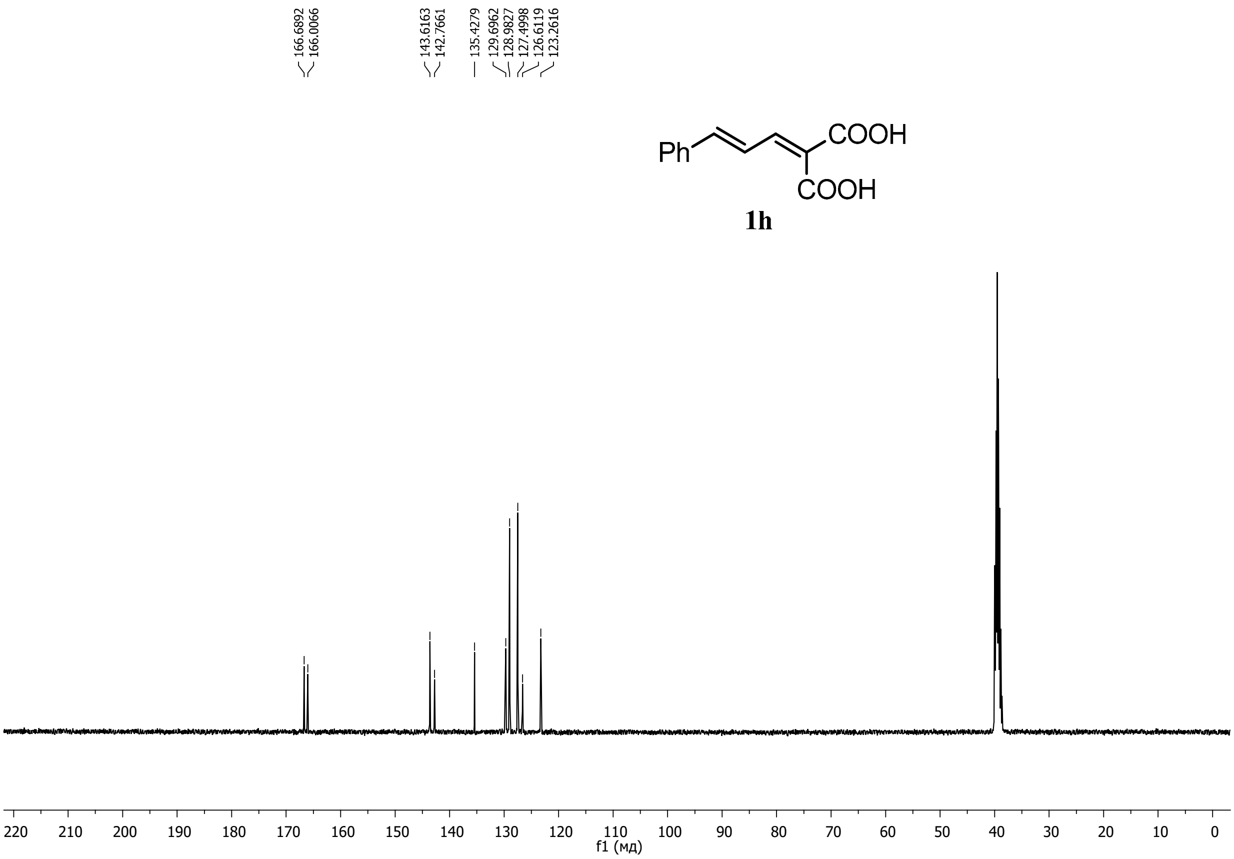

Figure S16. ^{13}C NMR spectrum of compound **1h** (100 MHz, $(CD_3)_2SO$).

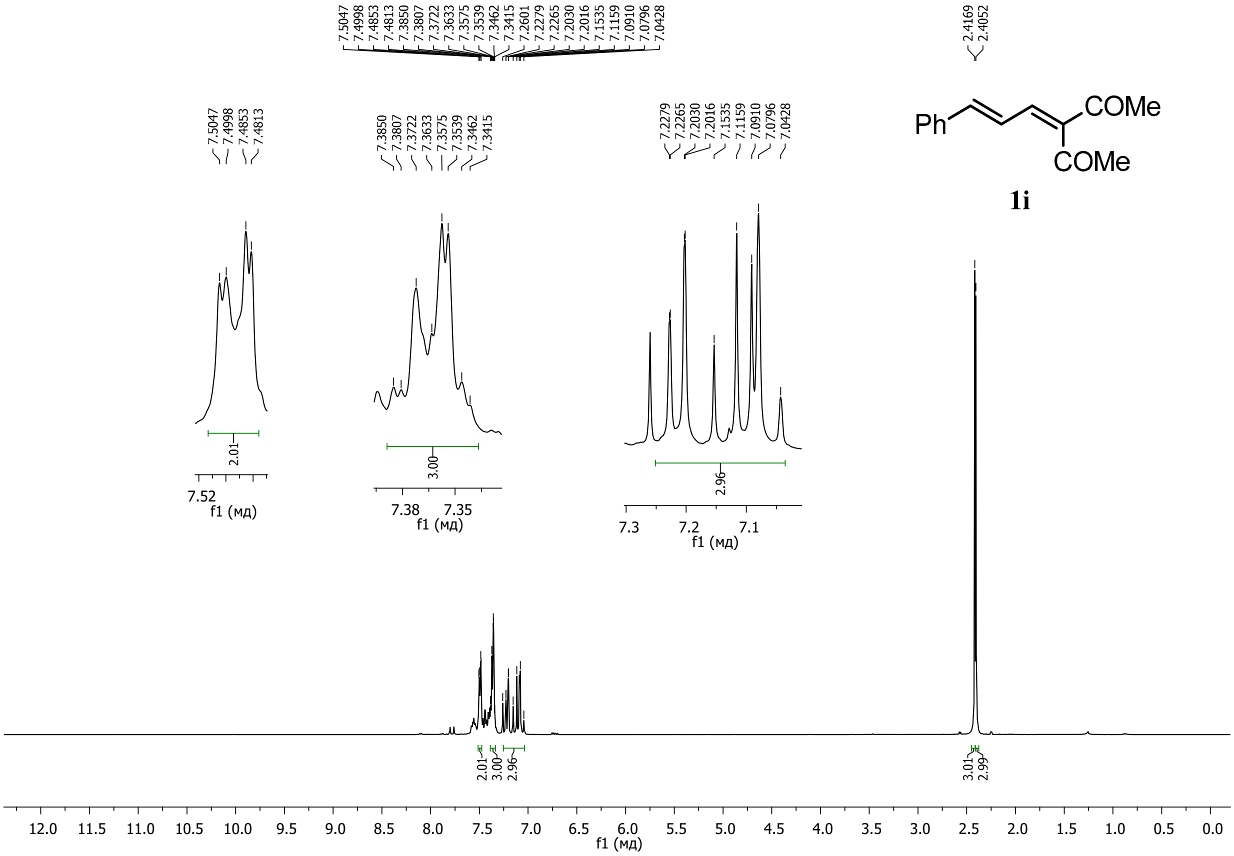

Figure S17. ^{1}H NMR spectrum of compound **1i** (400 MHz, ($CDCl_3$).

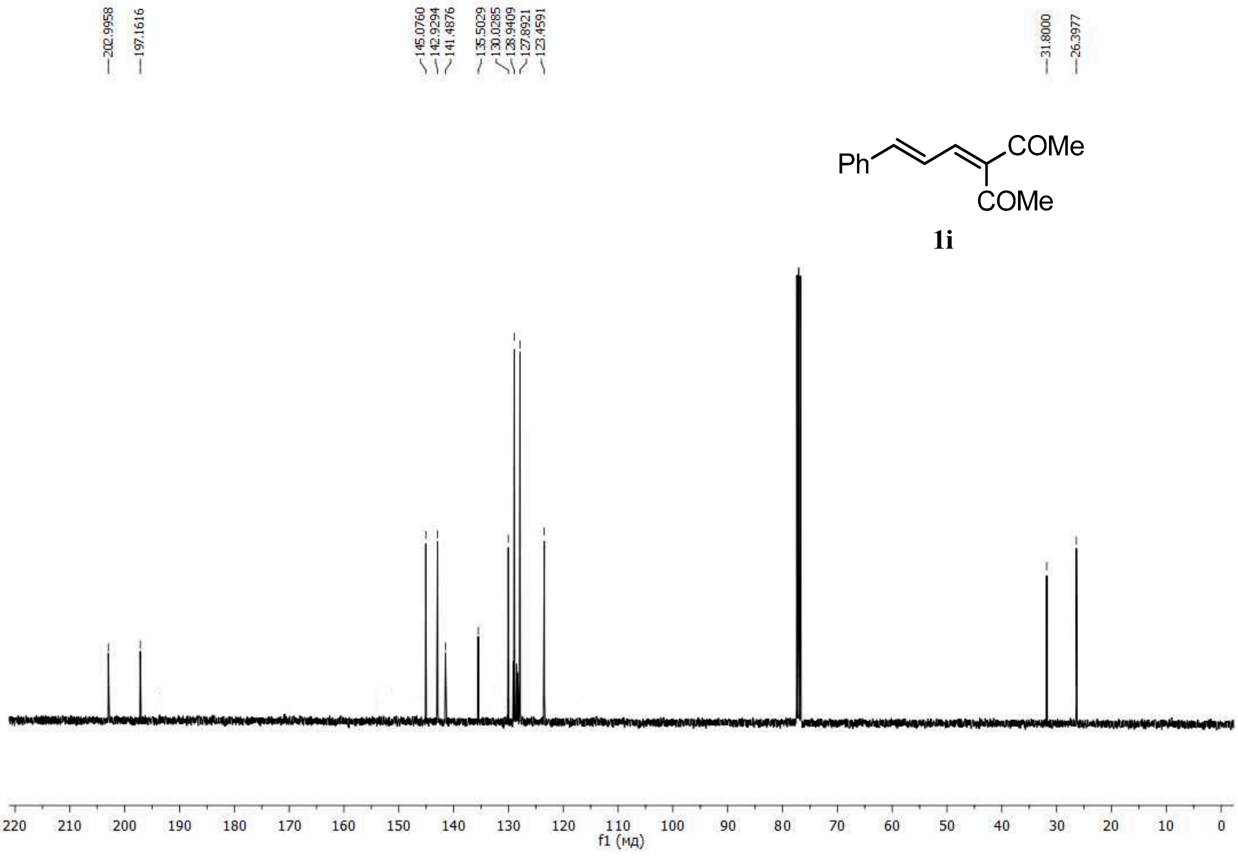

Figure S18. ^{13}C NMR spectrum of compound **1i** (100 MHz, ($CDCl_3$).

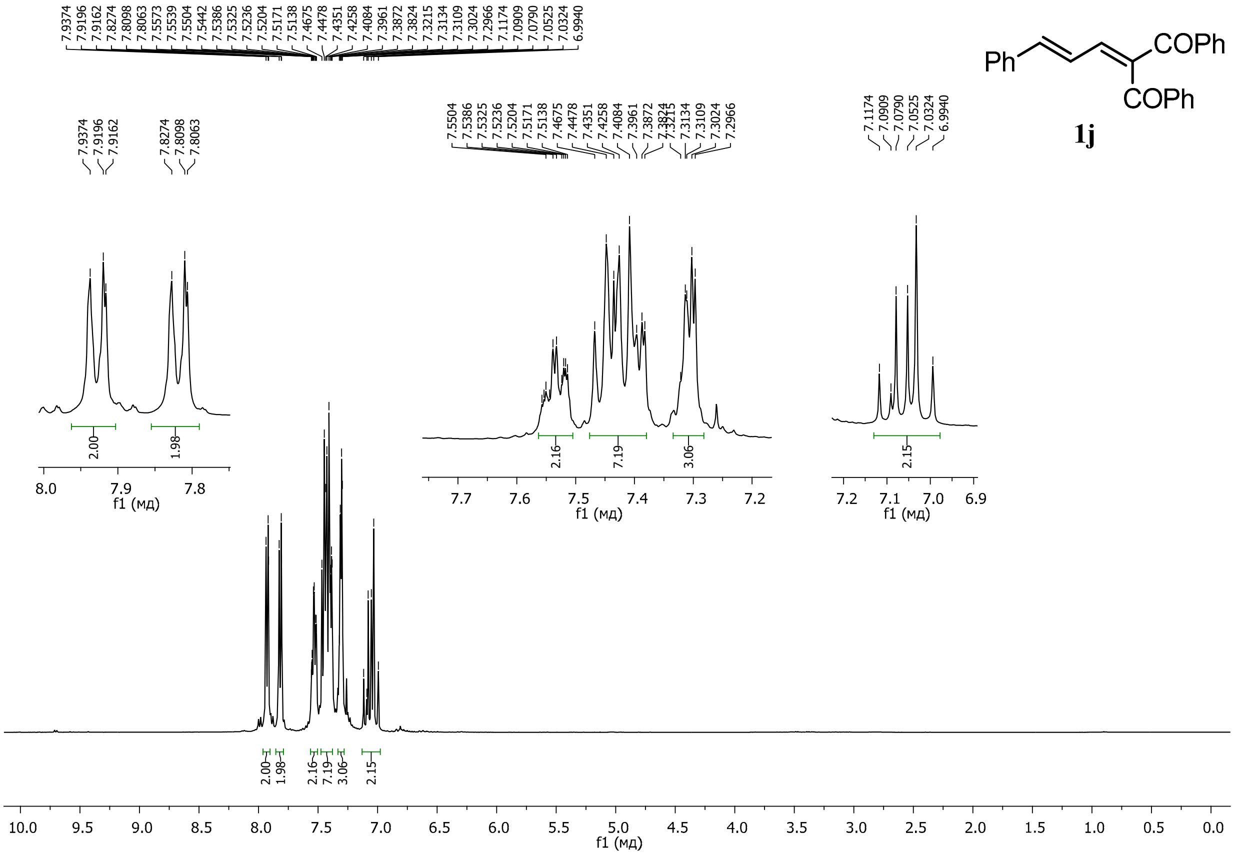

Figure S19. ^{1}H NMR spectrum of compound **1j** (400 MHz, $CDCl_3$).

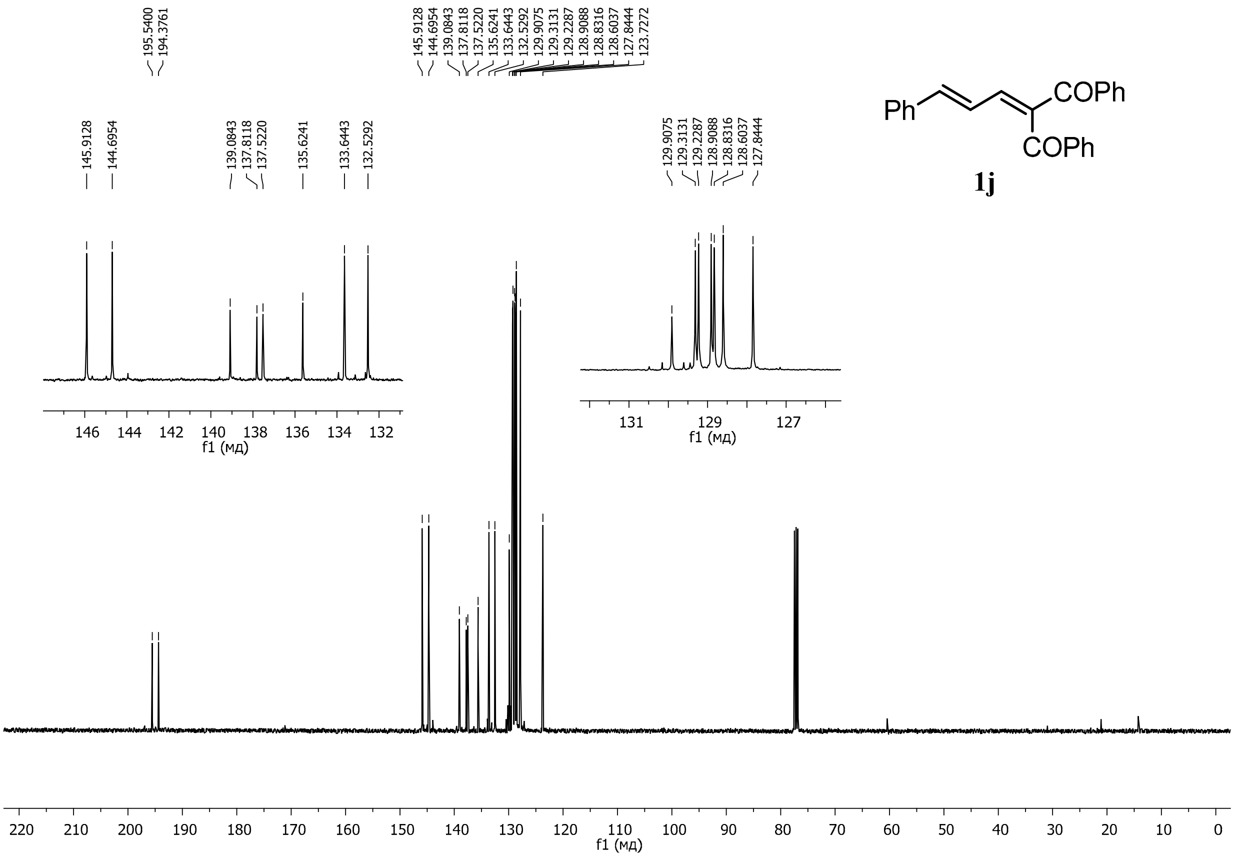

Figure S20. ^{13}C NMR spectrum of compound **1j** (100 MHz, $CDCl_3$).

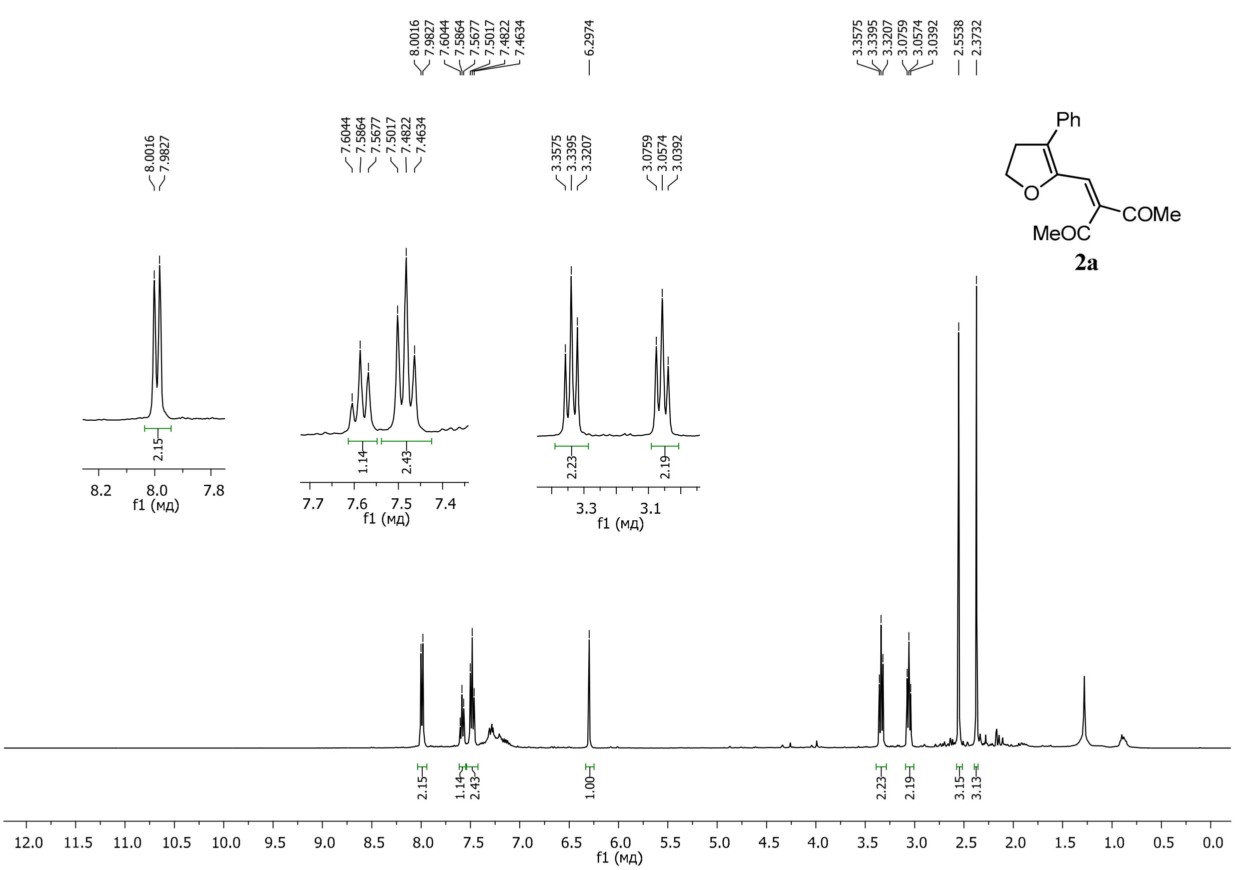

Figure S21. ^{1}H NMR spectrum of compound **2a** (400 MHz, $CDCl_3$).

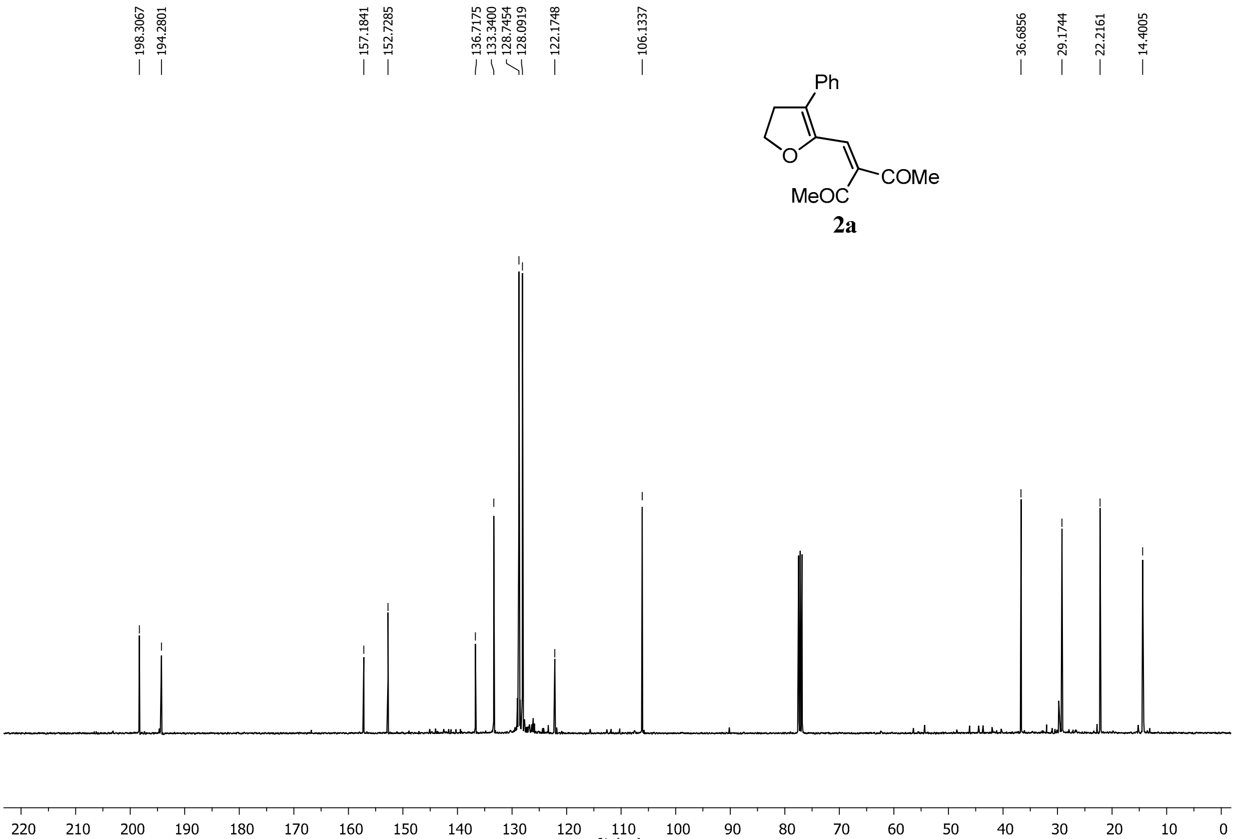

Figure S22. ^{13}C NMR spectrum of compound **2a** (100 MHz, $CDCl_3$).

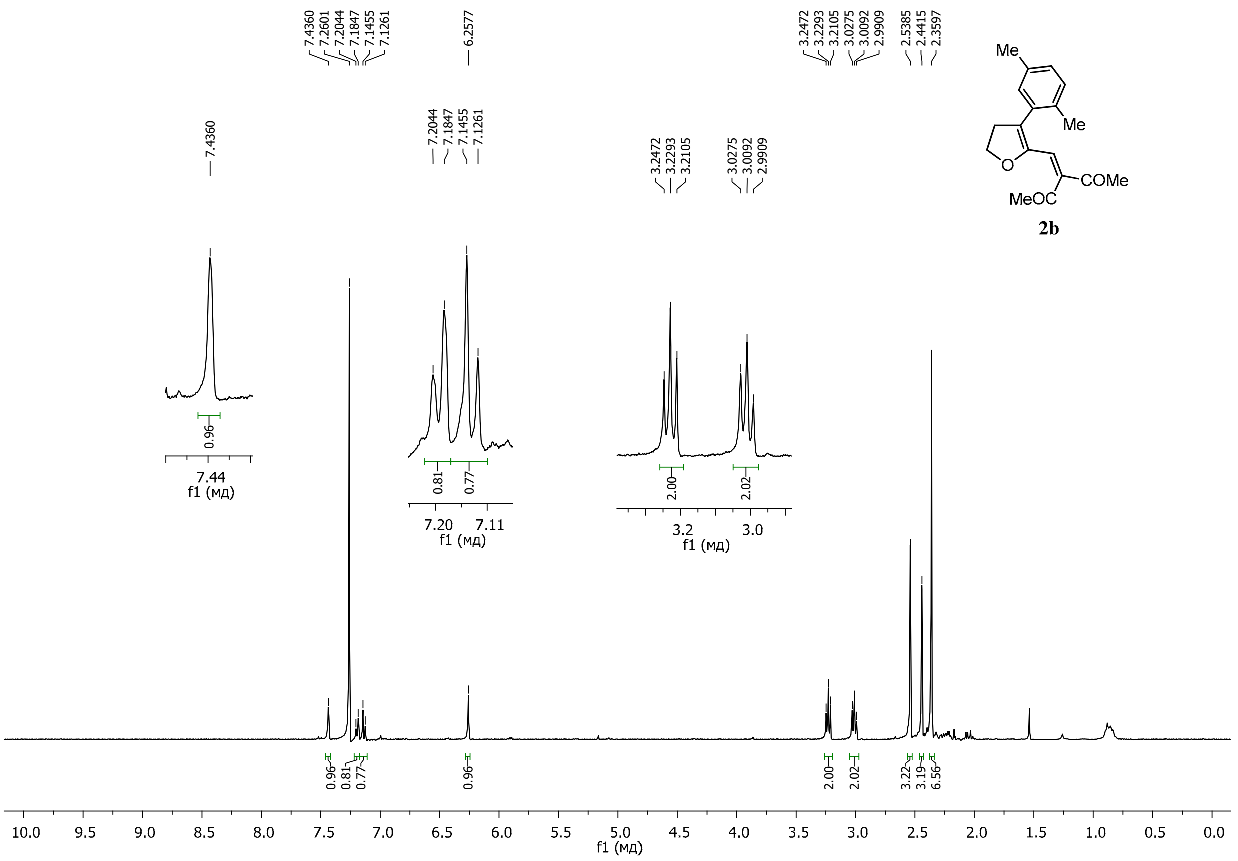

Figure S23. ^{1}H NMR spectrum of compound **2b** (400 MHz, $CDCl_3$).

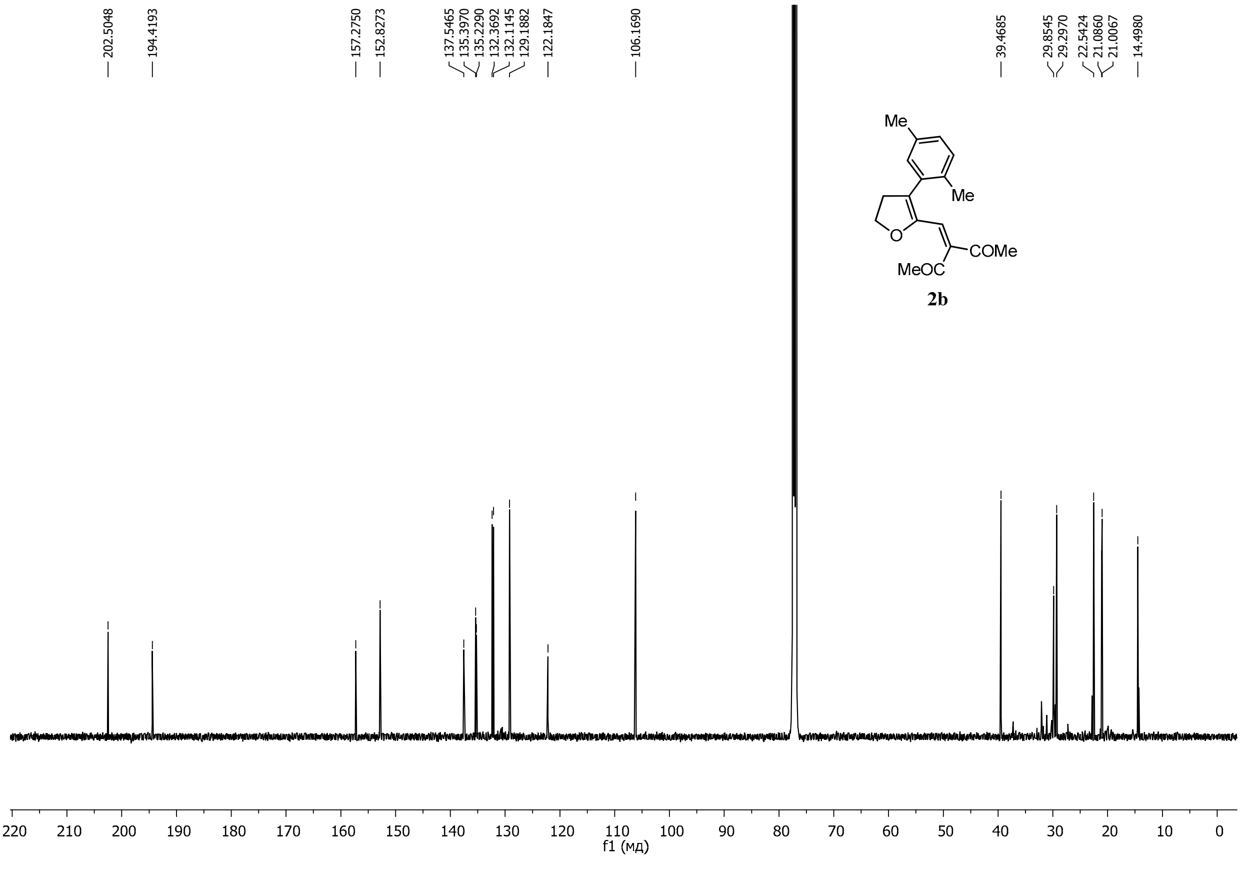

Figure S24. ^{13}C NMR spectrum of compound **2b** (100 MHz, $CDCl_3$).

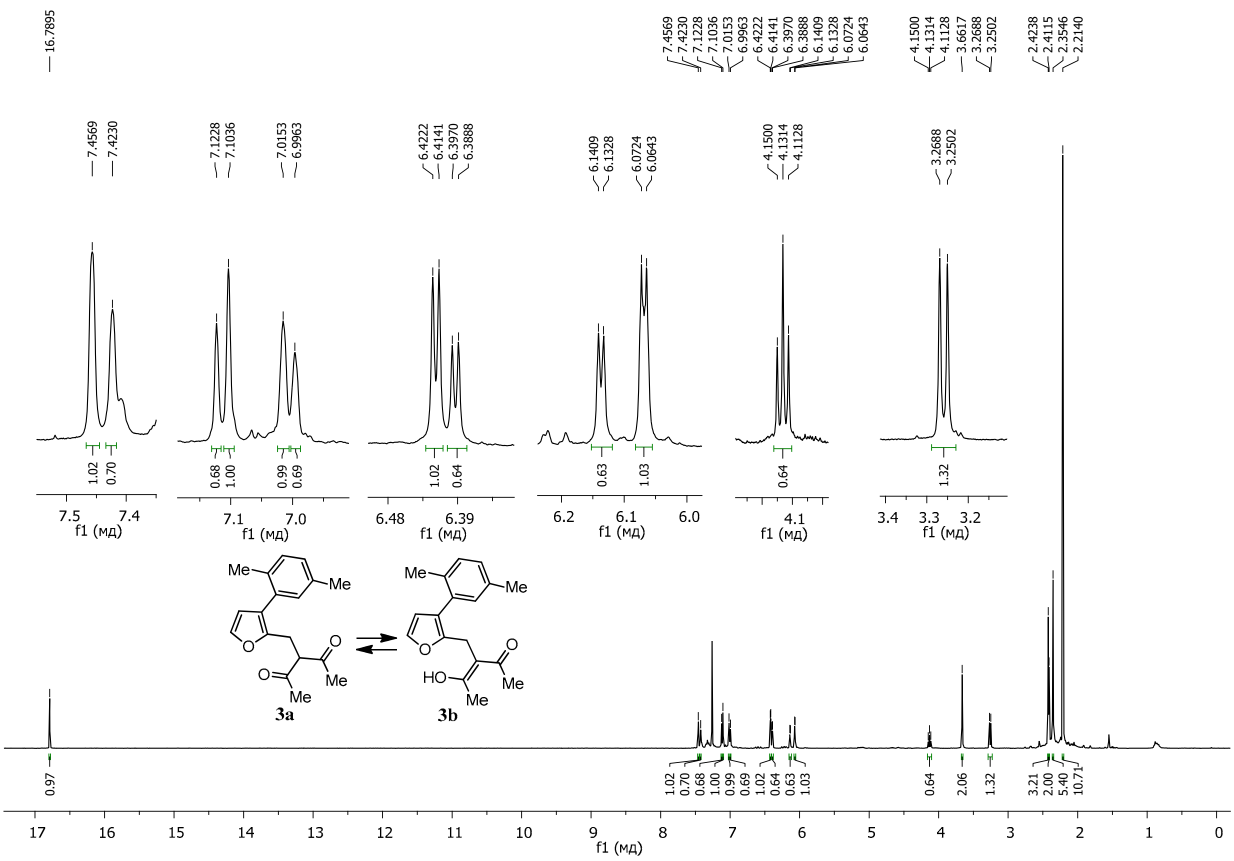

Figure S25. ^{1}H NMR spectrum of compounds**3a** and**3b** (400 MHz, $CDCl_3$).

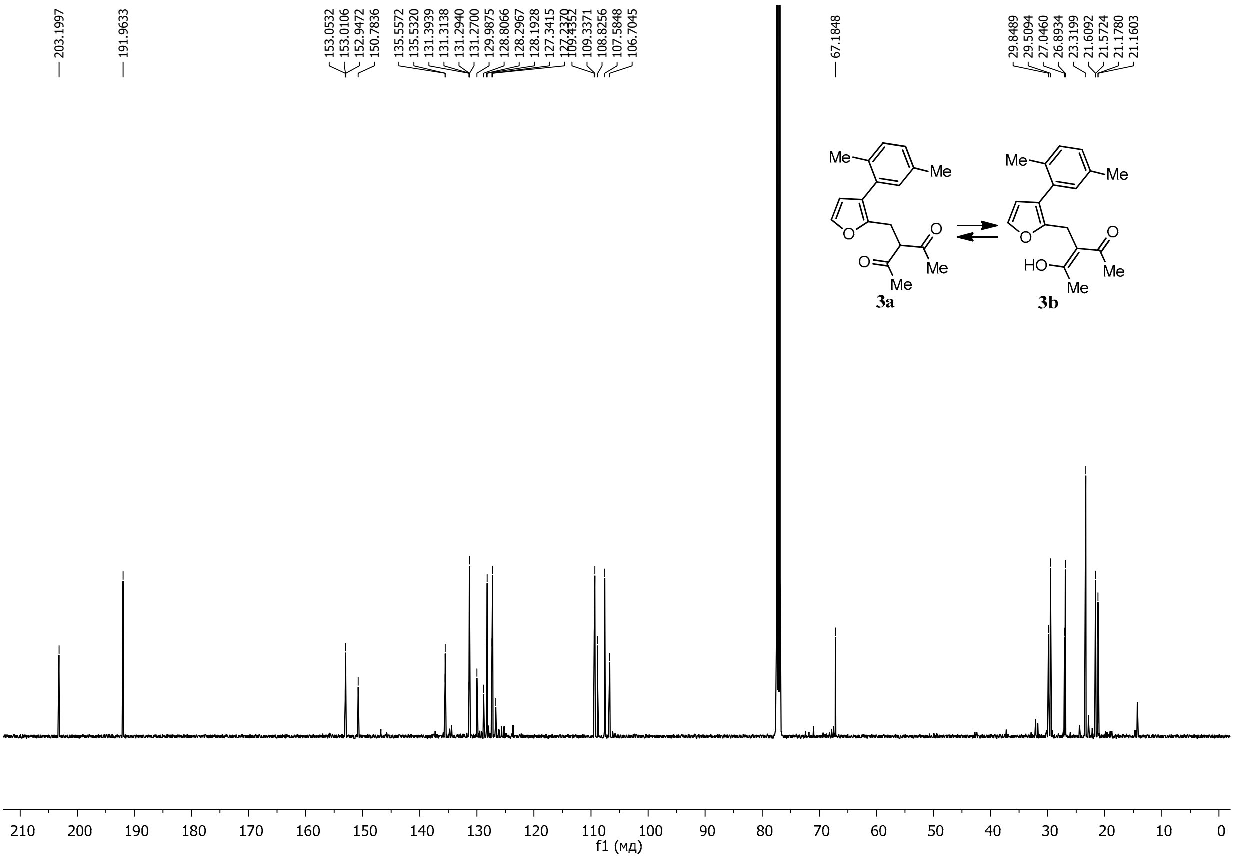

Figure S26. ^{13}C NMR spectrum of compounds **3a** and **3b** (100 MHz, $CDCl_3$).

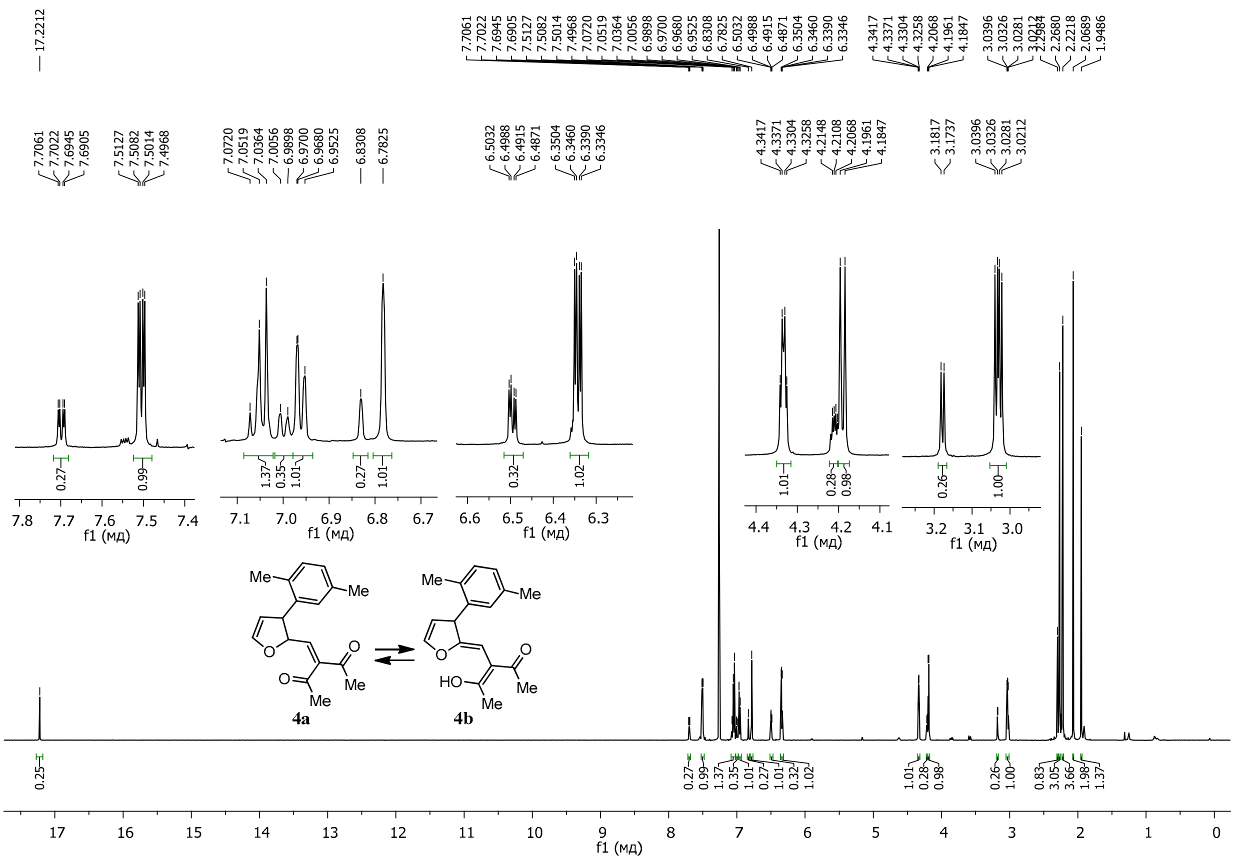

Figure S27. ^{1}H NMR spectrum of compounds **4a** and **4b** (400 MHz, $CDCl_3$).

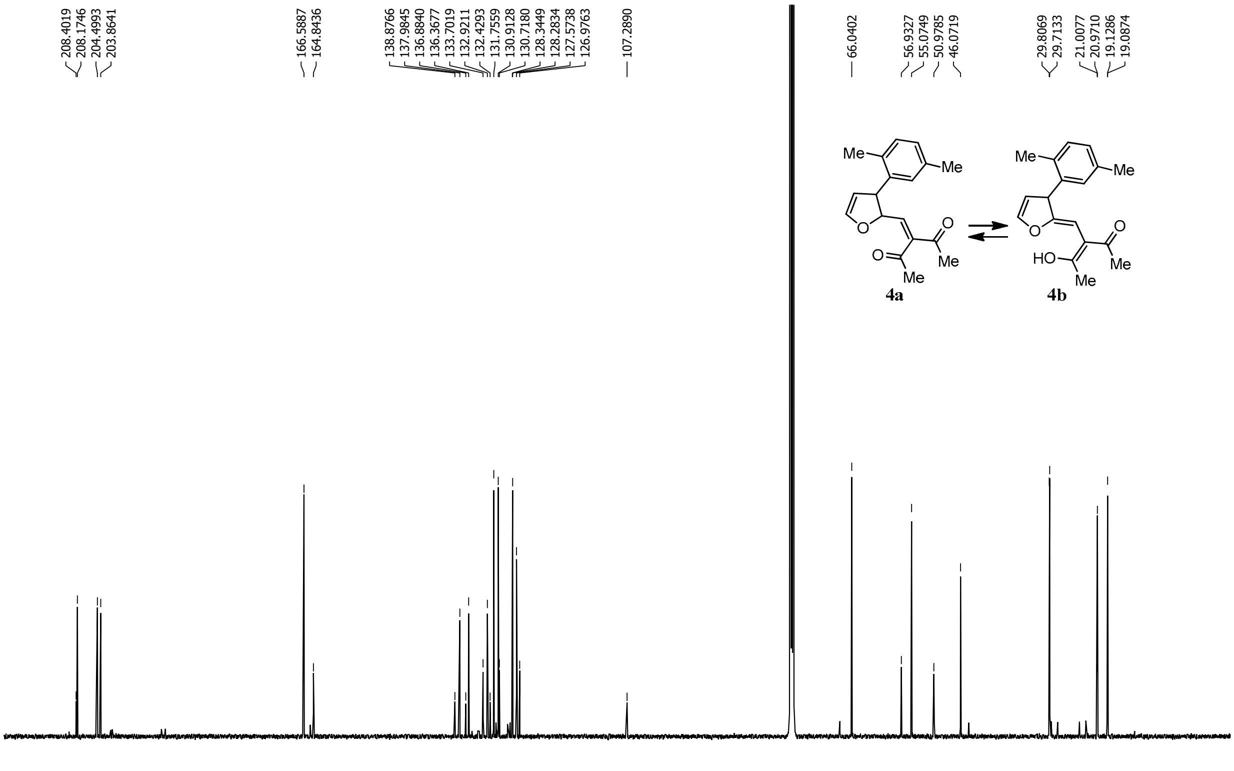

Figure S28. ^{13}C NMR spectrum of compounds **4a** and **4b** (100 MHz, $CDCl_3$).

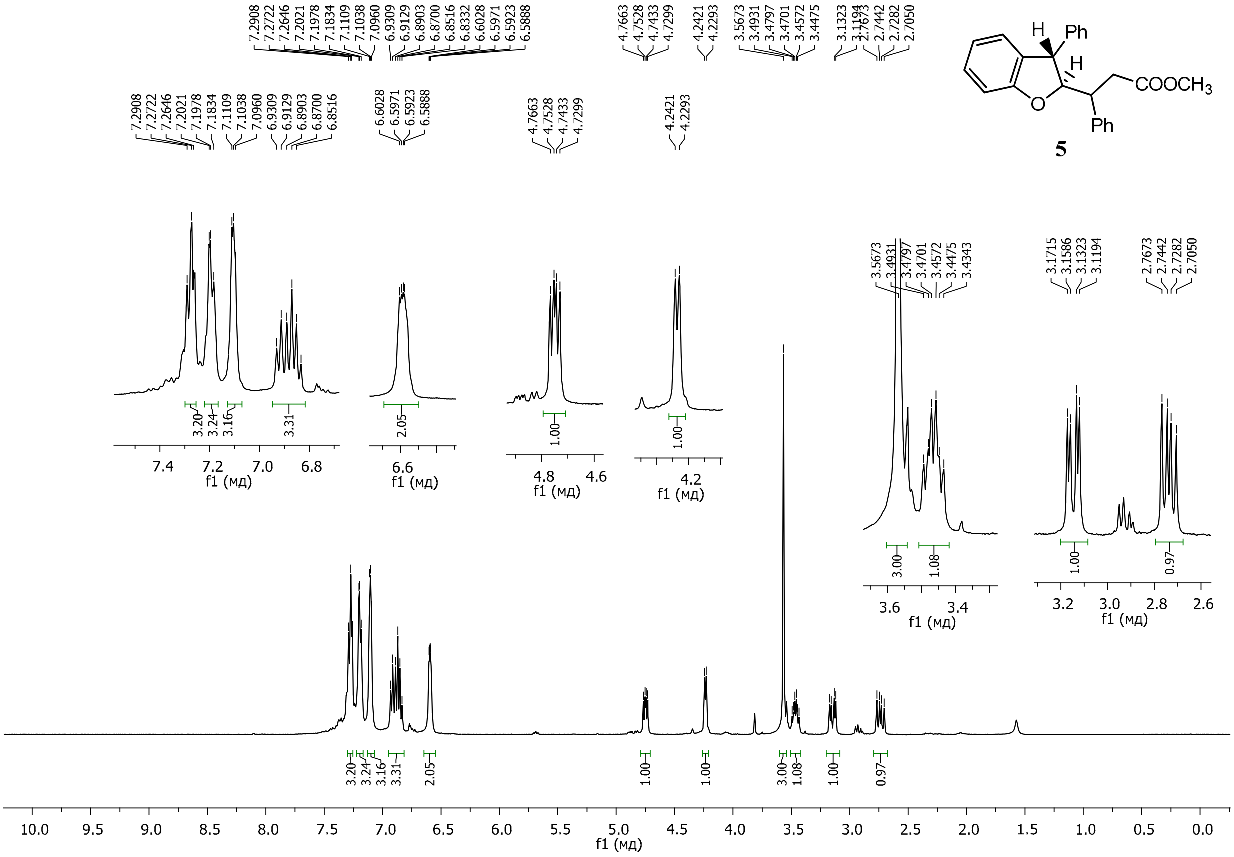

Figure S29. ^{1}H NMR spectrum of compound **5** (400 MHz, $CDCl_3$).

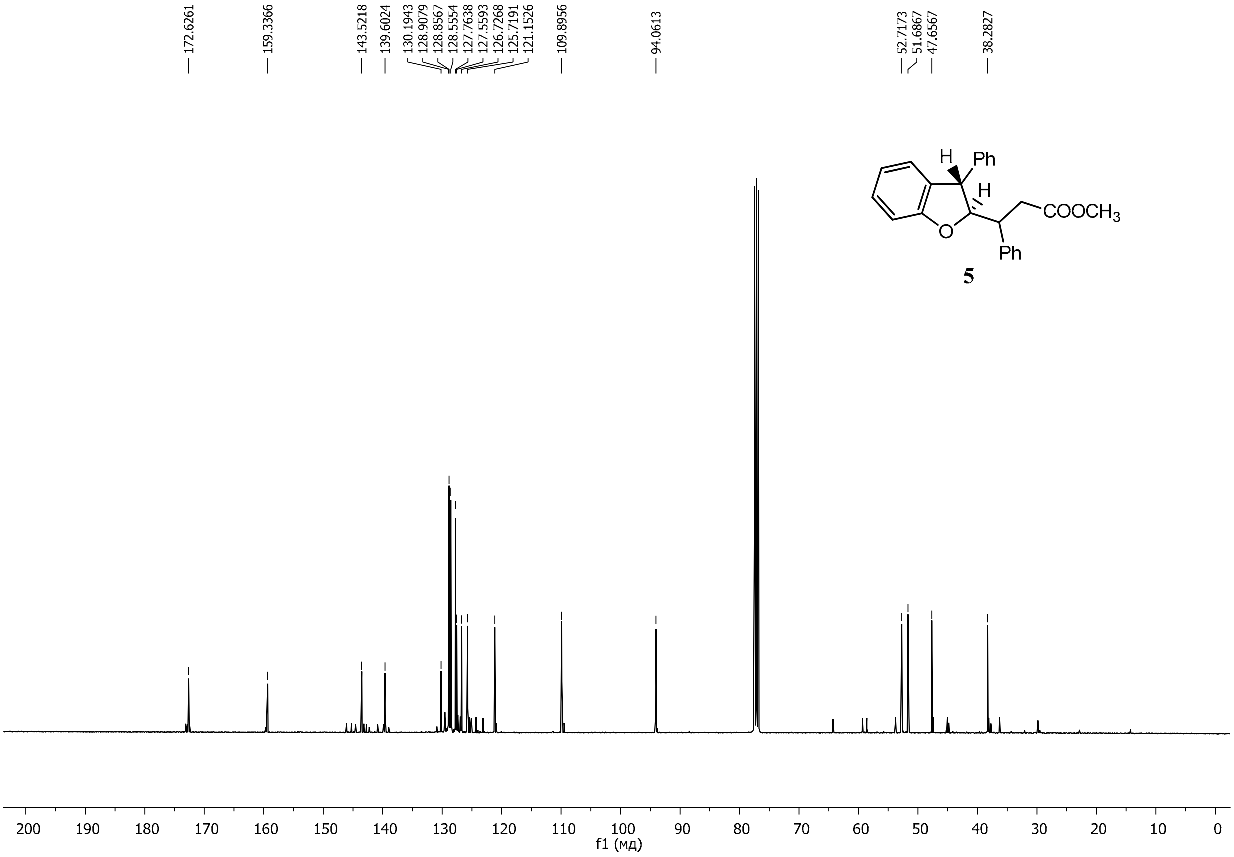

Figure S30. ^{13}C NMR spectrum of compound **5** (100 MHz, $CDCl_3$).

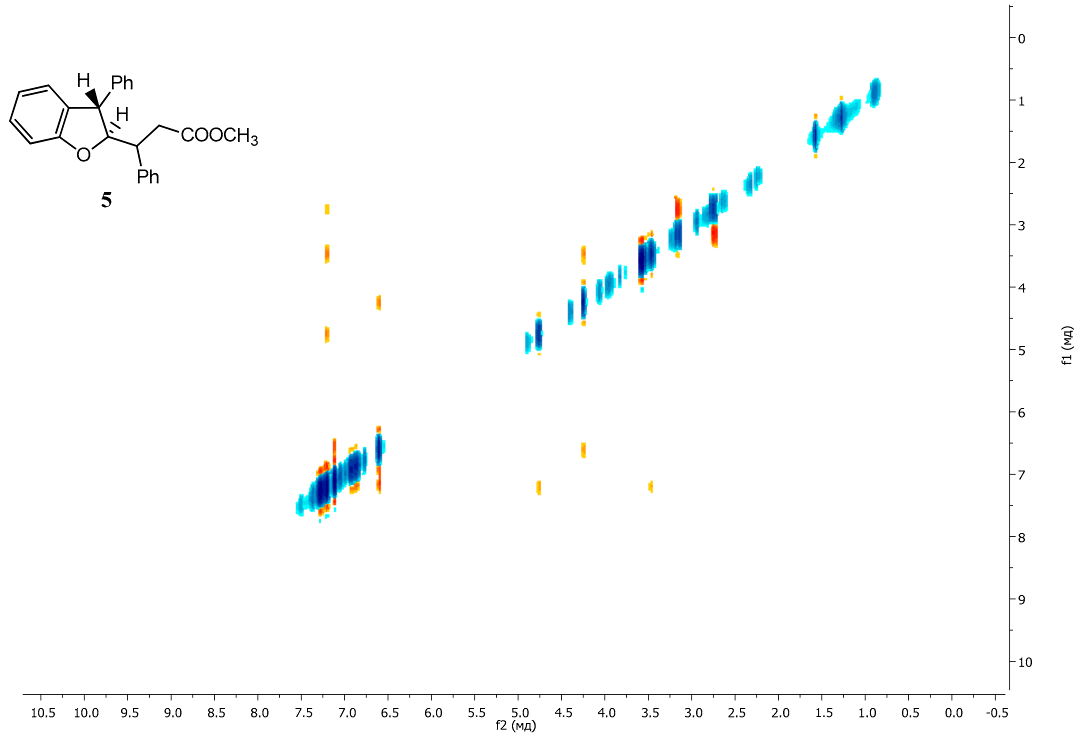

Figure S31. ^{1}H–^{1}H NOESY spectrum of compound **5** (400 MHz, CDCl$_3$).

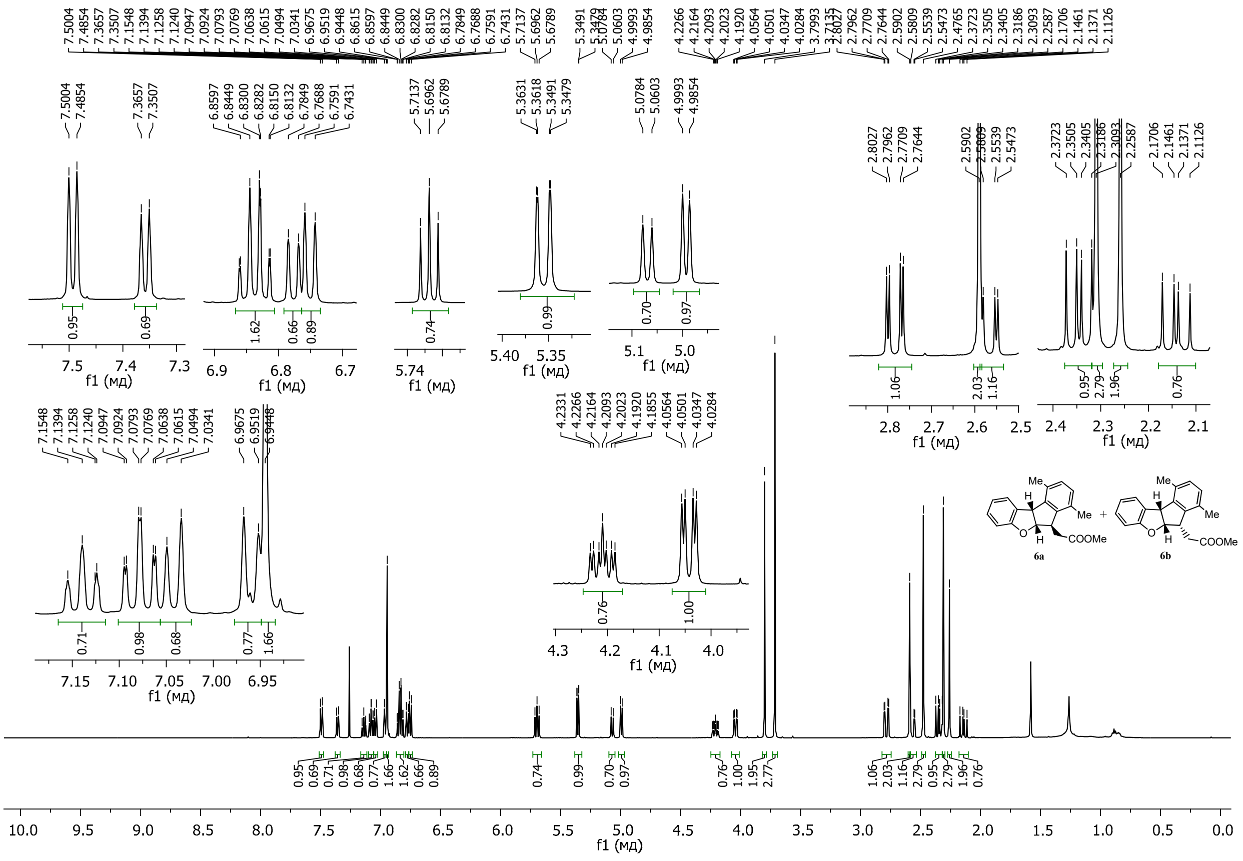

Figure S32. ^{1}H NMR spectrum of compounds **6a** and **6b** (400 MHz, (CDCl$_3$).

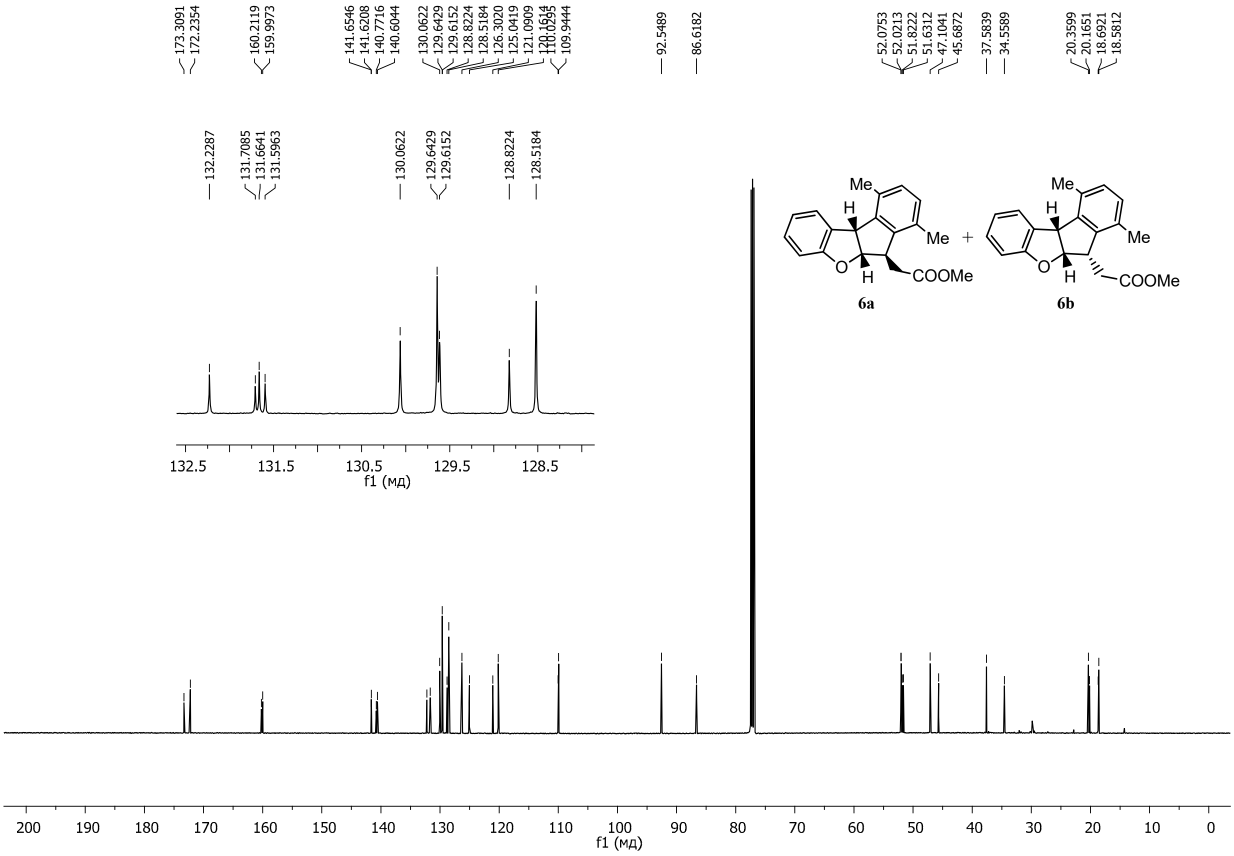

Figure S33. ^{13}C NMR spectrum of compounds **6a** and **6b** (100 MHz, $CDCl_3$).

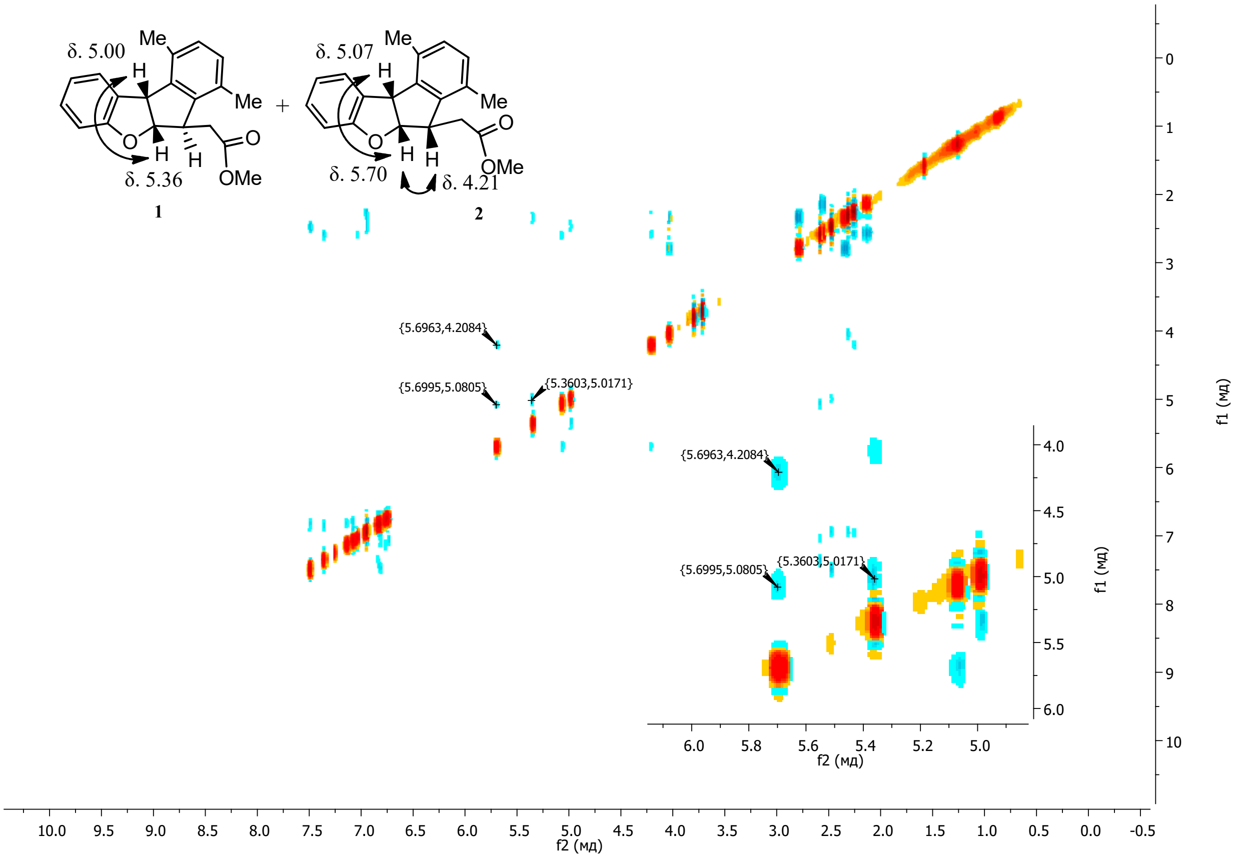

Figure S34. NOESY NMR spectrum of compound **6a** and **6b** (400 MHz, $CDCl_3$).

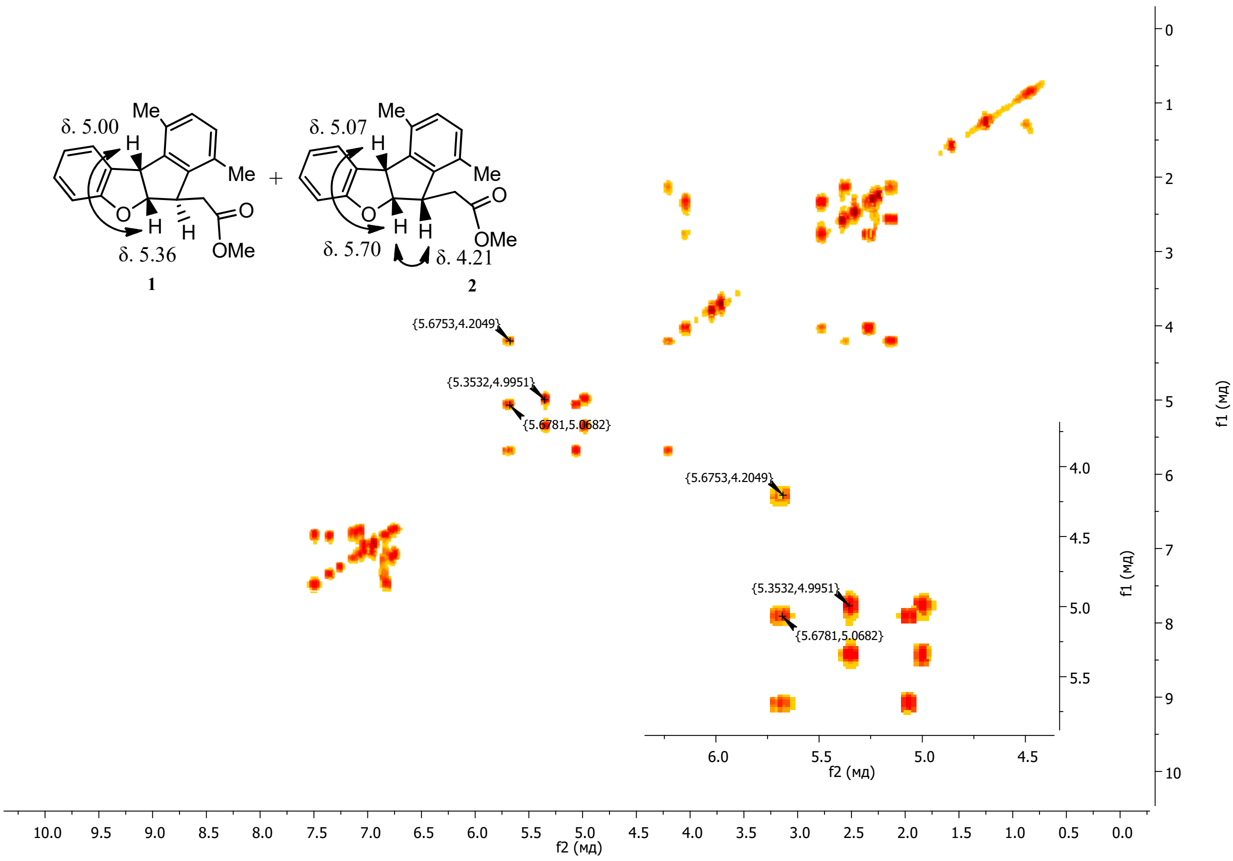

Figure S35. H-H COSY NMR spectrum of compound **6a** and **6b** (400 MHz, $CDCl_3$).

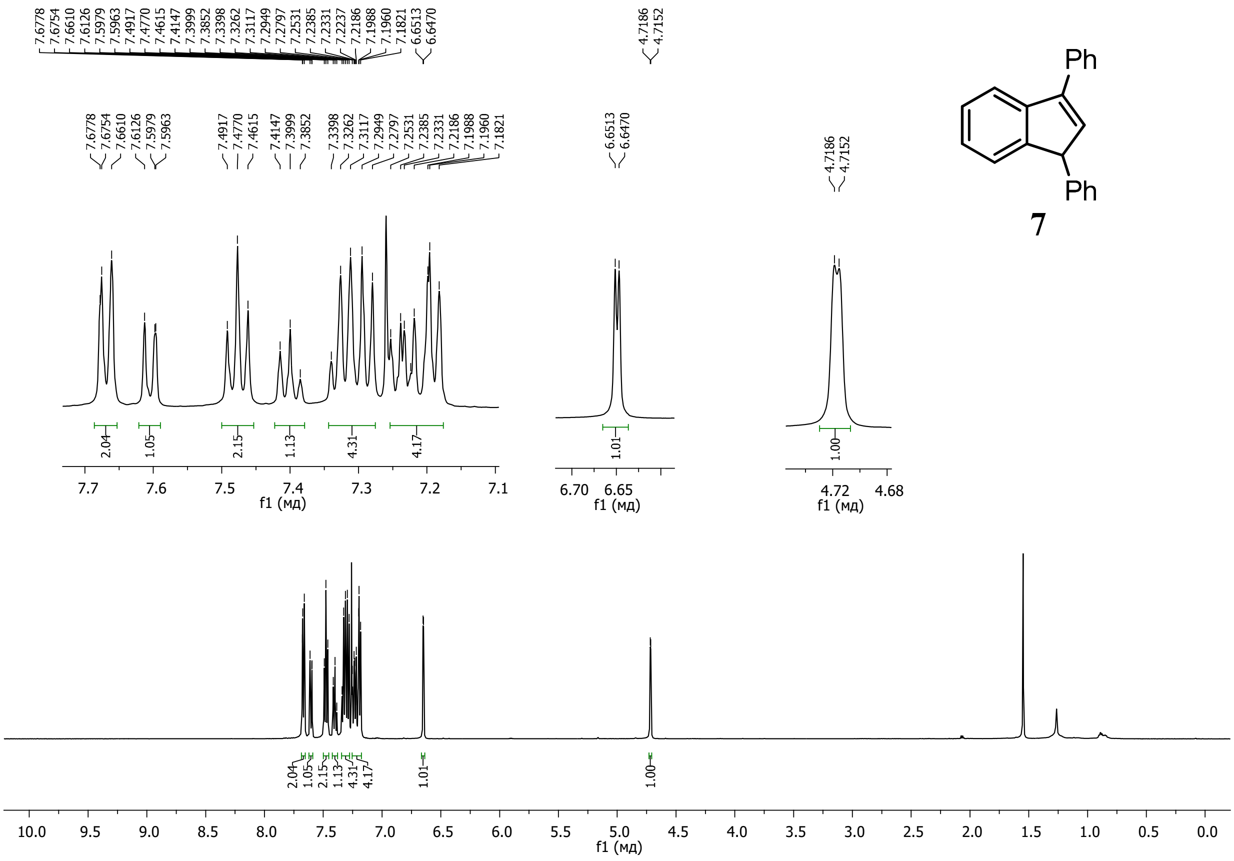

Figure S36. ^{1}H NMR spectrum of compound **7** (500 MHz, $CDCl_3$).

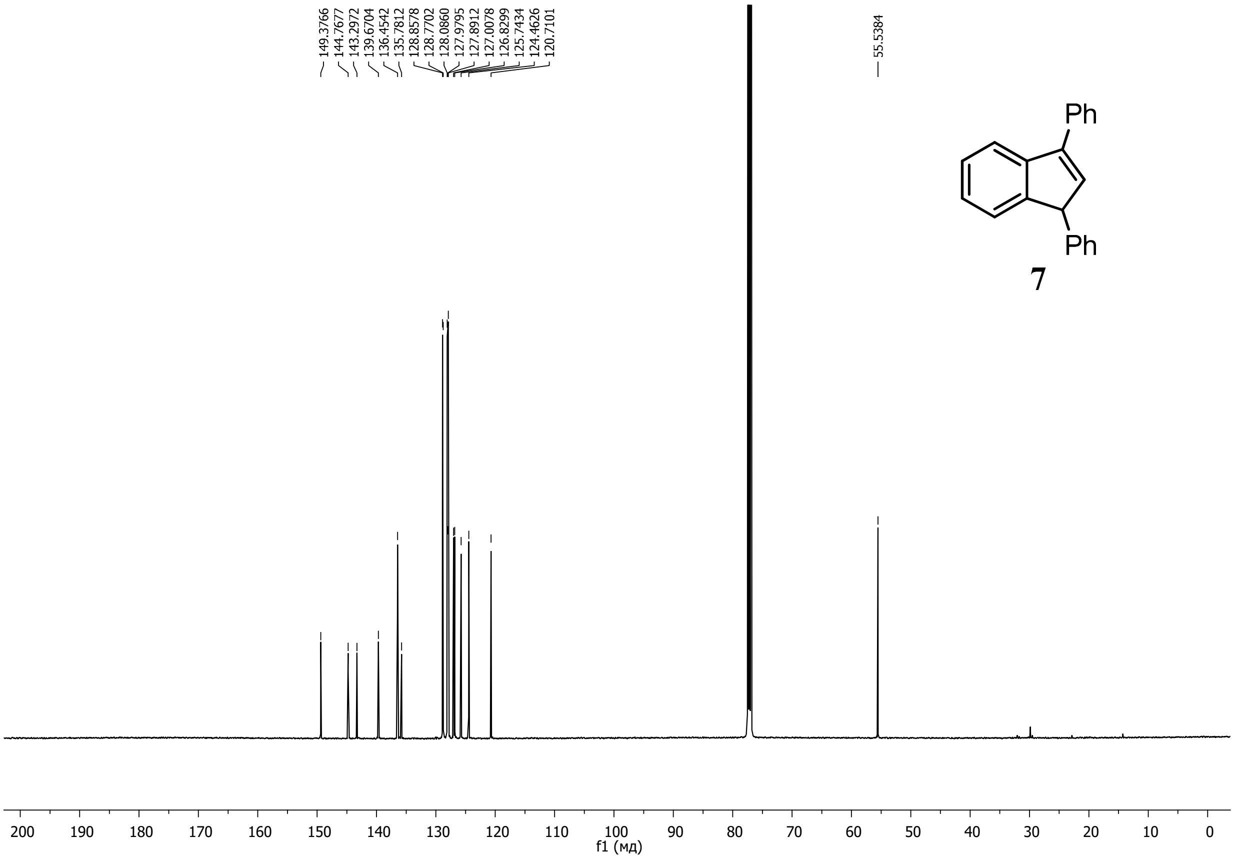

Figure S37. ^{13}C NMR spectrum of compound **7** (125 MHz, CDCl$_3$).

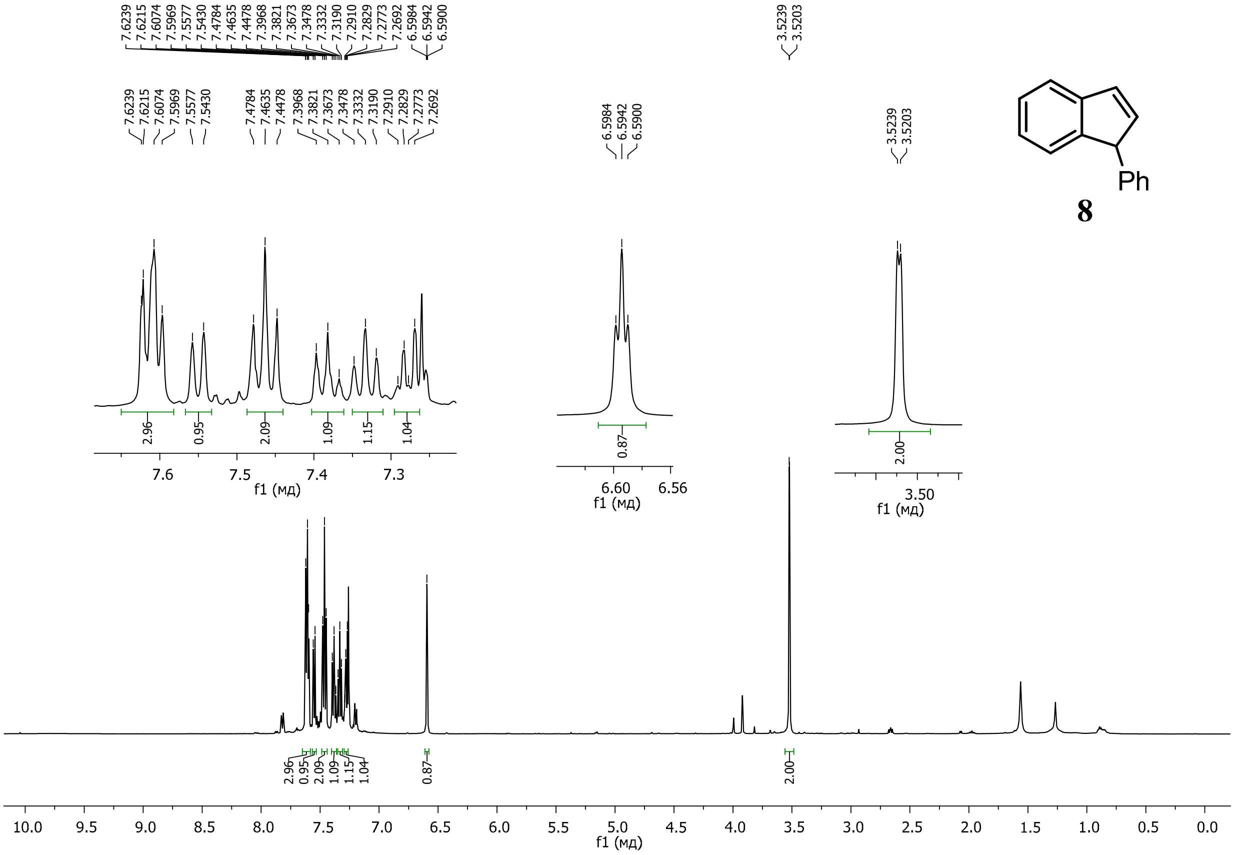

Figure S38. ^{1}H NMR spectrum of compound **8** (400 MHz, CDCl$_3$).

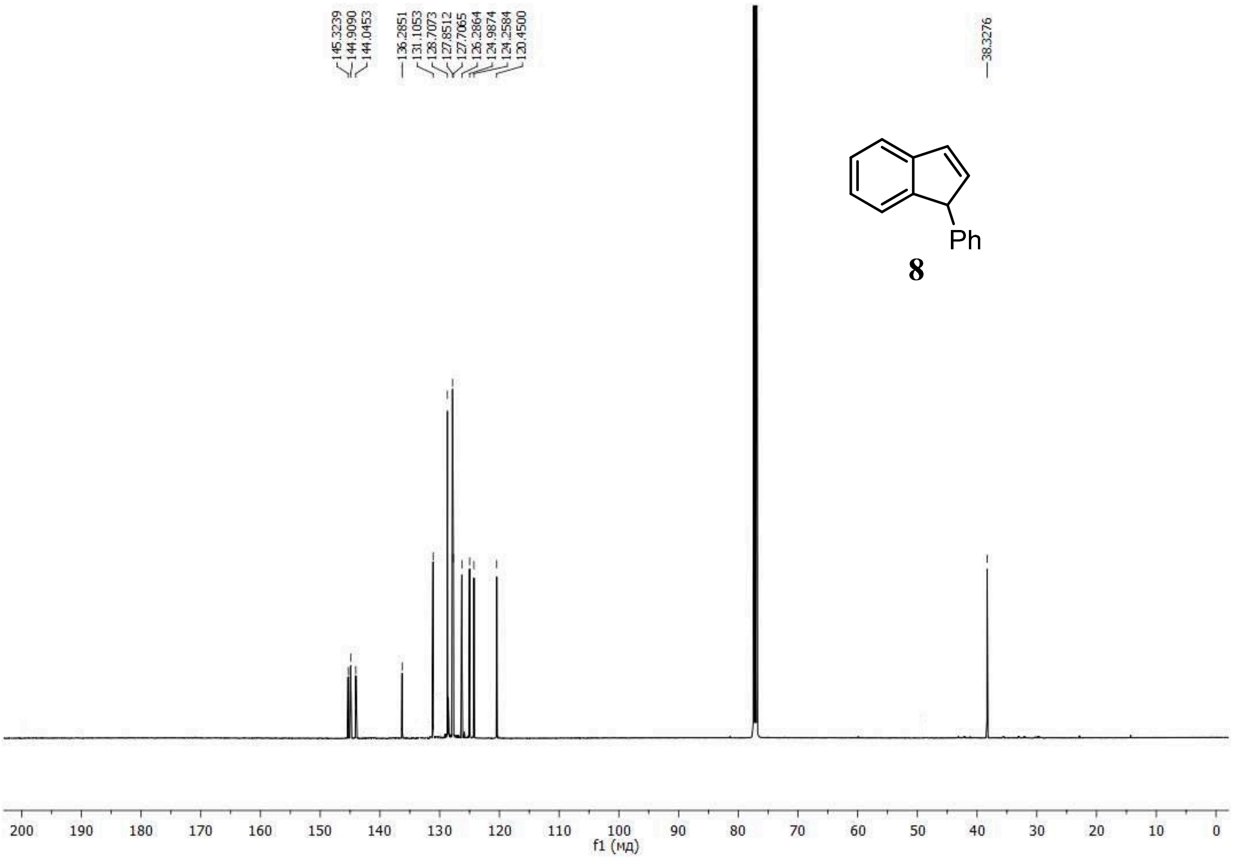

Figure S39. ^{13}C NMR spectrum of compound **8** (100 MHz, $CDCl_3$).

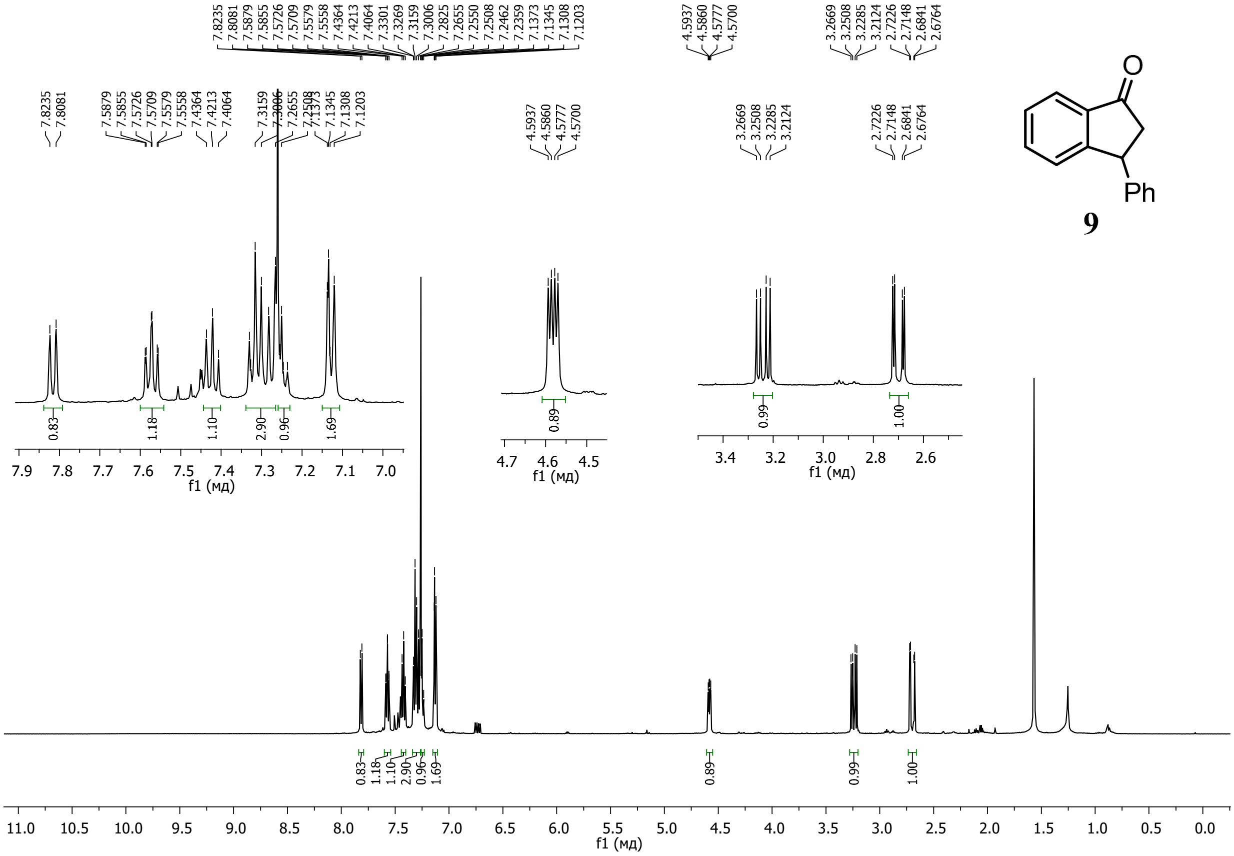

Figure S40. ^{1}H NMR spectrum of compound **9** (400 MHz, $CDCl_3$).

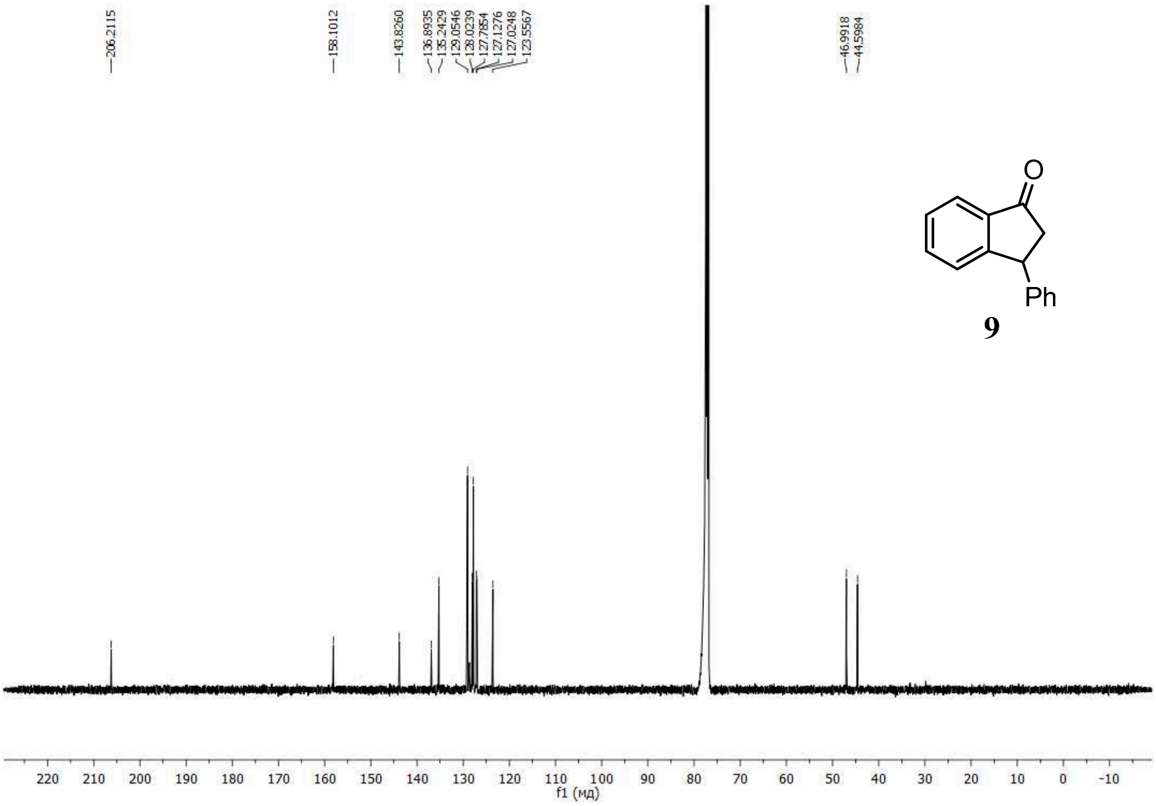

9

Figure S41. ^{13}C NMR spectrum of compound **9** (100MHz, $CDCl_3$).

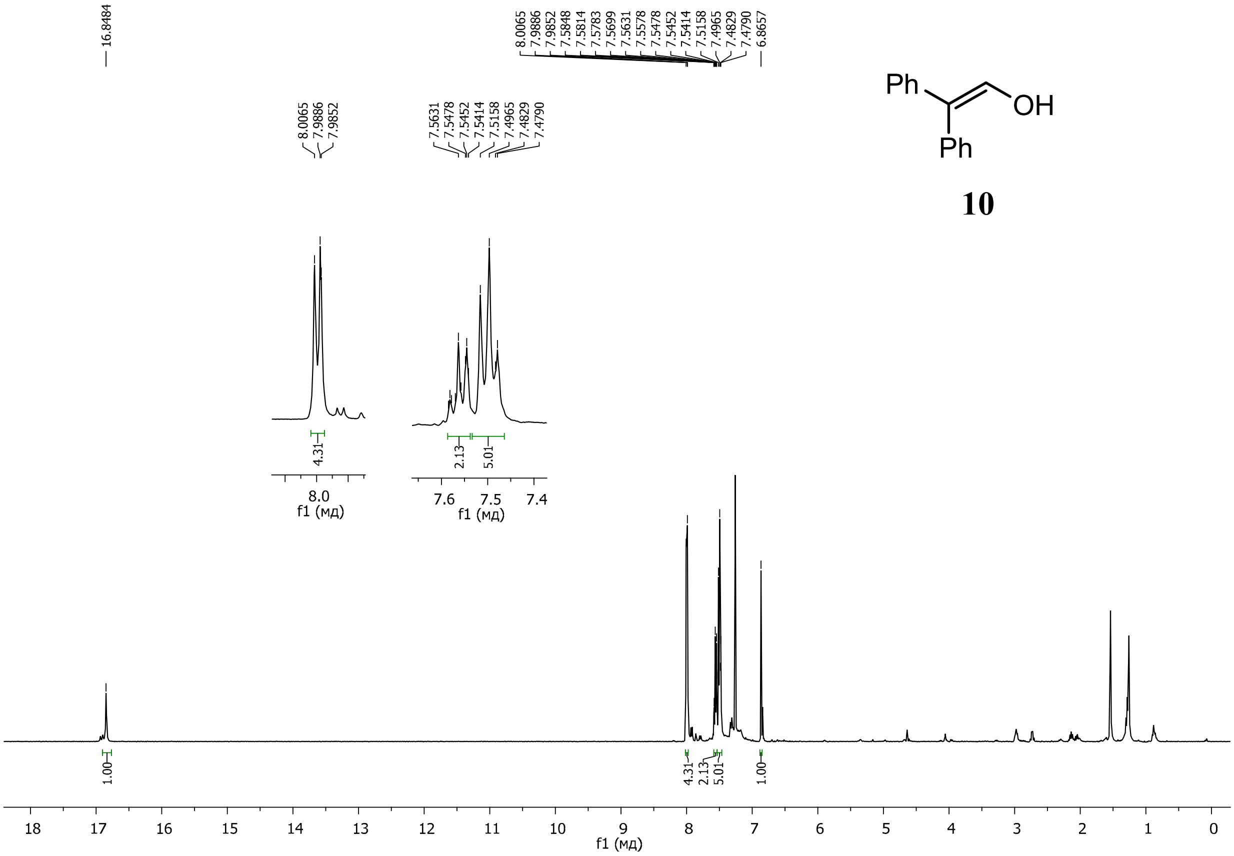

10

Figure S42. ^{1}H NMR spectrum of compound **10** (400 MHz, ($CDCl_3$).

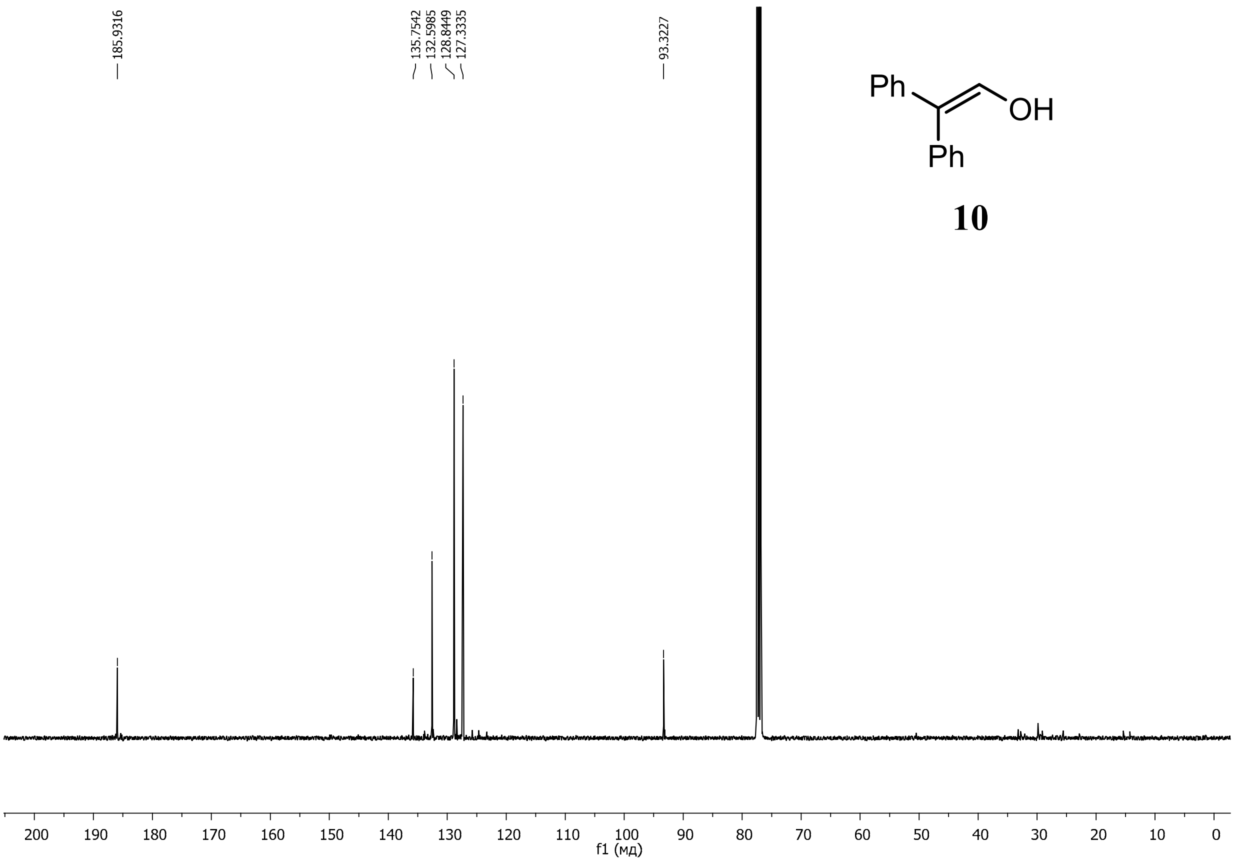

Figure S43. ^{13}C NMR spectrum of compound **10** (100 MHz, $CDCl_3$).

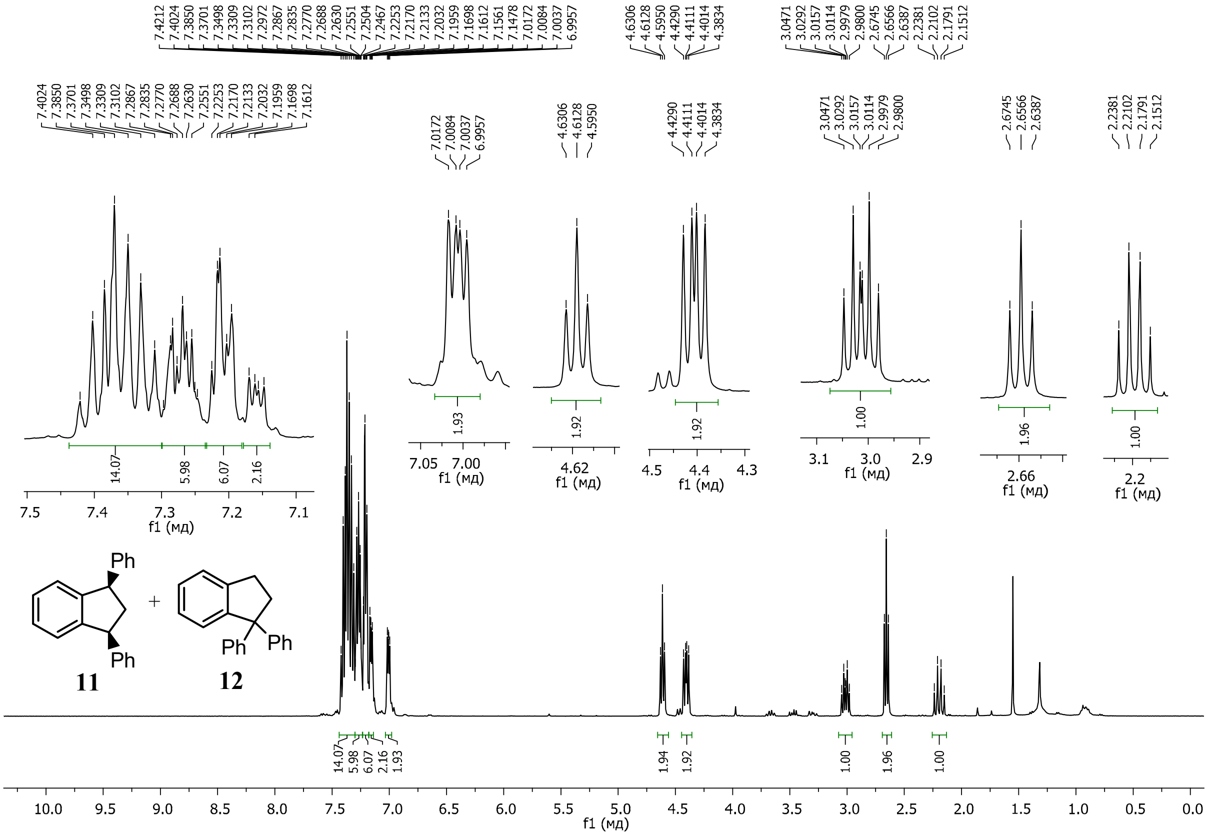

Figure S44. ^{1}H NMR spectrum of compounds **11** and **12** (400 MHz, ($CDCl_3$).

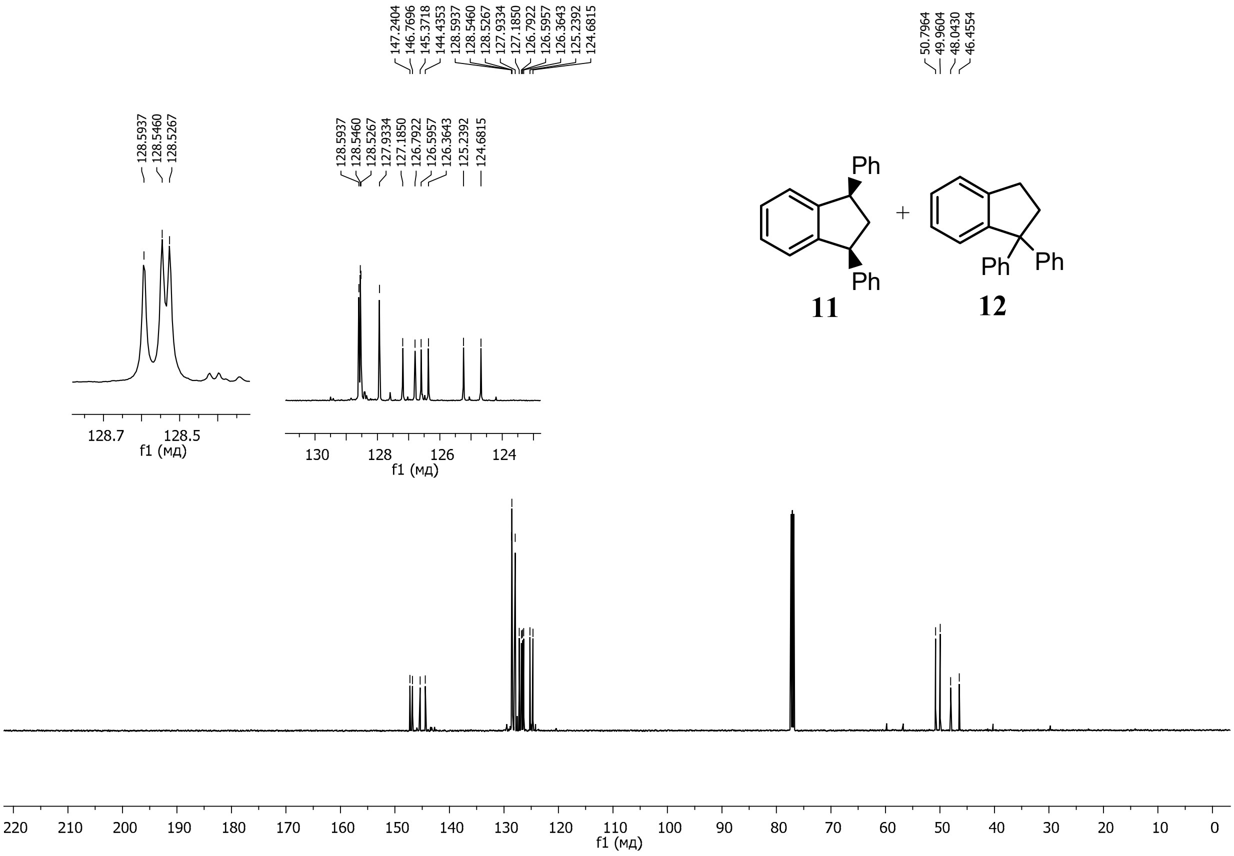

Figure S45. ^{13}C NMR spectrum of compounds **11** and **12** (100 MHz, $CDCl_3$).

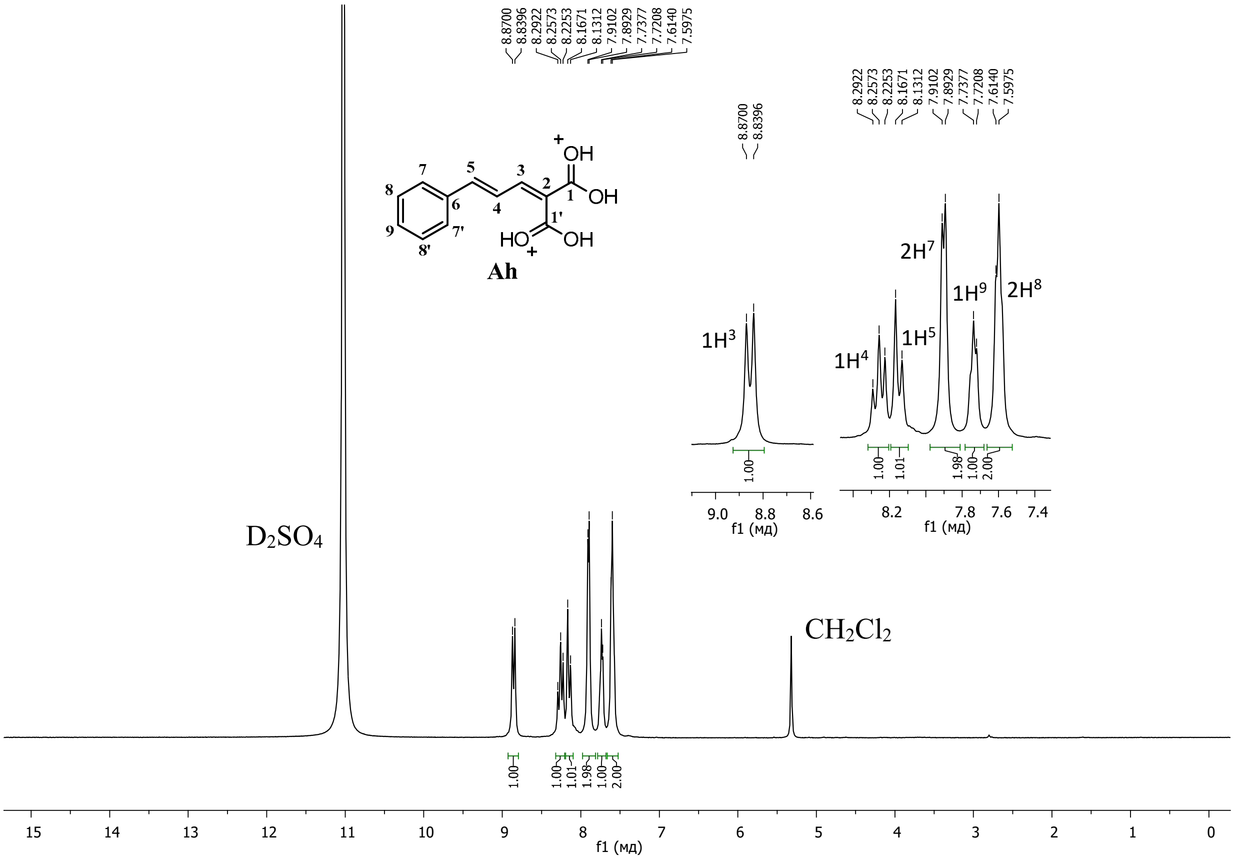

Figure S46. ^{1}H NMR spectrum of cation **Ah** in D_2SO_4 at room temperature (CH_2Cl_2 as internal standard, 400 MHz).

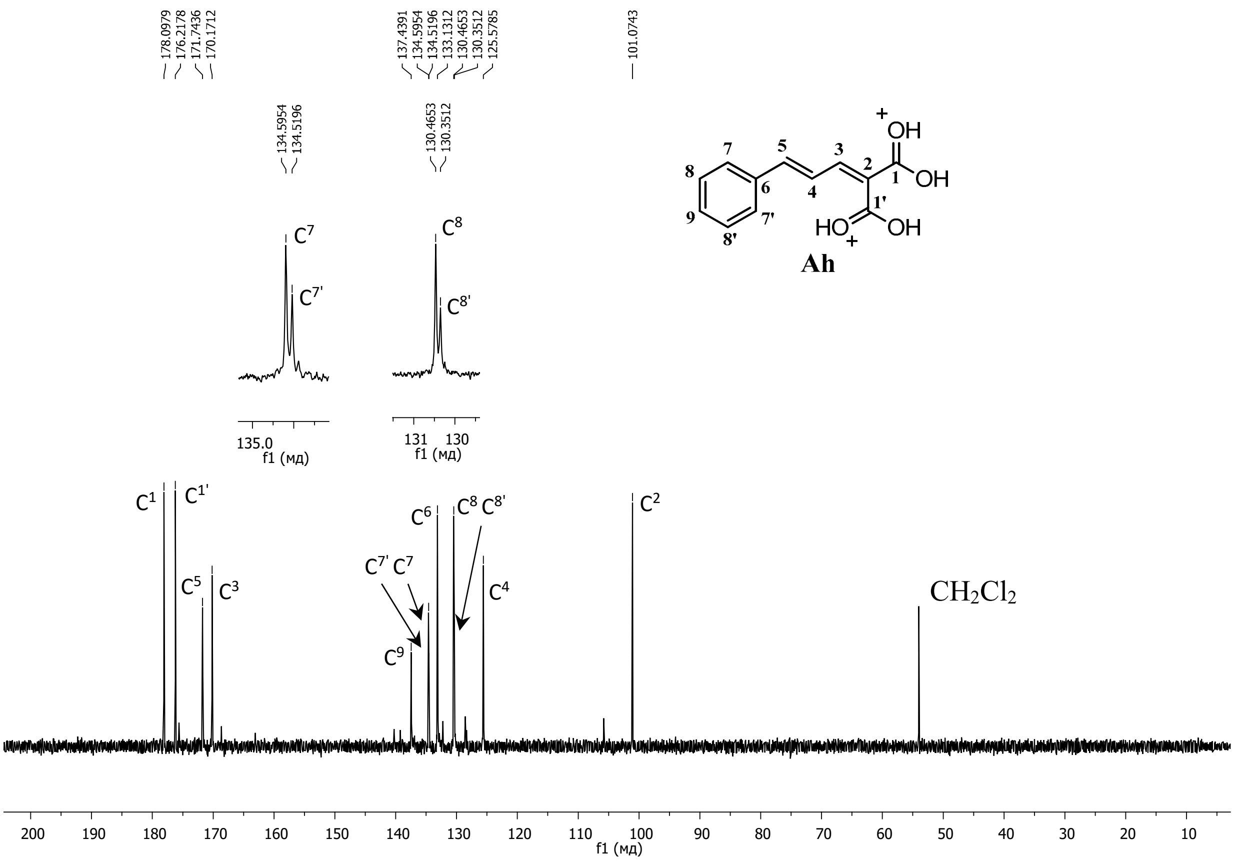

Figure S47.^{13}C NMR spectrum of cation **Ah** in D_2SO_4 at room temperature (CH_2Cl_2 as internal standard, 400 MHz).

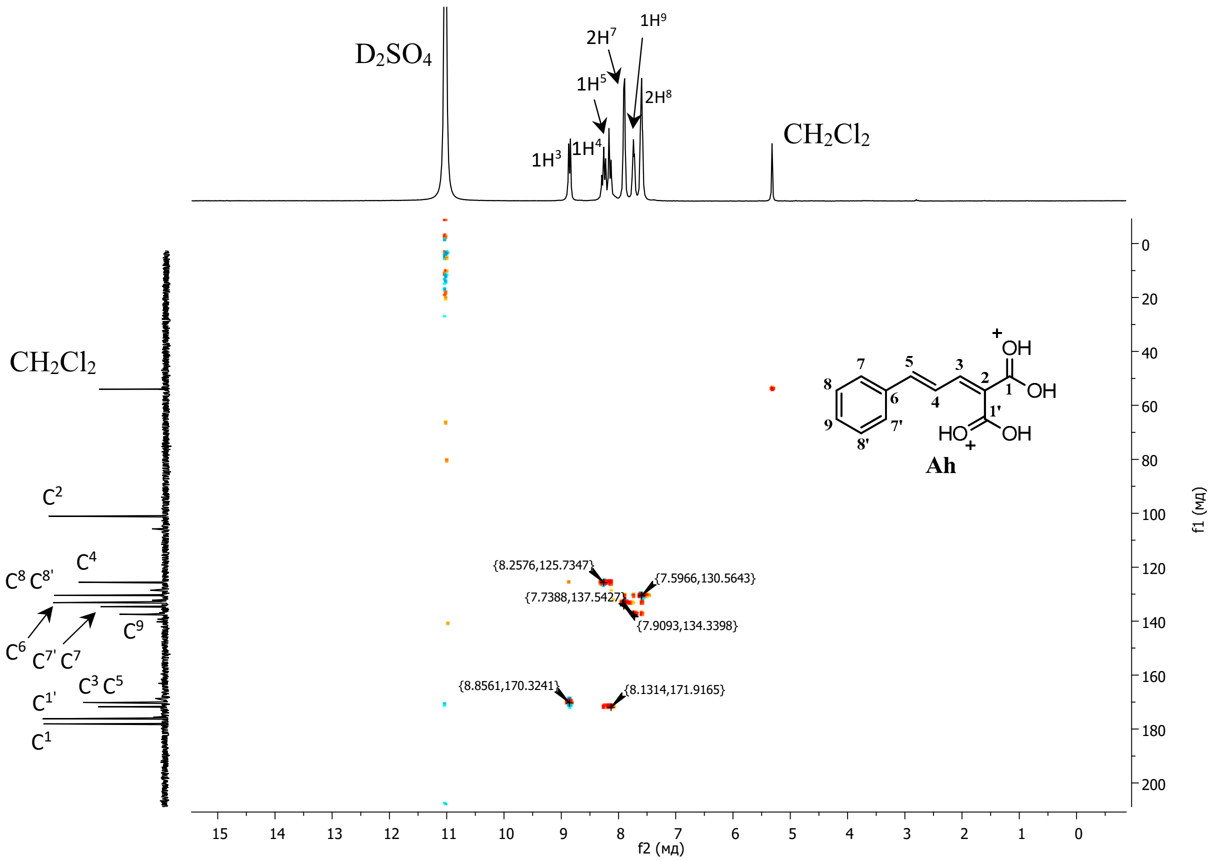

Figure S48. ^{1}H–^{13}C HSQC spectrum of cation **Ah** in D_2SO_4 at room temperature (CH_2Cl_2 as internal standard).

2. X-ray data for compound 11

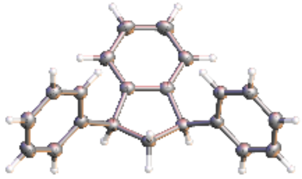

Table S1. Crystal data and structure refinement for compound 11

Empirical formula	$C_{21}H_{18}$
Formula weight	270.35
Temperature/K	100(2)
Crystal system	orthorhombic
Space group	Cmce
a/Å	20.1661(6)
b/Å	7.5589(2)
c/Å	19.3668(5)
α/°	90
β/°	90
γ/°	90
Volume/Å^3	2952.15(14)
Z	8
ρ_{calc}g/cm^3	1.217
μ/mm^{-1}	0.515
F(000)	1152.0
Crystal size/mm^3	0.17 × 0.15 × 0.13
Radiation	Cu Kα (λ = 1.54184)
2Θ range for data collection/°	8.77 to 138.156
Index ranges	$-24 \leq h \leq 23$, $-7 \leq k \leq 9$, $-22 \leq l \leq 23$
Reflections collected	4414
Independent reflections	1412 [R_{int} = 0.0206, R_{sigma} = 0.0224]
Data/restraints/parameters	1412/0/97
Goodness-of-fit on F^2	1.056
Final R indexes [I>=2σ (I)]	R_1 = 0.0477, wR_2 = 0.1291
Final R indexes [all data]	R_1 = 0.0527, wR_2 = 0.1341
Largest diff. peak/hole / e Å^{-3}	0.44/-0.18

3. Data of DFT calculations of compound 1b, and cations A-E, G, J, K, K-H$^+$, water H_2O and H_3O^+

Table S2. Selected electronic characteristics (DFT calculations) of compound **1b**, cations **A, B, C.**

Species	E_{HOMO}, eV	E_{LUMO}, eV	ω,[a] eV	$q(C^2)$,[b] e	$q(C^4)$,[b] e	$q(C^6)$,[b] e	$q(C^8)$,[b] e
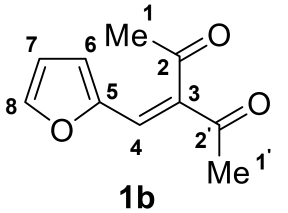 **1b**	-6.48	-2.57	2.6	0.61<br>0.56	-0.13	-0.23	0.17
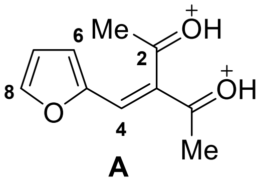 **A**	-8.30	-4.56	5.5	0.64<br>0.64	0.0	-0.08	0.34
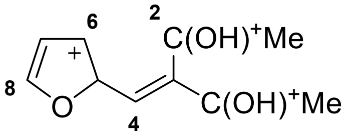 **B**	-10.36	-5.48	6.4	0.72<br>0.71	0.12	0.03	0.48
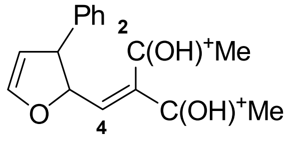 **Ca**	-6.96	-4.80	8.0	0.70<br>0.69	0.16	-0.23	0.16
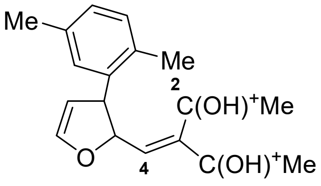 **Cb**	-6.88	-4.78	8.1	0.70<br>0.69	0.16	-0.24	0.16

Species	k(C^2)$_{LUMO}$,[c] %	k(C^4)$_{LUMO}$,[c] %	k(C^6)$_{LUMO}$,[c] %	k(C^8)$_{LUMO}$,[c] %
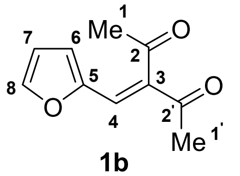 **1b**	7.5<br>8.6	24.3	9.5	6.1
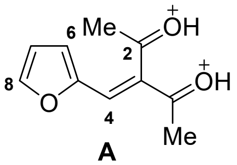 **A**	11.1<br>32.8	7.3	6.7	4.3
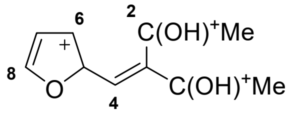 **B**	9.9<br>21.4	22.2	0.7	0.3
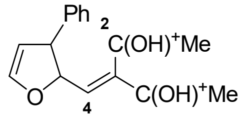 **Ca**	12.7<br>13.8	22.3	7.8	1.5
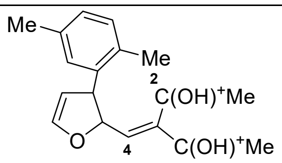 **Cb**	13.2<br>16.4	26.0	4.2	0.5

[a]Global electrophilicity index ω = (E_{HOMO} + E_{LUMO})^2/8(E_{LUMO} - E_{HOMO}).
[b]Natural charges.
[c]Contribution of atomic orbital into LUMO.

Compound 1b

Energy E(B3LYP) = -612.922683336 h, G^{298} = -612.786191 h, μ=2.78 D

Cartesian coordinates, Å

N	atom	x	y	z
1	C	-3.506247	0.610427	-0.052054
2	C	-2.112527	0.852939	-0.148630
3	C	-1.482709	-0.361926	-0.015719
4	O	-2.442750	-1.340709	0.152207
5	C	-3.648470	-0.732423	0.129123
6	H	-4.300207	1.333216	-0.114596
7	H	-1.636464	1.801797	-0.313640
8	H	-4.505320	-1.370178	0.250191
9	C	-0.137496	-0.845210	-0.012736
10	H	-0.077001	-1.923988	0.049015
11	C	1.023713	-0.152421	-0.060973
12	C	1.087275	1.357376	-0.125067
13	O	0.843129	1.941421	-1.163172
14	C	2.336728	-0.824951	-0.066887
15	O	3.355735	-0.144696	-0.163875
16	C	1.439530	2.078089	1.146038
17	H	0.656702	1.900499	1.887495
18	H	2.365950	1.678464	1.557355
19	H	1.535713	3.145138	0.963590
20	C	2.426138	-2.330716	0.032392
21	H	3.473943	-2.616207	0.030692
22	H	1.955742	-2.694609	0.945963
23	H	1.926422	-2.809363	-0.810257

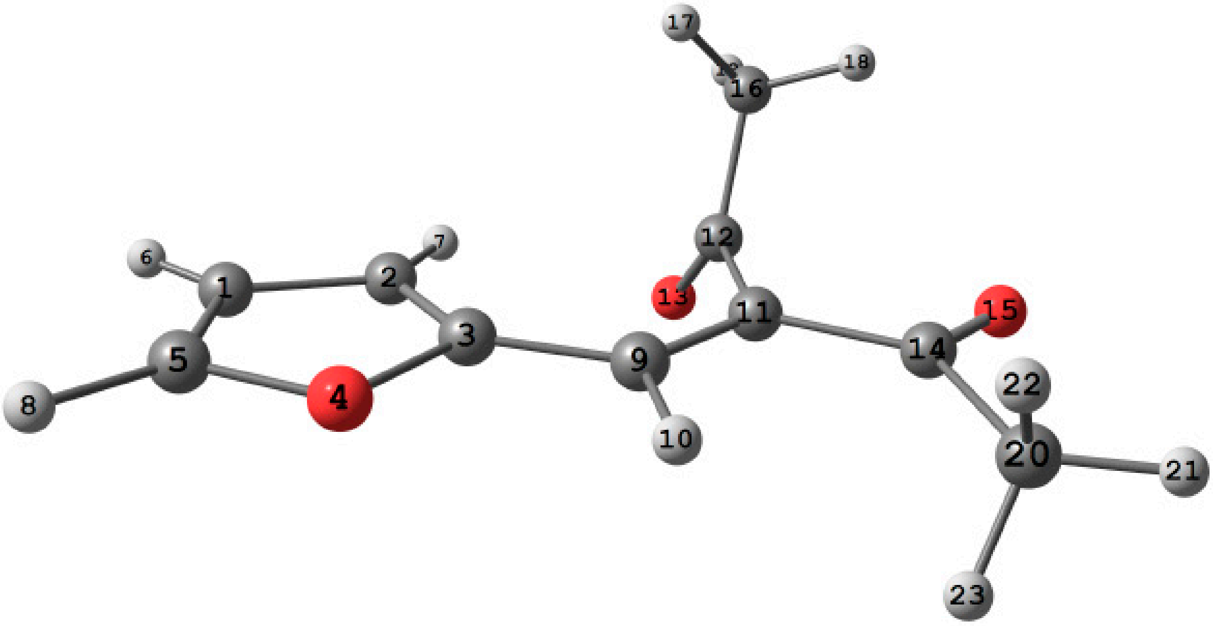

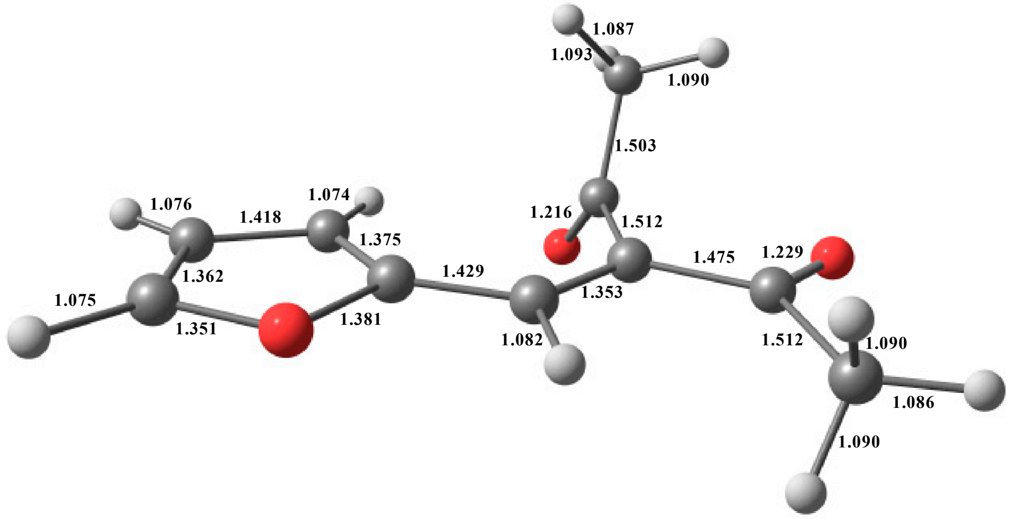

Summary of Natural Population Analysis:

Atom	No	Natural Charge	Core	Valence	Rydberg	Total
C	1	-0.29727	1.99916	4.27968	0.01843	6.29727
C	2	-0.22817	1.99904	4.21203	0.01710	6.22817
C	3	0.23394	1.99894	3.74350	0.02361	5.76606
O	4	-0.46174	1.99972	6.43799	0.02402	8.46174
C	5	0.16650	1.99909	3.80989	0.02452	5.83350
H	6	0.23870	0.00000	0.75982	0.00148	0.76130
H	7	0.24036	0.00000	0.75766	0.00198	0.75964
H	8	0.21514	0.00000	0.78339	0.00147	0.78486
C	9	-0.13305	1.99896	4.11652	0.01757	6.13305
H	10	0.23480	0.00000	0.76307	0.00214	0.76520
C	11	-0.21454	1.99880	4.18727	0.02847	6.21454
C	12	0.60581	1.99929	3.35442	0.04048	5.39419
O	13	-0.59042	1.99974	6.56387	0.02681	8.59042
C	14	0.55806	1.99925	3.41078	0.03192	5.44194
O	15	-0.62523	1.99976	6.59964	0.02583	8.62523
C	16	-0.66934	1.99923	4.65825	0.01186	6.66934
H	17	0.23776	0.00000	0.76061	0.00163	0.76224
H	18	0.24000	0.00000	0.75807	0.00193	0.76000
H	19	0.22365	0.00000	0.77403	0.00232	0.77635
C	20	-0.66941	1.99926	4.65849	0.01166	6.66941
H	21	0.22889	0.00000	0.76908	0.00203	0.77111
H	22	0.23197	0.00000	0.76643	0.00160	0.76803
H	23	0.23359	0.00000	0.76482	0.00159	0.76641
* Total *		0.00000	25.99024	67.68930	0.32046	94.00000

Natural Population: Core, Valence, Rydberg, Total.

Cation A

Energy E(B3LYP) =-613.74144596 h, G^{298} = -613.576675 h, μ=4.32 D

Cartesian coordinates, Å

N	atom	x	y	z
1	C	-3.645428	0.629654	0.288951
2	C	-2.304869	0.957167	0.310080
3	C	-1.572191	-0.203909	-0.034973
4	O	-2.515149	-1.224500	-0.262480
5	C	-3.709115	-0.710412	-0.075072
6	H	-4.483645	1.266096	0.506944
7	H	-1.886448	1.910587	0.562315
8	H	-4.548893	-1.369365	-0.222327
9	C	-0.284770	-0.658060	-0.134685
10	H	-0.283101	-1.721643	-0.322859
11	C	1.023953	-0.087691	-0.032349
12	C	1.279328	1.314937	-0.089785
13	O	0.223807	2.051950	-0.228443
14	C	2.095270	-1.027303	0.065829
15	O	3.295984	-0.561220	0.185616
16	C	2.594453	1.992873	-0.041662
17	H	2.468877	3.068559	-0.135880
18	H	3.098467	1.768392	0.898368
19	H	3.240950	1.630133	-0.840374
20	C	1.915924	-2.502714	0.057083
21	H	2.875089	-3.005004	0.144515
22	H	1.283156	-2.817472	0.886602
23	H	1.442591	-2.825676	-0.870723
24	H	3.967193	-1.259572	0.252510
25	H	0.433298	2.997865	-0.297128

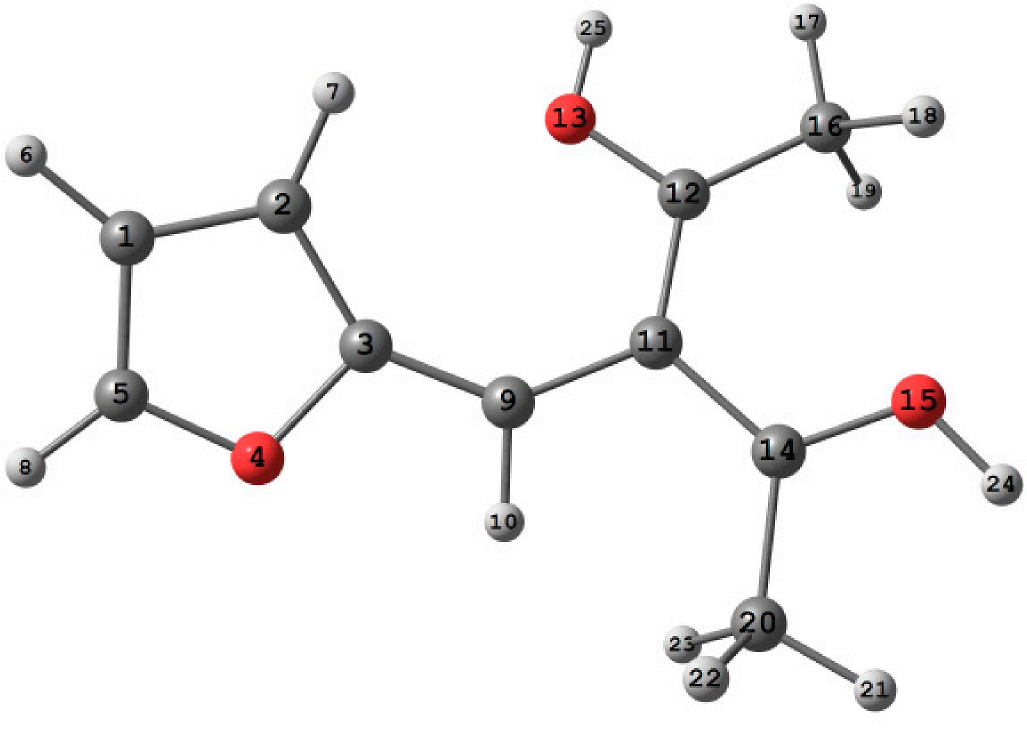

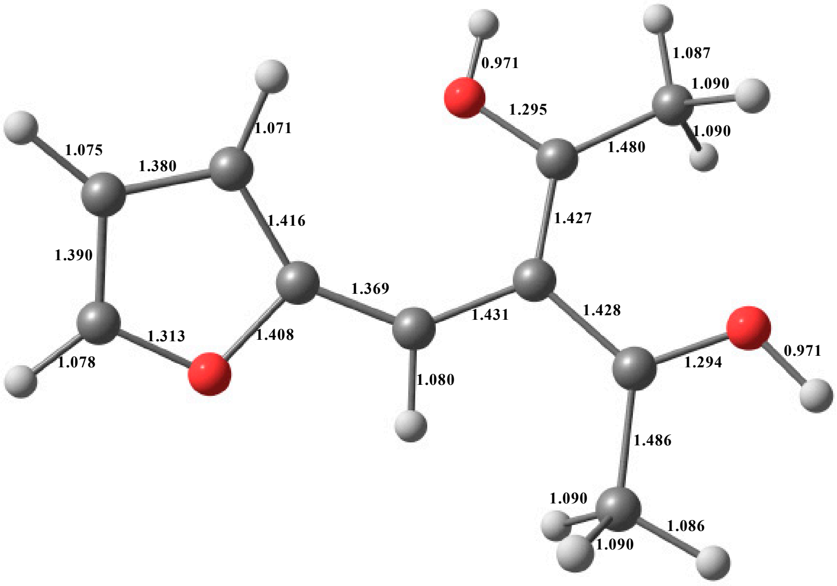

Summary of Natural Population Analysis:

Atom	No	Natural Charge	Core	Valence	Rydberg	Total
			Natural Population			
C	1	-0.27147	1.99916	4.25439	0.01792	6.27147
C	2	-0.08274	1.99906	4.06697	0.01671	6.08274
C	3	0.23420	1.99893	3.74479	0.02208	5.76580
O	4	-0.42433	1.99971	6.39979	0.02482	8.42433
C	5	0.34145	1.99919	3.63598	0.02338	5.65855
H	6	0.26282	0.00000	0.73575	0.00143	0.73718
H	7	0.26194	0.00000	0.73618	0.00188	0.73806
H	8	0.23758	0.00000	0.76113	0.00129	0.76242
C	9	-0.00444	1.99899	3.98749	0.01795	6.00444
H	10	0.25591	0.00000	0.74198	0.00211	0.74409
C	11	-0.27435	1.99881	4.25524	0.02031	6.27435
C	12	0.64426	1.99910	3.33445	0.02219	5.35574
O	13	-0.58160	1.99968	6.55639	0.02552	8.58160
C	14	0.64210	1.99911	3.33632	0.02247	5.35790
O	15	-0.57468	1.99968	6.54965	0.02536	8.57468
C	16	-0.66356	1.99922	4.64859	0.01575	6.66356
H	17	0.23832	0.00000	0.75973	0.00196	0.76168
H	18	0.28025	0.00000	0.71818	0.00158	0.71975
H	19	0.27942	0.00000	0.71898	0.00160	0.72058
C	20	-0.67681	1.99922	4.66334	0.01426	6.67681
H	21	0.24795	0.00000	0.75004	0.00201	0.75205
H	22	0.27597	0.00000	0.72269	0.00134	0.72403
H	23	0.27419	0.00000	0.72448	0.00134	0.72581
H	24	0.53896	0.00000	0.45827	0.00276	0.46104
H	25	0.53866	0.00000	0.45868	0.00266	0.46134
* Total *		2.00000	25.98987	67.71947	0.29066	94.00000

Cation B

Energy E(B3LYP) = -614.085518026 h, G^{298} = -613.908264 h, μ=2.41 D

Cartesian coordinates, Å

N	atom	x	y	z
1	C	-3.507485	-0.051569	0.854075
2	C	-2.278995	-0.609945	0.922490
3	C	-1.543640	-0.301188	-0.344500
4	O	-2.497261	0.516559	-1.091360
5	C	-3.576355	0.611463	-0.394894
6	H	-4.302405	-0.098468	1.577442
7	H	-1.860403	-1.198668	1.722508
8	H	-4.393521	1.166713	-0.834491
9	C	-0.302736	0.515670	-0.160398
10	H	-0.498853	1.578431	-0.162851
11	C	0.985078	0.109377	-0.014257
12	C	1.367464	-1.313829	-0.082392
13	O	0.424414	-2.099486	0.259409
14	C	2.004343	1.153720	0.161082
15	O	3.179191	0.719798	0.379380
16	C	2.662027	-1.832233	-0.529472
17	H	2.570395	-2.864688	-0.857949
18	H	3.092407	-1.210138	-1.310009
19	H	3.350988	-1.803286	0.323783
20	C	1.721631	2.597266	0.113301
21	H	2.630514	3.175514	0.249284
22	H	1.267609	2.854947	-0.845583
23	H	1.014105	2.866555	0.900490
24	H	3.855059	1.415326	0.492936
25	H	0.630144	-3.050396	0.163642
26	H	-1.394781	-1.199207	-0.948840

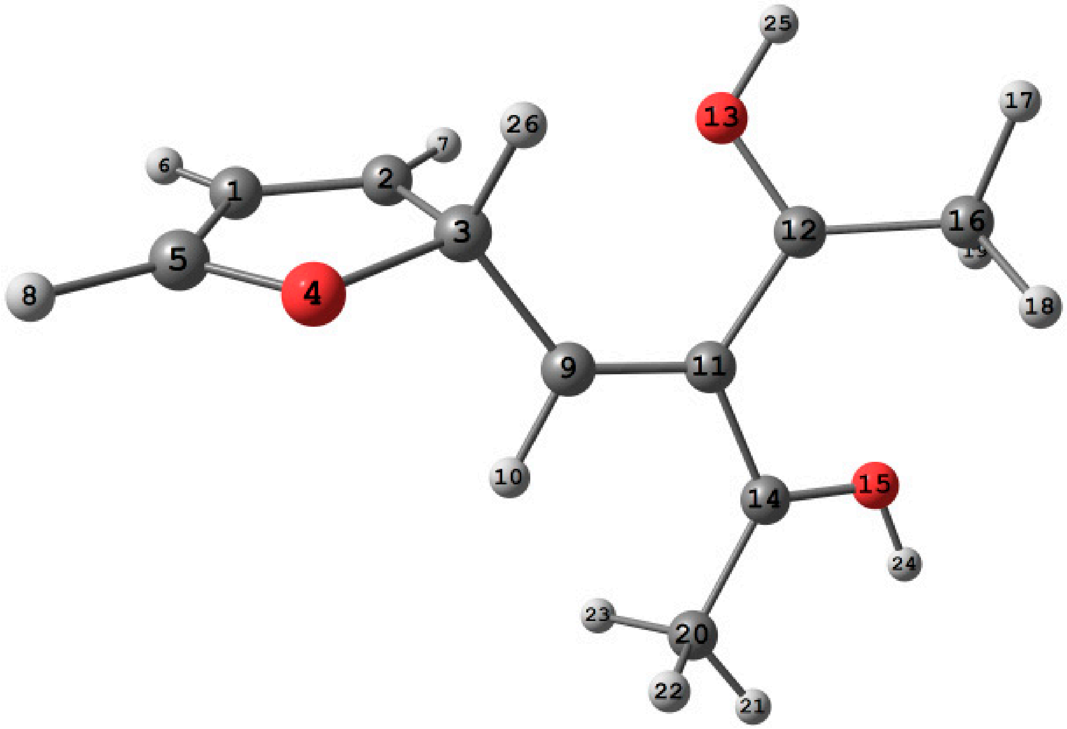

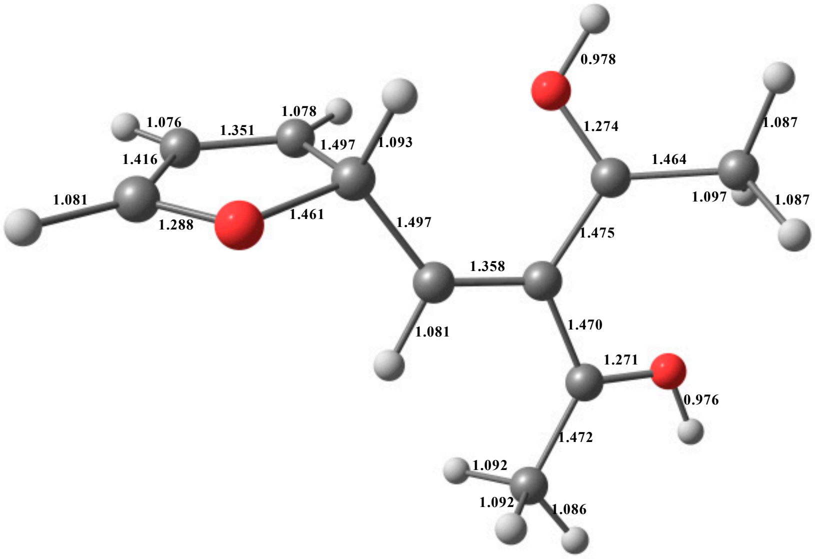

Summary of Natural Population Analysis:

Atom	No	Natural Charge	Natural Population Core	Valence	Rydberg	Total
C	1	-0.26415	1.99910	4.24627	0.01879	6.26415
C	2	0.03485	1.99910	3.94875	0.01731	5.96515
C	3	0.00247	1.99902	3.97485	0.02366	5.99753
O	4	-0.39160	1.99972	6.36623	0.02565	8.39160
C	5	0.47729	1.99926	3.49918	0.02427	5.52271
H	6	0.28596	0.00000	0.71252	0.00152	0.71404
H	7	0.27851	0.00000	0.71970	0.00179	0.72149
H	8	0.25752	0.00000	0.74132	0.00116	0.74248
C	9	0.12373	1.99898	3.85975	0.01753	5.87627
H	10	0.27202	0.00000	0.72595	0.00202	0.72798
C	11	-0.23088	1.99873	4.20925	0.02290	6.23088
C	12	0.71910	1.99917	3.25789	0.02384	5.28090
O	13	-0.53899	1.99966	6.51361	0.02573	8.53899
C	14	0.71329	1.99919	3.26455	0.02297	5.28671
O	15	-0.52754	1.99966	6.50239	0.02549	8.52754
C	16	-0.68737	1.99920	4.67188	0.01630	6.68737
H	17	0.26940	0.00000	0.72871	0.00188	0.73060
H	18	0.28443	0.00000	0.71406	0.00151	0.71557
H	19	0.31514	0.00000	0.68332	0.00154	0.68486
C	20	-0.69214	1.99920	4.67746	0.01547	6.69214
H	21	0.26679	0.00000	0.73136	0.00185	0.73321
H	22	0.29328	0.00000	0.70540	0.00133	0.70672
H	23	0.29589	0.00000	0.70277	0.00134	0.70411
H	24	0.55875	0.00000	0.43869	0.00256	0.44125
H	25	0.56124	0.00000	0.43624	0.00252	0.43876
H	26	0.32303	0.00000	0.67474	0.00223	0.67697
* Total *		3.00000	25.98999	67.70685	0.30317	94.00000

Cation Ca
Energy E(B3LYP) = -846.029063271 h, G^{298} = -845.76978 h, μ=21.9 D

Cartesian coordinates, Å

N	atom	x	y	z
1	C	-1.409579	2.511189	0.634708
2	C	-1.016618	1.047743	0.604457
3	C	0.007242	1.094378	-0.643004
4	O	-0.065028	2.418493	-1.200325
5	C	-0.858963	3.177256	-0.368162
6	H	-2.103057	2.913222	1.353562
7	H	-0.476004	0.773056	1.507943
8	H	-0.942737	4.213340	-0.650152
9	C	1.386142	0.915338	-0.170155
10	H	1.818743	1.844554	0.174899
11	C	2.196754	-0.201039	-0.112080
12	C	1.756846	-1.496761	-0.616455
13	O	0.484264	-1.622891	-0.679692
14	C	3.520264	-0.034290	0.454527
15	O	4.218813	-1.101283	0.575708
16	C	2.620371	-2.603502	-1.057750
17	H	2.067982	-3.274926	-1.711311
18	H	3.521028	-2.244530	-1.547040
19	H	2.934075	-3.165717	-0.171912
20	C	4.067133	1.256884	0.922272
21	H	5.107674	1.148437	1.214592
22	H	3.991048	2.014676	0.143658
23	H	3.498417	1.603988	1.788273
24	H	5.099040	-0.952348	0.964713
25	H	0.192392	-2.464375	-1.076155
26	H	-0.292037	0.389930	-1.409934
27	C	-2.170969	0.088286	0.431917
28	C	-2.330512	-0.974754	1.323281
29	C	-3.103696	0.254281	-0.596984
30	C	-3.399480	-1.855356	1.193240
31	C	-4.169952	-0.627075	-0.730827
32	C	-4.320753	-1.684447	0.164060
33	H	-1.615794	-1.112617	2.124015
34	H	-3.002535	1.077674	-1.291822
35	H	-3.511854	-2.671781	1.893101
36	H	-4.884655	-0.487419	-1.530020
37	H	-5.151505	-2.368487	0.059787

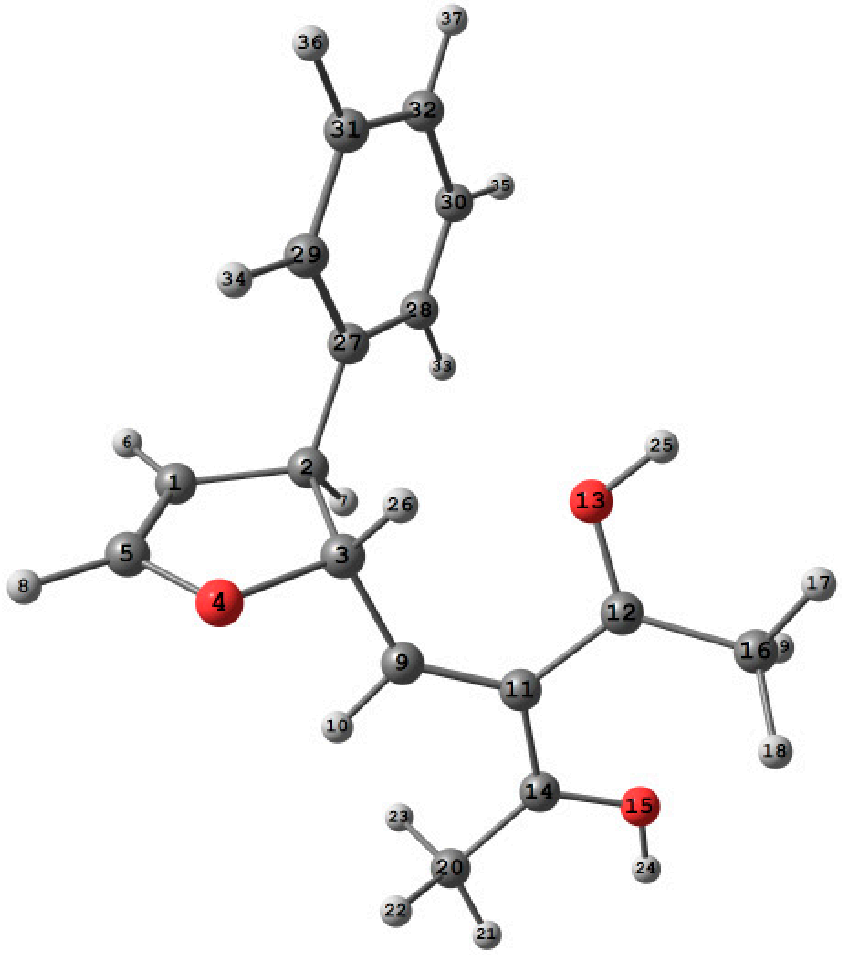
1
2
3
4
5
6
7
8
9
10
11
12
13
14
15
16
17
18
20
21
22
23
24
25
26
27
28
29
30
31
32
33
34
35
36
37

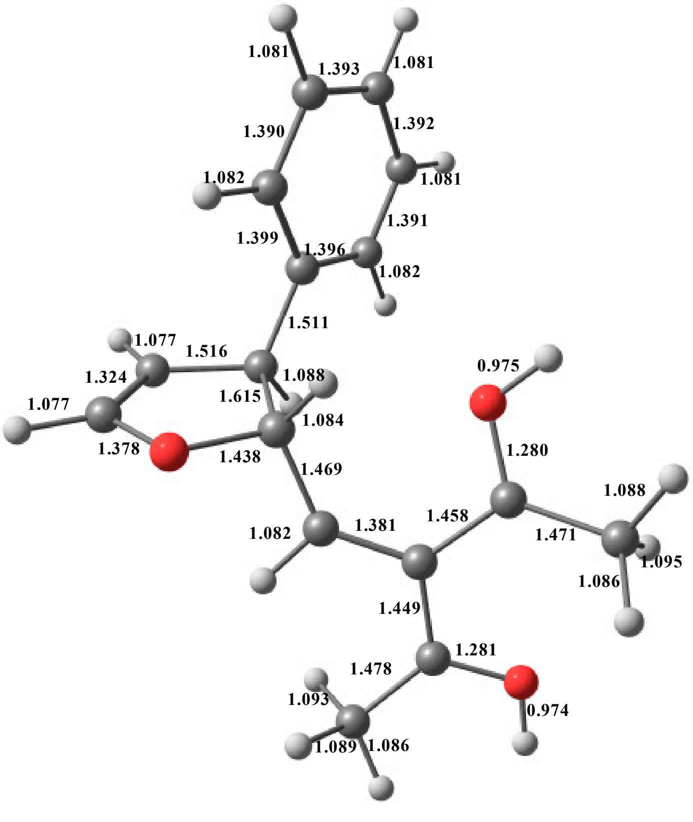
1.081
1.393
1.081
1.390
1.392
1.082
1.081
1.391
1.399
1.396
1.082
1.511
1.077
1.516
1.324
1.088
1.077
1.615
1.084
0.975
1.378
1.438
1.280
1.469
1.088
1.458
1.082
1.381
1.471
1.095
1.086
1.449
1.281
1.478
1.093
0.974
1.089
1.086

Summary of Natural Population Analysis:

Atom	No	Natural Charge	Natural Population Core	Valence	Rydberg	Total
C	1	-0.27182	1.99904	4.25376	0.01902	6.27182
C	2	-0.23429	1.99909	4.20811	0.02709	6.23429
C	3	0.05930	1.99902	3.91249	0.02919	5.94070
O	4	-0.54998	1.99977	6.52591	0.02430	8.54998
C	5	0.16058	1.99889	3.81751	0.02302	5.83942
H	6	0.24449	0.00000	0.75378	0.00173	0.75551
H	7	0.23622	0.00000	0.76098	0.00280	0.76378
H	8	0.22014	0.00000	0.77829	0.00158	0.77986
C	9	0.15710	1.99906	3.82594	0.01789	5.84290
H	10	0.24987	0.00000	0.74770	0.00243	0.75013
C	11	-0.28606	1.99875	4.26287	0.02443	6.28606
C	12	0.70002	1.99915	3.27768	0.02314	5.29998
O	13	-0.54338	1.99967	6.51688	0.02683	8.54338
C	14	0.68972	1.99915	3.28883	0.02229	5.31028
O	15	-0.54860	1.99967	6.52355	0.02539	8.54860
C	16	-0.67859	1.99921	4.66352	0.01586	6.67859
H	17	0.25720	0.00000	0.74081	0.00199	0.74280
H	18	0.27676	0.00000	0.72172	0.00152	0.72324
H	19	0.30184	0.00000	0.69660	0.00156	0.69816
C	20	-0.68369	1.99921	4.66967	0.01481	6.68369
H	21	0.25719	0.00000	0.74089	0.00193	0.74281
H	22	0.27974	0.00000	0.71885	0.00141	0.72026
H	23	0.29070	0.00000	0.70791	0.00138	0.70930
H	24	0.54876	0.00000	0.44857	0.00267	0.45124
H	25	0.54922	0.00000	0.44809	0.00269	0.45078
H	26	0.25769	0.00000	0.74012	0.00219	0.74231
C	27	-0.04998	1.99897	4.03081	0.02020	6.04998
C	28	-0.18879	1.99904	4.17110	0.01865	6.18879
C	29	-0.19600	1.99904	4.17898	0.01799	6.19600
C	30	-0.19868	1.99915	4.17942	0.02011	6.19868
C	31	-0.19771	1.99916	4.17837	0.02018	6.19771
C	32	-0.19455	1.99915	4.17530	0.02010	6.19455
H	33	0.21607	0.00000	0.78207	0.00187	0.78393
H	34	0.21891	0.00000	0.77917	0.00192	0.78109
H	35	0.21717	0.00000	0.78112	0.00172	0.78283
H	36	0.21699	0.00000	0.78128	0.00173	0.78301
H	37	0.21645	0.00000	0.78199	0.00155	0.78355
* Total *		2.00000	37.98418	97.57066	0.44516	136.00000

Cation Cb
Energy E(B3LYP) = -924.682568419 h, G^{298} = -924.373423 h, μ=24.6 D

Cartesian coordinates, Å

N	atom	x	y	z
1	C	0.852370	2.467937	-1.210015
2	C	0.617705	1.037766	-0.758706
3	C	-0.512442	1.311076	0.351789
4	O	-0.618313	2.738589	0.505731
5	C	0.142318	3.319295	-0.486965
6	H	1.549290	2.726544	-1.988852
7	H	0.171301	0.468754	-1.571349
8	H	0.079620	4.393690	-0.529406
9	C	-1.826445	0.854643	-0.124119
10	H	-2.318348	1.598071	-0.736526
11	C	-2.524426	-0.313428	0.110198
12	C	-1.988461	-1.367612	0.962956
13	O	-0.711113	-1.356471	1.055785
14	C	-3.829786	-0.449621	-0.502276
15	O	-4.414310	-1.577066	-0.330612
16	C	-2.766013	-2.371727	1.706726
17	H	-2.185817	-2.751905	2.544777
18	H	-3.717497	-1.974691	2.047334
19	H	-2.981576	-3.207799	1.032482
20	C	-4.478239	0.596175	-1.320777
21	H	-5.487972	0.300229	-1.591556
22	H	-4.514059	1.543636	-0.785290
23	H	-3.903550	0.749380	-2.237463
24	H	-5.286993	-1.636561	-0.758287
25	H	-0.355807	-2.021958	1.673622
26	H	-0.233238	0.885602	1.307967
27	C	1.846180	0.269229	-0.313544
28	C	2.188130	-0.848168	-1.087061
29	C	2.662799	0.626574	0.779798
30	C	3.313669	-1.626317	-0.835684
31	C	3.788985	-0.163094	1.030248
32	C	4.113983	-1.262841	0.248842
33	H	1.547893	-1.114431	-1.919258
34	H	4.427854	0.096218	1.864458
35	H	4.996929	-1.842654	0.486129
36	C	2.385671	1.790228	1.701401
37	H	3.247146	1.972369	2.340527
38	H	2.171866	2.707734	1.158199
39	H	1.537263	1.596896	2.361243
40	C	3.665998	-2.803793	-1.708518
41	H	3.953029	-3.668010	-1.109027
42	H	2.829663	-3.091374	-2.343618
43	H	4.510918	-2.568086	-2.359107

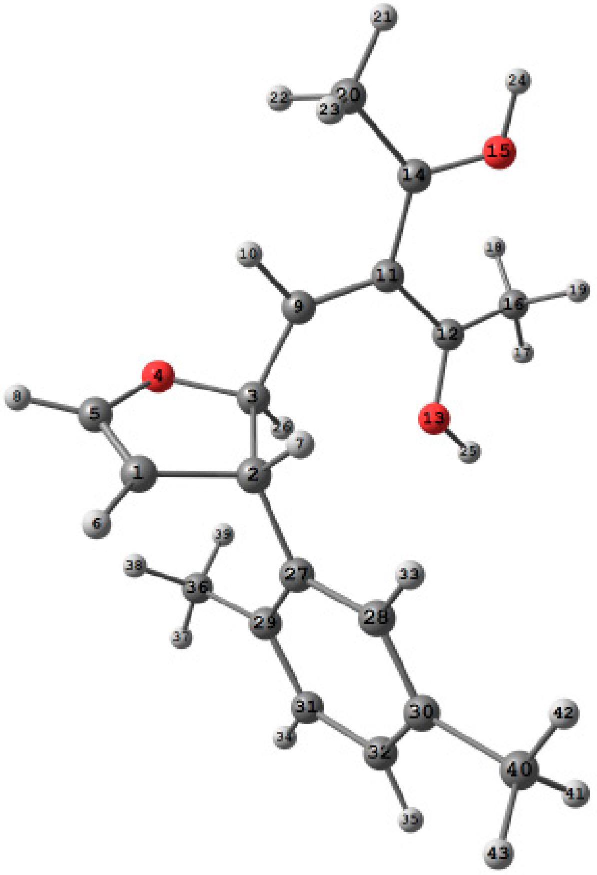
21
22
23
20
24
15
14
10
18
11
19
16
9
12
17
4
8
5
3
13
26
7
25
1
2
6
39
38
36
27
33
37
29
28
31
30
42
34
32
40
41
35
43

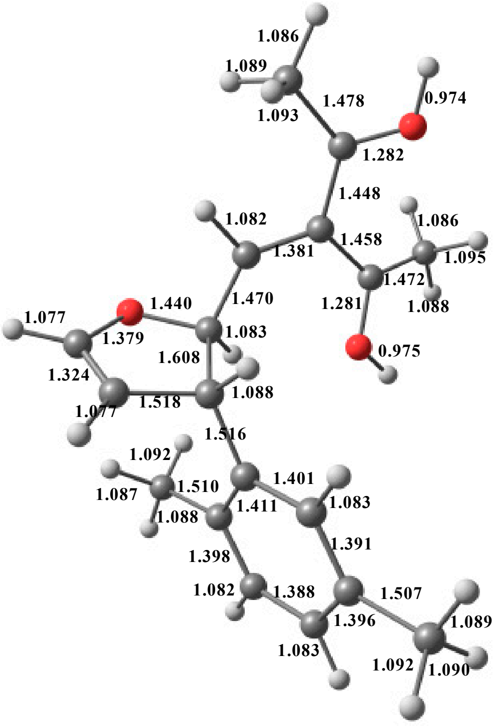
1.086
1.089
0.974
1.478
1.093
1.282
1.448
1.082
1.086
1.381
1.458
1.095
1.470
1.472
1.440
1.281
1.088
1.077
1.083
1.379
0.975
1.608
1.324
1.088
1.077
1.518
1.516
1.092
1.510
1.401
1.087
1.411
1.083
1.088
1.391
1.398
1.082
1.388
1.507
1.396
1.089
1.083
1.092
1.090

Summary of Natural Population Analysis:

Atom	No	Natural Charge	Natural Population Core	Valence	Rydberg	Total
C	1	-0.26923	1.99903	4.25163	0.01857	6.26923
C	2	-0.23541	1.99909	4.20905	0.02727	6.23541
C	3	0.05687	1.99901	3.91585	0.02828	5.94313
O	4	-0.55196	1.99977	6.52771	0.02449	8.55196
C	5	0.15700	1.99888	3.82093	0.02319	5.84300
H	6	0.24387	0.00000	0.75437	0.00176	0.75613
H	7	0.23484	0.00000	0.76235	0.00281	0.76516
H	8	0.22068	0.00000	0.77772	0.00160	0.77932
C	9	0.15614	1.99906	3.82686	0.01794	5.84386
H	10	0.24993	0.00000	0.74773	0.00235	0.75007
C	11	-0.28660	1.99875	4.26350	0.02436	6.28660
C	12	0.70077	1.99915	3.27707	0.02300	5.29923
O	13	-0.54244	1.99967	6.51627	0.02651	8.54244
C	14	0.68857	1.99915	3.29008	0.02220	5.31143
O	15	-0.54935	1.99967	6.52434	0.02534	8.54935
C	16	-0.67932	1.99921	4.66432	0.01579	6.67932
H	17	0.25874	0.00000	0.73928	0.00198	0.74126
H	18	0.27533	0.00000	0.72316	0.00151	0.72467
H	19	0.30192	0.00000	0.69651	0.00157	0.69808
C	20	-0.68339	1.99921	4.66943	0.01475	6.68339
H	21	0.25700	0.00000	0.74108	0.00192	0.74300
H	22	0.27993	0.00000	0.71867	0.00140	0.72007
H	23	0.29043	0.00000	0.70819	0.00138	0.70957
H	24	0.54854	0.00000	0.44880	0.00267	0.45146
H	25	0.54920	0.00000	0.44810	0.00270	0.45080
H	26	0.25371	0.00000	0.74330	0.00299	0.74629
C	27	-0.04437	1.99889	4.02365	0.02183	6.04437
C	28	-0.18585	1.99895	4.16918	0.01772	6.18585
C	29	-0.01948	1.99894	4.00432	0.01622	6.01948
C	30	-0.02889	1.99902	4.01284	0.01702	6.02889
C	31	-0.19415	1.99906	4.17568	0.01940	6.19415
C	32	-0.18669	1.99904	4.16865	0.01900	6.18669
H	33	0.20883	0.00000	0.78910	0.00207	0.79117
H	34	0.21157	0.00000	0.78643	0.00200	0.78843
H	35	0.21276	0.00000	0.78541	0.00183	0.78724
C	36	-0.60095	1.99929	4.58861	0.01305	6.60095
H	37	0.21690	0.00000	0.78147	0.00164	0.78310
H	38	0.22055	0.00000	0.77755	0.00189	0.77945
H	39	0.21222	0.00000	0.78613	0.00165	0.78778
C	40	-0.59001	1.99929	4.57914	0.01158	6.59001
H	41	0.21436	0.00000	0.78411	0.00152	0.78564
H	42	0.21026	0.00000	0.78827	0.00148	0.78974
H	43	0.21721	0.00000	0.78121	0.00158	0.78279
* Total *		2.00000	41.98212	109.54807	0.46980	152.00000

Cation D
Energy E(B3LYP) = -925.06446033 h, G^{298} = -924.740374 h, μ=19.2 D

Cartesian coordinates, Å

N	atom	x	y	z
1	C	-1.209366	-1.660731	-0.803768
2	C	-0.452090	-0.445378	-0.669097
3	C	0.394011	-0.594096	0.601554
4	O	-0.080785	-1.763789	1.251586
5	C	-0.866465	-2.565997	0.345340
6	H	-1.522671	-2.080759	-1.744622
7	H	-0.069079	0.118142	-1.503215
8	H	-1.711817	-2.968381	0.896296
9	C	1.847100	-0.773382	0.275464
10	H	2.115839	-1.817631	0.195170
11	C	2.861227	0.141150	0.152985
12	C	2.618933	1.581275	0.284022
13	O	1.386623	1.899202	0.162553
14	C	4.202698	-0.377676	-0.099627
15	O	5.103908	0.500416	-0.308933
16	C	3.621732	2.622754	0.543913
17	H	3.144100	3.545858	0.862087
18	H	4.354740	2.296527	1.278413
19	H	4.166765	2.813448	-0.386612
20	C	4.543313	-1.812551	-0.143679
21	H	5.607911	-1.948061	-0.309847
22	H	4.261335	-2.298846	0.791083
23	H	3.998427	-2.300063	-0.954755
24	H	5.990346	0.129437	-0.473961
25	H	1.205053	2.847781	0.299612
26	H	0.264322	0.242650	1.286666
27	C	-2.012262	-0.255927	-0.443185
28	C	-2.742302	0.305526	-1.559290
29	C	-2.554235	-0.151922	0.881524
30	C	-3.963020	0.897563	-1.281785
31	C	-3.765232	0.444726	1.123975
32	C	-4.459406	0.965873	0.013475
33	H	-2.002166	-0.572412	1.708024
34	H	-4.539683	1.319020	-2.091698
35	H	-5.419603	1.438934	0.174066
36	H	-0.274942	-3.395973	-0.048017
37	C	-2.227050	0.290226	-2.961645
38	H	-2.988622	0.650941	-3.646152
39	H	-1.355193	0.941749	-3.060483
40	H	-1.919260	-0.703469	-3.287860
41	C	-4.348607	0.544223	2.506129
42	H	-3.690775	0.094349	3.245614
43	H	-4.512934	1.586857	2.779444
44	H	-5.313944	0.039337	2.551264

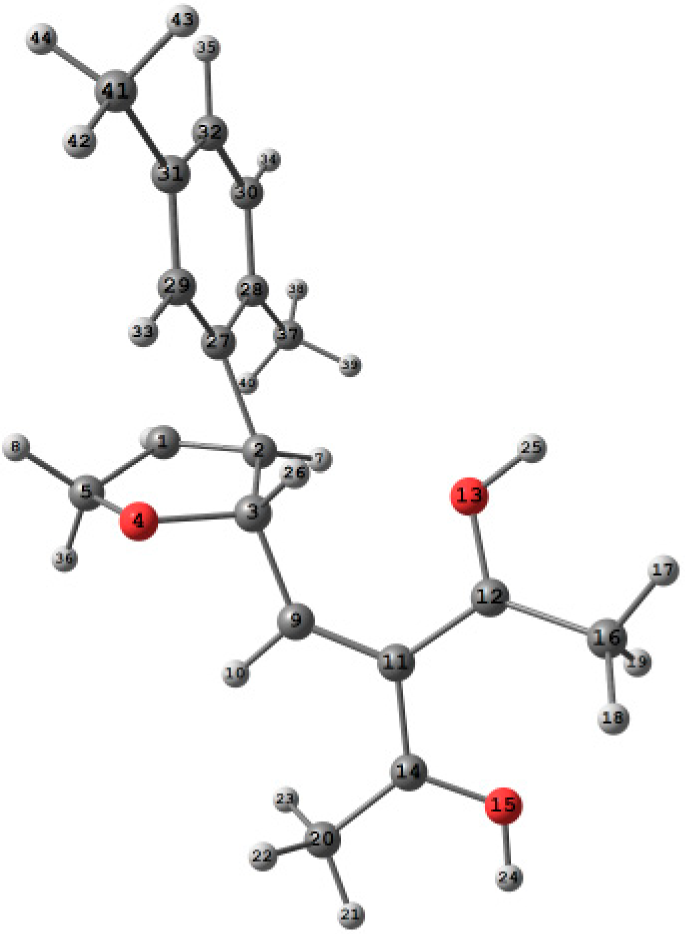
44
43
35
41
32
42
31
34
30
29
28
38
33
27
37
39
40
1
2
8
7
26
5
4
3
36
25
13
17
12
9
16
11
19
10
18
14
23
15
20
22
24
21

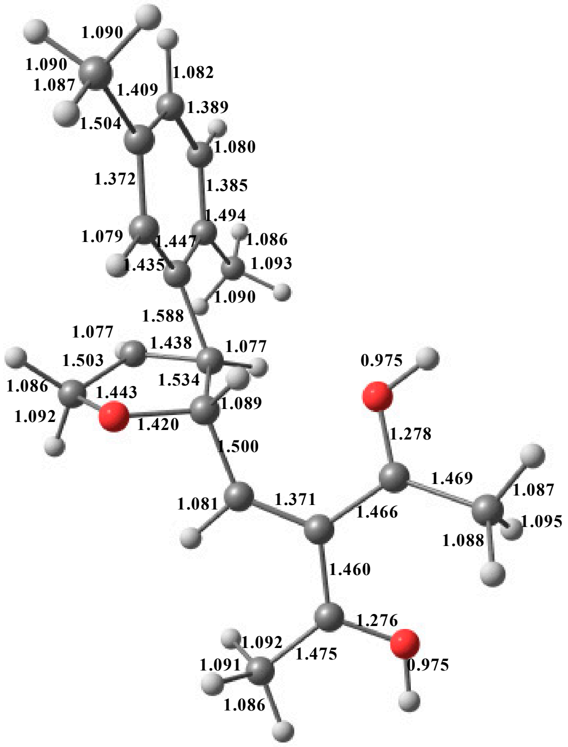
1.090
1.090
1.087
1.082
1.409
1.389
1.504
1.080
1.372
1.385
1.494
1.079
1.447
1.086
1.435
1.093
1.090
1.588
1.077
1.438
1.077
1.503
1.534
0.975
1.086
1.443
1.089
1.092
1.420
1.278
1.500
1.469
1.087
1.081
1.371
1.466
1.095
1.088
1.460
1.276
1.092
1.475
1.091
0.975
1.086

Summary of Natural Population Analysis:

Atom	No	Natural Charge	Natural Population: Core	Valence	Rydberg	Total
C	1	-0.08605	1.99906	4.06719	0.01981	6.08605
C	2	-0.16196	1.99893	4.13587	0.02715	6.16196
C	3	0.05175	1.99904	3.91852	0.03069	5.94825
O	4	-0.57857	1.99978	6.55842	0.02037	8.57857
C	5	-0.04228	1.99912	4.02248	0.02068	6.04228
H	6	0.27814	0.00000	0.71991	0.00195	0.72186
H	7	0.28343	0.00000	0.71467	0.00190	0.71657
H	8	0.22871	0.00000	0.76981	0.00148	0.77129
C	9	0.16849	1.99906	3.81402	0.01843	5.83151
H	10	0.25335	0.00000	0.74438	0.00226	0.74665
C	11	-0.26949	1.99875	4.24707	0.02367	6.26949
C	12	0.70776	1.99916	3.27028	0.02280	5.29224
O	13	-0.54355	1.99966	6.51823	0.02566	8.54355
C	14	0.70468	1.99917	3.27380	0.02235	5.29532
O	15	-0.53754	1.99966	6.51237	0.02551	8.53754
C	16	-0.68083	1.99921	4.66572	0.01590	6.68083
H	17	0.25839	0.00000	0.73968	0.00193	0.74161
H	18	0.28423	0.00000	0.71428	0.00149	0.71577
H	19	0.30615	0.00000	0.69236	0.00149	0.69385
C	20	-0.68729	1.99921	4.67284	0.01524	6.68729
H	21	0.26039	0.00000	0.73773	0.00188	0.73961
H	22	0.28825	0.00000	0.71041	0.00134	0.71175
H	23	0.29142	0.00000	0.70723	0.00135	0.70858
H	24	0.55321	0.00000	0.44416	0.00264	0.44679
H	25	0.55450	0.00000	0.44281	0.00269	0.44550
H	26	0.26888	0.00000	0.72902	0.00210	0.73112
C	27	-0.11835	1.99891	4.09481	0.02463	6.11835
C	28	0.16471	1.99896	3.81949	0.01685	5.83529
C	29	-0.06235	1.99891	4.04601	0.01743	6.06235
C	30	-0.21476	1.99901	4.19572	0.02003	6.21476
C	31	-0.03608	1.99902	4.02013	0.01693	6.03608
C	32	-0.01295	1.99908	3.99629	0.01758	6.01295
H	33	0.24082	0.00000	0.75640	0.00278	0.75918
H	34	0.23924	0.00000	0.75890	0.00187	0.76076
H	35	0.23285	0.00000	0.76566	0.00149	0.76715
H	36	0.22501	0.00000	0.77326	0.00173	0.77499
C	37	-0.61868	1.99926	4.60534	0.01408	6.61868
H	38	0.23888	0.00000	0.75955	0.00156	0.76112
H	39	0.24491	0.00000	0.75355	0.00154	0.75509
H	40	0.23573	0.00000	0.76280	0.00147	0.76427
C	41	-0.59193	1.99928	4.58098	0.01167	6.59193
H	42	0.22083	0.00000	0.77789	0.00129	0.77917
H	43	0.22881	0.00000	0.76983	0.00137	0.77119
H	44	0.22914	0.00000	0.76948	0.00138	0.77086
* Total *		3.00000	41.98223	109.54933	0.46844	152.00000

Cation E
Energy E(B3LYP) = -925.071113424 h, G^{298} = -924.747213 h, μ=15.8 D

Cartesian coordinates, Å

N	atom	x	y	z
1	C	1.337341	2.488373	0.202747
2	C	1.100850	1.013363	0.320108
3	C	-0.358177	0.848571	0.716626
4	O	-0.904310	2.160304	0.841076
5	C	-0.041646	3.130712	0.196338
6	H	1.904880	2.800573	1.085921
7	H	-0.101083	4.035669	0.792170
8	C	-1.276872	0.338479	-0.366924
9	H	-0.949869	0.598378	-1.363787
10	C	-2.539629	-0.191081	-0.265131
11	C	-3.173378	-0.497366	1.022938
12	O	-2.356038	-0.815118	1.950856
13	C	-3.276876	-0.400802	-1.501353
14	O	-4.382702	-1.029882	-1.376256
15	C	-4.613785	-0.423598	1.304722
16	H	-4.777123	-0.205435	2.358525
17	H	-5.117814	0.304987	0.677967
18	H	-5.051095	-1.405965	1.092104
19	C	-2.806795	0.017432	-2.836347
20	H	-3.574060	-0.158686	-3.584858
21	H	-2.535047	1.072260	-2.836547
22	H	-1.920344	-0.559470	-3.110586
23	H	-4.860517	-1.163482	-2.214784
24	H	-2.777236	-0.950088	2.821063
25	H	-0.449072	0.335553	1.667468
26	C	2.081773	0.044276	0.163271
27	C	3.398205	0.508912	-0.198348
28	C	1.886920	-1.380964	0.364514
29	C	4.469188	-0.329450	-0.355638
30	C	2.987501	-2.203383	0.200477
31	C	4.236989	-1.704483	-0.148691
32	H	3.550448	1.564141	-0.351649
33	H	2.872501	-3.266824	0.346884
34	H	5.058318	-2.399331	-0.266851
35	H	-0.404212	3.335641	-0.808681
36	H	1.928886	2.754248	-0.669294
37	C	0.589374	-2.011744	0.746644
38	H	-0.179579	-1.834587	-0.006032
39	H	0.705714	-3.088198	0.830769
40	H	0.227137	-1.645876	1.707243
41	C	5.835743	0.167992	-0.733077
42	H	6.563087	-0.089570	0.037380
43	H	6.168696	-0.294940	-1.662485
44	H	5.841440	1.247143	-0.864605

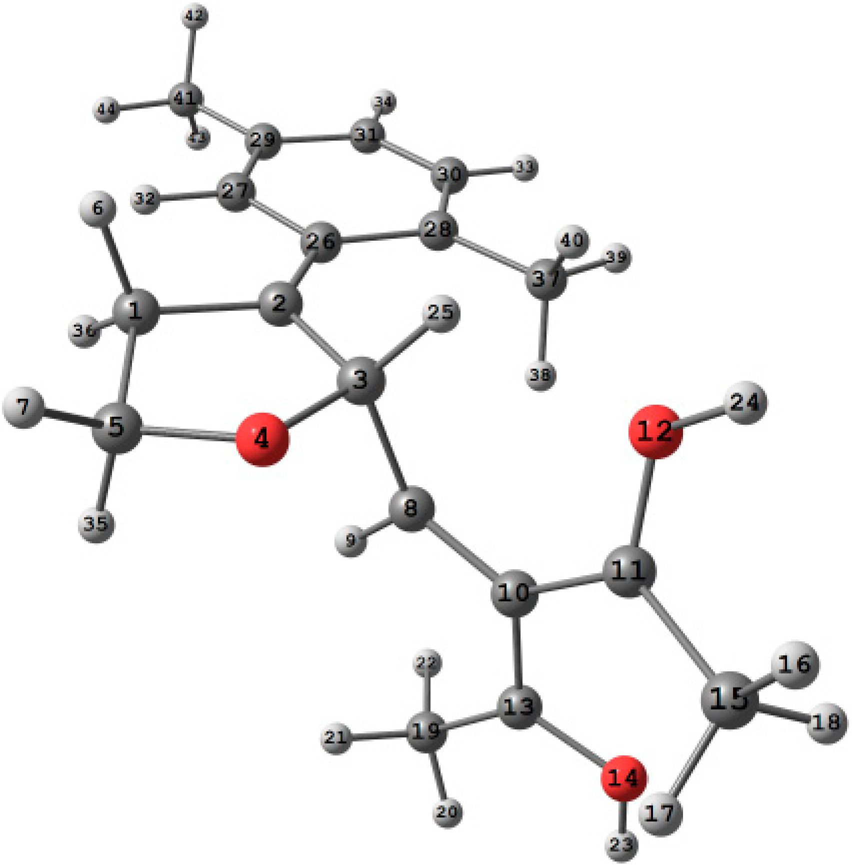
42
44
41
43
34
29
31
30
33
32
27
26
28
40
39
6
37
1
2
25
36
3
38
7
5
4
12
24
35
8
9
11
10
16
15
22
13
18
21
19
14
17
20
23

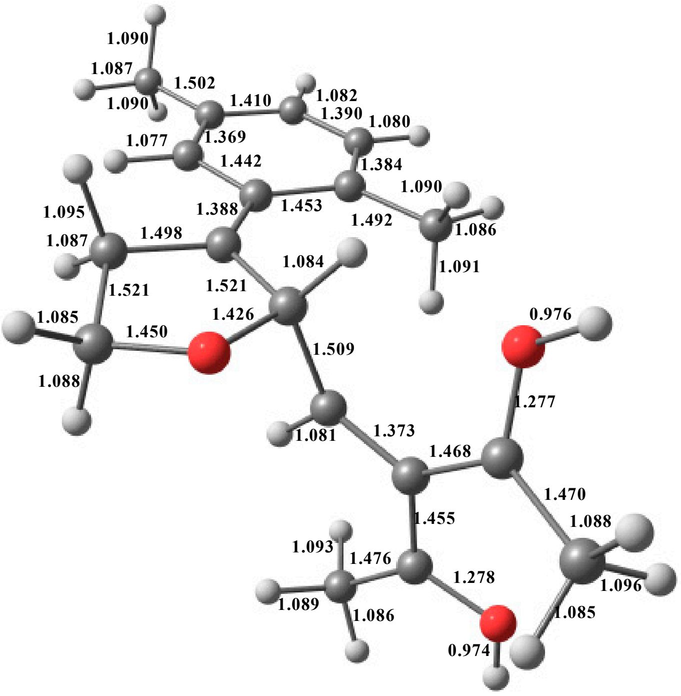
1.090
1.087
1.502
1.410
1.082
1.090
1.390
1.080
1.077
1.369
1.442
1.384
1.090
1.388
1.453
1.095
1.492
1.086
1.087
1.498
1.084
1.091
1.521
1.521
1.426
1.085
1.450
1.509
0.976
1.088
1.277
1.081
1.373
1.468
1.470
1.455
1.088
1.093
1.476
1.278
1.096
1.089
1.086
1.085
0.974

Summary of Natural Population Analysis:

Atom	No	Natural Charge	Core	Valence	Rydberg	Total
C	1	-0.55067	1.99914	4.48764	0.06389	6.55067
C	2	-0.24081	1.99903	3.91645	0.32532	6.24081
C	3	0.64675	1.99894	2.25134	1.10298	5.35325
O	4	-0.81970	1.99967	6.75974	0.06029	8.81970
C	5	-0.03706	1.99924	3.98962	0.04820	6.03706
H	6	0.28094	0.00000	0.71210	0.00696	0.71906
H	7	0.22180	0.00000	0.77416	0.00403	0.77820
C	8	-0.00615	1.99890	3.94722	0.06003	6.00615
H	9	0.24130	0.00000	0.74690	0.01180	0.75870
C	10	-0.30694	1.99871	4.25207	0.05617	6.30694
C	11	0.68913	1.99916	3.26165	0.05005	5.31087
O	12	-0.54791	1.99966	6.51603	0.03222	8.54791
C	13	0.68850	1.99915	3.28306	0.02928	5.31150
O	14	-0.54360	1.99966	6.51671	0.02722	8.54360
C	15	-0.68999	1.99921	4.66897	0.02181	6.68999
H	16	0.26673	0.00000	0.73124	0.00203	0.73327
H	17	0.27583	0.00000	0.72260	0.00157	0.72417
H	18	0.30736	0.00000	0.69042	0.00222	0.69264
C	19	-0.69035	1.99921	4.67216	0.01898	6.69035
H	20	0.26187	0.00000	0.73627	0.00186	0.73813
H	21	0.28199	0.00000	0.71641	0.00159	0.71801
H	22	0.29356	0.00000	0.70476	0.00168	0.70644
H	23	0.55090	0.00000	0.44525	0.00385	0.44910
H	24	0.55313	0.00000	0.44391	0.00296	0.44687
H	25	0.11161	0.00000	0.85273	0.03567	0.88839
C	26	0.02228	1.99883	2.80407	1.17482	5.97772
C	27	-0.39564	1.99889	4.21363	0.18312	6.39564
C	28	-0.34904	1.99888	4.13741	0.21275	6.34904
C	29	-0.13033	1.99898	4.03264	0.09871	6.13033
C	30	0.70273	1.99901	2.96987	0.32839	5.29727
C	31	-0.18680	1.99903	4.06839	0.11939	6.18680
H	32	0.21704	0.00000	0.77219	0.01077	0.78296
H	33	-0.36172	0.00000	1.32991	0.03181	1.36172
H	34	0.22360	0.00000	0.77117	0.00523	0.77640
H	35	0.19922	0.00000	0.79684	0.00394	0.80078
H	36	0.26713	0.00000	0.72385	0.00902	0.73287
C	37	-0.66723	1.99923	4.61125	0.05675	6.66723
H	38	0.22324	0.00000	0.76898	0.00778	0.77676
H	39	0.24019	0.00000	0.75561	0.00419	0.75981
H	40	0.23025	0.00000	0.76049	0.00926	0.76975
C	41	-0.59695	1.99928	4.58192	0.01576	6.59695
H	42	0.23006	0.00000	0.76829	0.00165	0.76994
H	43	0.22974	0.00000	0.76856	0.00170	0.77026
H	44	0.21962	0.00000	0.77688	0.00350	0.78038
* Total *		1.55564	41.98180	107.21135	4.25120	153.44436

Cation G
Energy E(B3LYP) = -846. 340462203 h, G^{298} = -846.071763 h, μ=23.1 D

Cartesian coordinates, Å

N	atom	x	y	z
1	C	1.114979	-2.112326	-0.604212
2	C	1.222341	-0.818077	0.155373
3	C	-0.048697	-0.063713	-0.573819
4	O	-0.837069	-1.099863	-1.105071
5	C	-0.064460	-2.229771	-1.205824
6	C	2.536418	-0.137818	0.140846
7	C	3.322065	-0.096378	-1.023989
8	C	4.573625	0.491253	-0.997739
9	C	5.057446	1.051412	0.188300
10	C	4.284512	1.022084	1.348519
11	C	3.029390	0.436904	1.324614
12	C	-0.756957	0.894752	0.181482
13	C	-2.186459	0.764000	0.637584
14	C	-2.604830	-0.609447	1.128628
15	O	-1.667503	-1.202687	1.735094
16	C	-3.028689	1.358079	-0.492017
17	O	-3.312355	0.543550	-1.415747
18	C	-3.428030	2.757402	-0.476587
19	C	-3.958010	-1.142068	1.025838
20	H	1.927320	-2.816816	-0.655496
21	H	0.889436	-0.955756	1.179766
22	H	-0.510118	-3.039830	-1.756199
23	H	-0.260344	1.831909	0.390650
24	H	-4.110824	-1.989635	1.687277
25	H	-4.693161	-0.359046	1.219944
26	H	-4.113896	-1.463841	-0.011300
27	H	-3.842920	3.087614	-1.424048
28	H	-4.185547	2.857563	0.315014
29	H	-2.595904	3.389595	-0.161601
30	H	-3.816034	0.926497	-2.162566
31	H	-1.914649	-2.054880	2.149279
32	H	2.950724	-0.524840	-1.945388
33	H	4.661012	1.456890	2.262861
34	H	6.032719	1.517007	0.202703
35	H	-2.323463	1.406078	1.517254
36	H	5.175587	0.520609	-1.894191
37	H	2.425245	0.414719	2.221357
38	H	0.452380	0.470441	-1.391497

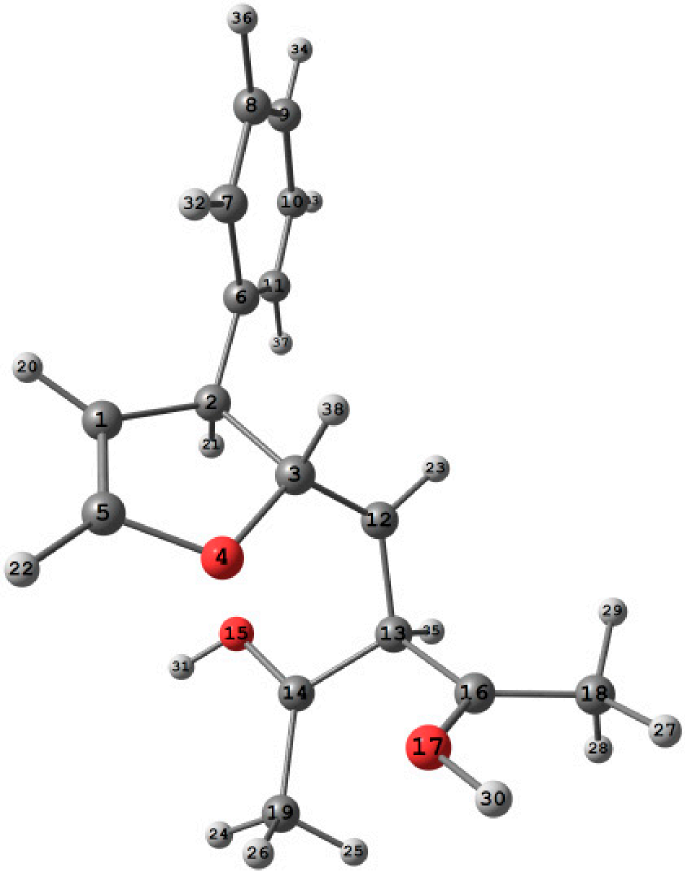
36
34
8
9
32
7
10
6
11
37
20
2
1
21
38
3
23
12
5
4
22
15
13
35
29
31
14
16
18
17
27
28
30
19
24
26
25

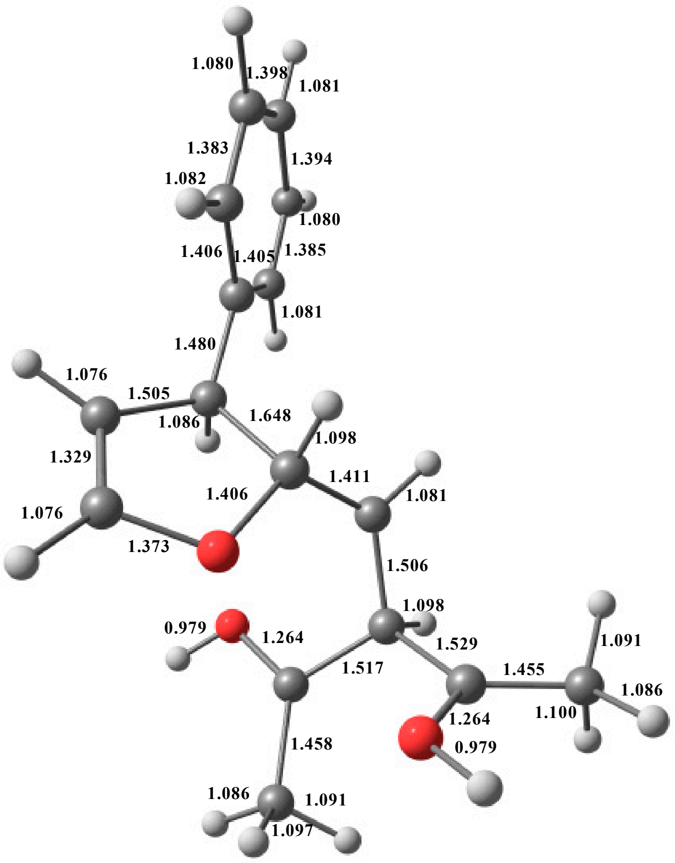
1.080
1.398
1.081
1.383
1.394
1.082
1.080
1.406
1.405
1.385
1.081
1.480
1.076
1.505
1.086
1.648
1.098
1.329
1.411
1.406
1.081
1.076
1.373
1.506
1.098
0.979
1.264
1.529
1.091
1.517
1.455
1.086
1.100
1.264
0.979
1.458
1.086
1.091
1.097

Summary of Natural Population Analysis:

Atom	No	Natural Charge	Natural Population Core	Valence	Rydberg	Total
C	1	-0.21142	1.99907	4.19374	0.01861	6.21142
C	2	-0.18902	1.99904	4.16598	0.02400	6.18902
C	3	0.02229	1.99890	3.95113	0.02768	5.97771
O	4	-0.49805	1.99973	6.47383	0.02449	8.49805
C	5	0.21295	1.99893	3.76624	0.02188	5.78705
C	6	-0.05357	1.99898	4.03515	0.01944	6.05357
C	7	-0.16442	1.99906	4.14762	0.01774	6.16442
C	8	-0.18812	1.99916	4.16891	0.02005	6.18812
C	9	-0.12907	1.99917	4.11046	0.01944	6.12907
C	10	-0.19627	1.99915	4.17717	0.01994	6.19627
C	11	-0.14330	1.99907	4.12617	0.01807	6.14330
C	12	0.14737	1.99909	3.83598	0.01757	5.85263
C	13	-0.45452	1.99889	4.42354	0.03209	6.45452
C	14	0.76552	1.99922	3.20874	0.02651	5.23448
O	15	-0.52601	1.99963	6.49878	0.02760	8.52601
C	16	0.74351	1.99922	3.23128	0.02599	5.25649
O	17	-0.52209	1.99963	6.49460	0.02786	8.52209
C	18	-0.69173	1.99915	4.67525	0.01734	6.69173
C	19	-0.70311	1.99916	4.68644	0.01751	6.70311
H	20	0.26870	0.00000	0.72957	0.00173	0.73130
H	21	0.27082	0.00000	0.72601	0.00317	0.72918
H	22	0.24142	0.00000	0.75712	0.00145	0.75858
H	23	0.27313	0.00000	0.72484	0.00203	0.72687
H	24	0.27548	0.00000	0.72249	0.00203	0.72452
H	25	0.29679	0.00000	0.70189	0.00132	0.70321
H	26	0.31657	0.00000	0.68161	0.00182	0.68343
H	27	0.27168	0.00000	0.72634	0.00198	0.72832
H	28	0.31823	0.00000	0.68045	0.00131	0.68177
H	29	0.29855	0.00000	0.70009	0.00136	0.70145
H	30	0.56410	0.00000	0.43319	0.00271	0.43590
H	31	0.56398	0.00000	0.43330	0.00272	0.43602
H	32	0.22761	0.00000	0.77049	0.00190	0.77239
H	33	0.22747	0.00000	0.77088	0.00165	0.77253
H	34	0.22419	0.00000	0.77438	0.00143	0.77581
H	35	0.34995	0.00000	0.64809	0.00196	0.65005
H	36	0.22715	0.00000	0.77120	0.00165	0.77285
H	37	0.22580	0.00000	0.77246	0.00173	0.77420
H	38	0.33745	0.00000	0.66051	0.00205	0.66255
* Total *		3.00000	37.98425	97.55591	0.45983	136.00000

Cation J
Energy E(B3LYP) = -923.875763548 h, G^{298} = -923.572312 h, μ=19.3 D

Cartesian coordinates, Å

N	atom	x	y	z
1	C	1.579802	2.668537	0.410123
2	C	1.232023	1.318538	0.410559
3	C	-0.245746	1.285225	0.794014
4	O	-0.613241	2.682681	0.969070
5	C	0.463802	3.401544	0.748721
6	H	2.541384	3.106060	0.232306
7	H	0.354582	4.469061	0.867325
8	C	-1.144069	0.813042	-0.318824
9	H	-0.890062	1.245040	-1.276800
10	C	-2.247870	0.025283	-0.269951
11	C	-2.790979	-0.516676	0.996264
12	O	-1.899948	-0.900171	1.818444
13	C	-2.956940	-0.242678	-1.526522
14	O	-3.948894	-1.032683	-1.414155
15	C	-4.215004	-0.584455	1.333676
16	H	-4.348510	-0.507453	2.411480
17	H	-4.794145	0.170865	0.811693
18	H	-4.587628	-1.568443	1.022067
19	C	-2.558723	0.295868	-2.836387
20	H	-3.229334	-0.051144	-3.617169
21	H	-2.568364	1.387172	-2.811761
22	H	-1.541443	-0.022401	-3.075293
23	H	-4.411218	-1.221692	-2.252542
24	H	-2.249630	-1.203566	2.680169
25	H	-0.412217	0.806134	1.751798
26	C	2.000041	0.148818	0.142539
27	C	1.442533	-1.105813	0.511061
28	C	3.285333	0.180794	-0.484763
29	C	2.090712	-2.304489	0.301490
30	C	3.916610	-1.041146	-0.697804
31	C	3.348797	-2.247920	-0.316936
32	H	0.490583	-1.133628	1.017780
33	H	4.882501	-1.047154	-1.182136
34	H	3.890830	-3.165808	-0.503117
35	C	3.979180	1.425560	-0.958531
36	H	4.841833	1.157739	-1.562461
37	H	3.329732	2.055977	-1.564960
38	H	4.344029	2.022849	-0.120716
39	C	1.491355	-3.620325	0.715813
40	H	2.152795	-4.145918	1.405199
41	H	0.528811	-3.483336	1.203469
42	H	1.346989	-4.267201	-0.150459

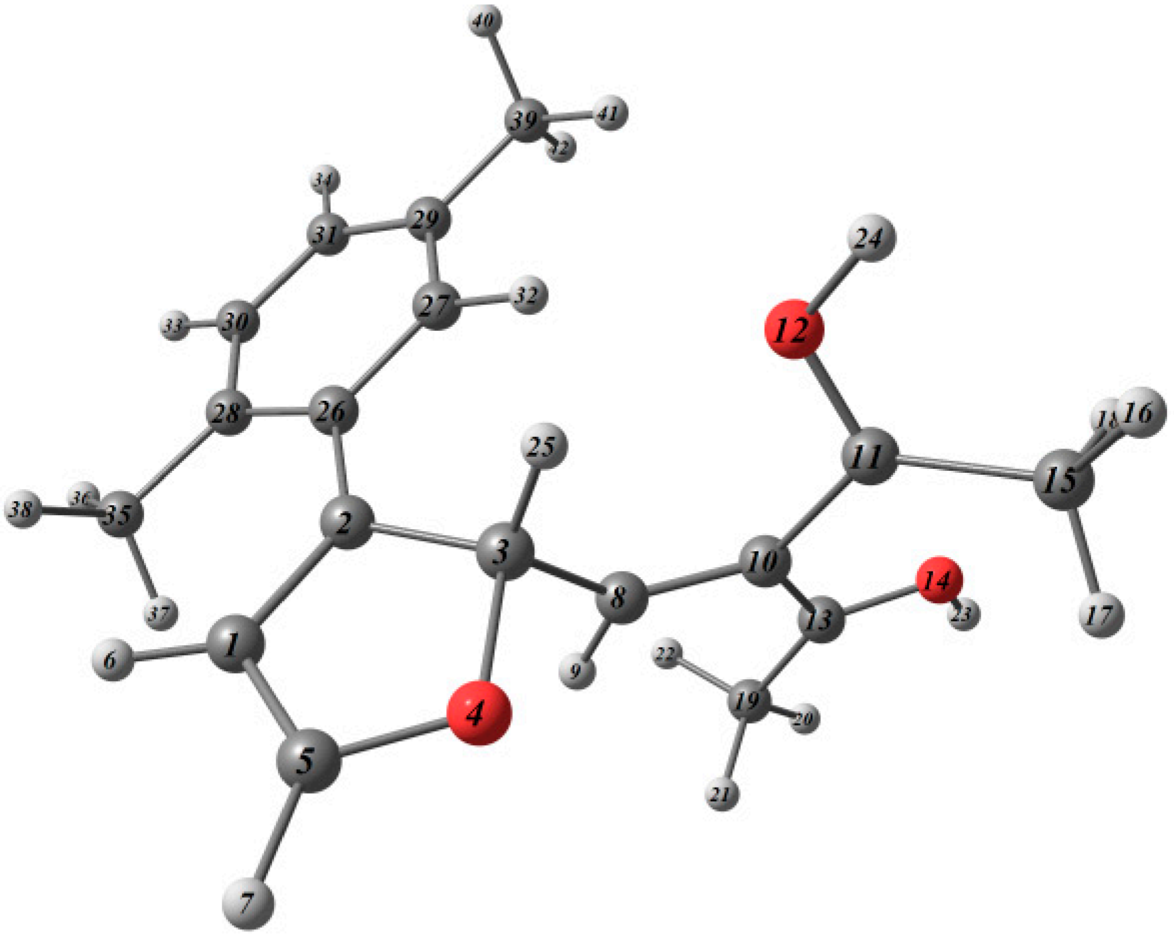
40
39
41
34
31
29
24
33
30
27
32
12
28
26
16
25
11
15
38
35
2
3
10
14
37
8
13
23
17
6
1
22
9
19
20
4
5
21
7

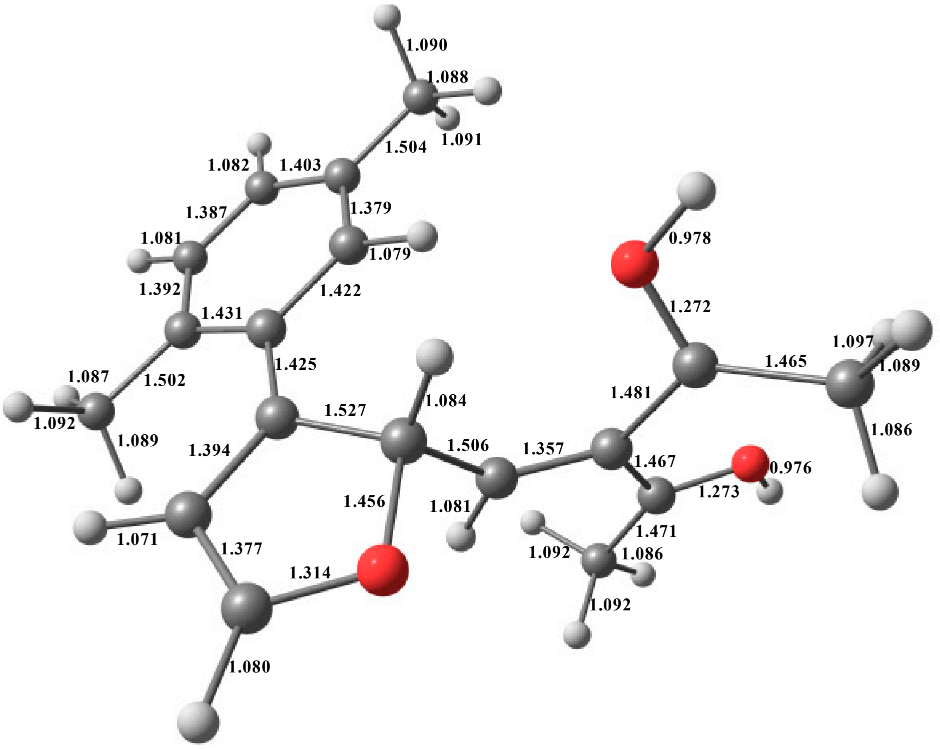
1.090
1.088
1.504
1.091
1.082
1.403
1.387
1.379
0.978
1.081
1.079
1.392
1.422
1.431
1.272
1.097
1.087
1.502
1.425
1.465
1.089
1.481
1.084
1.092
1.527
1.089
1.086
1.394
1.506
1.357
1.467
0.976
1.456
1.081
1.273
1.471
1.071
1.377
1.092
1.086
1.314
1.092
1.080

Cation K
Energy E(B3LYP) = -923.525456018 h, G^{298} = -923.234896 h, μ=14.4 D

Cartesian coordinates, Å

N	atom	x	y	z
1	C	0.547030	2.118812	-1.349853
2	C	0.424950	0.889272	-0.696349
3	C	-0.621557	1.085310	0.287403
4	O	-1.129680	2.366999	0.129899
5	C	-0.426273	2.944887	-0.833608
6	H	1.207901	2.373927	-2.156577
7	H	-0.714977	3.948073	-1.100740
8	C	-1.047184	0.301256	1.299522
9	H	-0.638469	-0.698848	1.309169
10	C	-1.999090	0.649131	2.354044
11	C	-1.871988	1.862048	3.074317
12	O	-0.794896	2.549840	2.822271
13	C	-2.963893	-0.322348	2.684330
14	O	-3.807784	-0.070401	3.644304
15	C	-2.807839	2.386844	4.098365
16	H	-2.531232	3.400687	4.377960
17	H	-3.832105	2.371421	3.734015
18	H	-2.775891	1.754401	4.986941
19	C	-3.069897	-1.621296	1.969386
20	H	-3.880862	-2.223480	2.370080
21	H	-3.245334	-1.454033	0.906785
22	H	-2.140595	-2.184437	2.067729
23	H	-4.437591	-0.794977	3.778759
24	H	-0.754747	3.372449	3.333855
25	C	1.156798	-0.337265	-0.947704
26	C	0.437977	-1.551539	-0.972142
27	C	2.552508	-0.337253	-1.189182
28	C	1.050764	-2.766077	-1.234996
29	C	3.157642	-1.571696	-1.419682
30	C	2.433665	-2.755331	-1.449076
31	H	-0.634432	-1.527212	-0.844017
32	H	4.227151	-1.604479	-1.576714
33	H	2.950378	-3.686034	-1.644263
34	C	3.408698	0.902732	-1.160927
35	H	4.447812	0.633084	-0.986302
36	H	3.370201	1.432511	-2.114703
37	H	3.106152	1.601508	-0.383236
38	C	0.265234	-4.049373	-1.301014
39	H	0.672835	-4.790271	-0.612207
40	H	-0.780689	-3.888570	-1.047988
41	H	0.308847	-4.481168	-2.302047

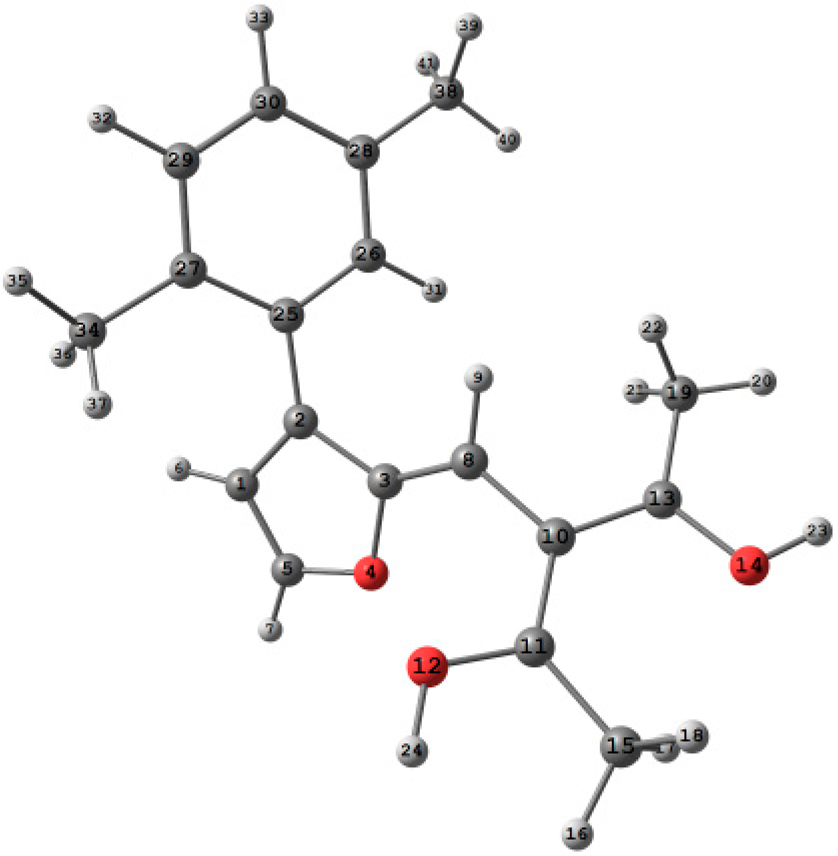
33
39
41
38
32
30
40
28
29
27
26
35
31
25
34
37
22
9
21
19
20
2
6
1
3
8
13
10
23
5
4
14
7
11
12
18
15
24
16

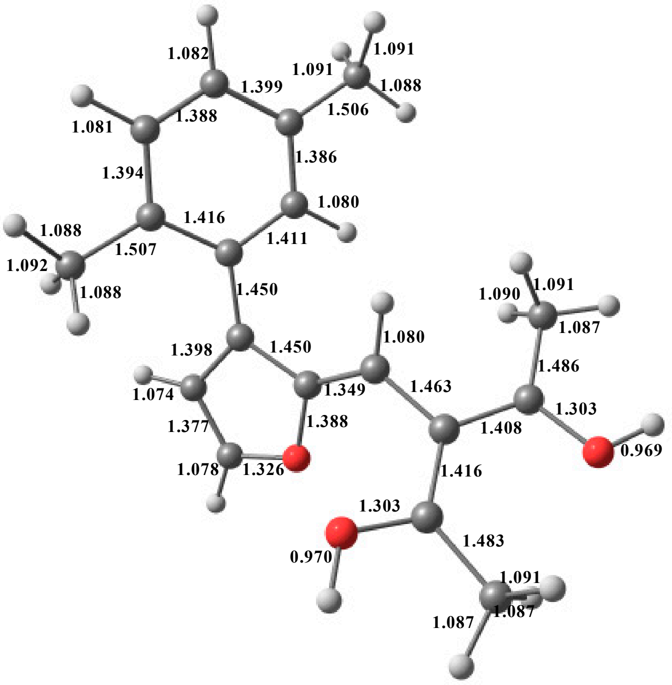
1.082
1.091
1.091
1.399
1.088
1.388
1.506
1.081
1.386
1.394
1.080
1.088
1.416
1.411
1.507
1.092
1.088
1.450
1.090
1.091
1.087
1.080
1.450
1.398
1.486
1.349
1.463
1.074
1.303
1.388
1.408
1.377
0.969
1.078
1.326
1.416
1.303
1.483
0.970
1.091
1.087
1.087

Cation K-H+
Energy E(B3LYP) = -923,875198233 h, G^{298} = -923.57282 h, μ=24.6 D

Cartesian coordinates, Å

N	atom	x	y	z
1	C	-1.299862	-2.459926	-1.117847
2	C	-1.149612	-1.176102	-0.582644
3	C	0.300087	-0.937625	-0.599304
4	O	0.910514	-2.017019	-1.224064
5	C	-0.052858	-2.890040	-1.505640
6	H	-2.209232	-3.002422	-1.289927
7	H	0.255728	-3.793143	-2.006379
8	C	1.056705	-0.004168	-0.017445
9	H	0.581880	0.793150	0.532379
10	C	2.572090	-0.047128	-0.035479
11	C	3.163293	-0.404126	1.322543
12	O	2.284812	-0.665873	2.190398
13	C	3.148319	1.190957	-0.694432
14	O	4.054653	1.789302	-0.050541
15	C	4.594162	-0.508012	1.600962
16	H	4.780924	-1.214734	2.407005
17	H	5.165144	-0.762384	0.712607
18	H	4.931036	0.481171	1.937447
19	C	2.656563	1.659732	-1.987746
20	H	3.413143	2.245833	-2.504866
21	H	2.291765	0.845223	-2.606858
22	H	1.806061	2.325777	-1.784394
23	H	4.435009	2.571143	-0.500877
24	H	2.639219	-0.964305	3.052321
25	C	-2.148261	-0.254172	-0.125740
26	C	-1.930185	1.131825	-0.336018
27	C	-3.348642	-0.690738	0.508039
28	C	-2.846336	2.090513	0.049807
29	C	-4.240557	0.294043	0.919928
30	C	-4.007369	1.644559	0.696759
31	H	-1.055480	1.447429	-0.883667
32	H	-5.143223	-0.004994	1.433838
33	H	-4.743313	2.366700	1.025210
34	C	-3.673975	-2.129329	0.803769
35	H	-4.428845	-2.184115	1.584278
36	H	-4.085822	-2.627786	-0.076244
37	H	-2.805898	-2.697555	1.132515
38	C	-2.626040	3.554238	-0.221432
39	H	-2.671542	4.129468	0.703981
40	H	-1.658522	3.730743	-0.686169
41	H	-3.398810	3.946068	-0.884068
42	H	2.895815	-0.869558	-0.692946

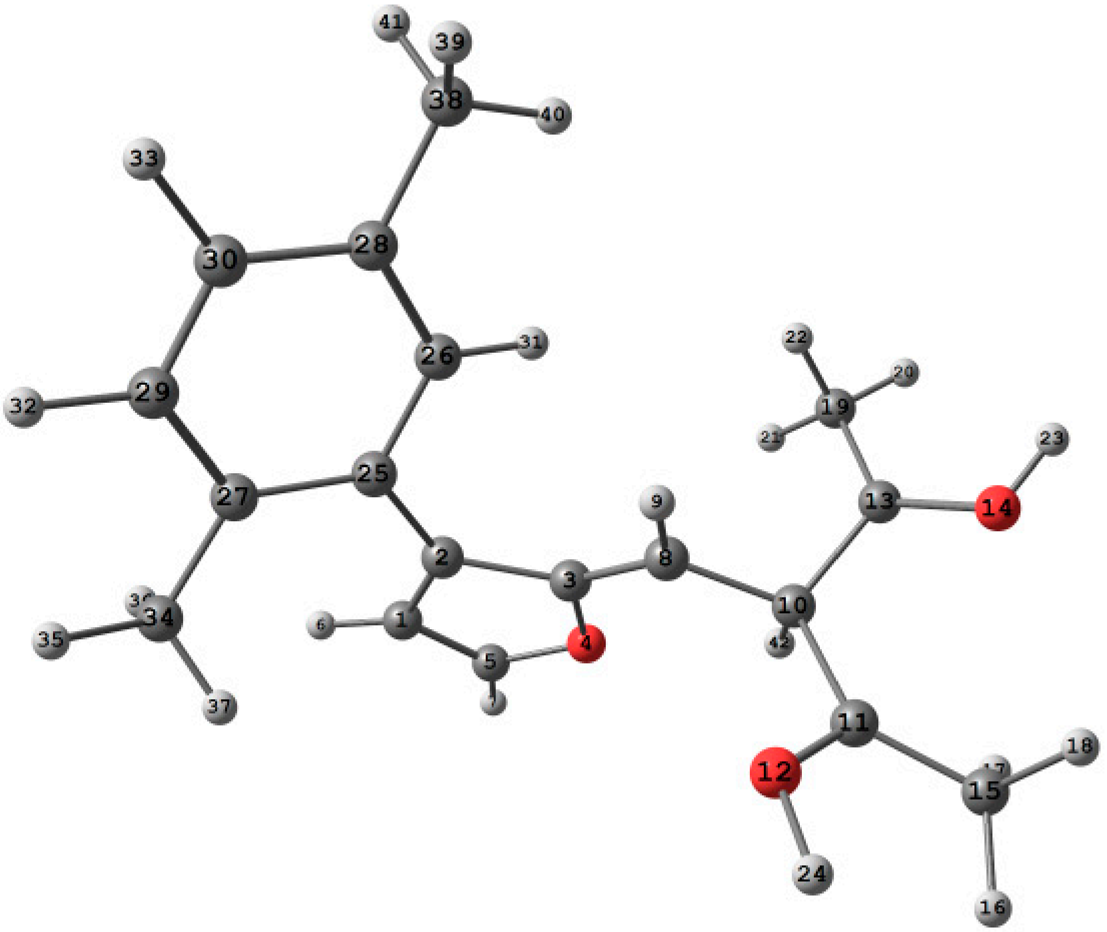
41
39
38
40
33
30
28
26
31
22
20
29
32
19
21
23
25
27
13
14
9
2
8
3
10
34
35
6
1
4
42
5
37
11
18
12
15
24
16

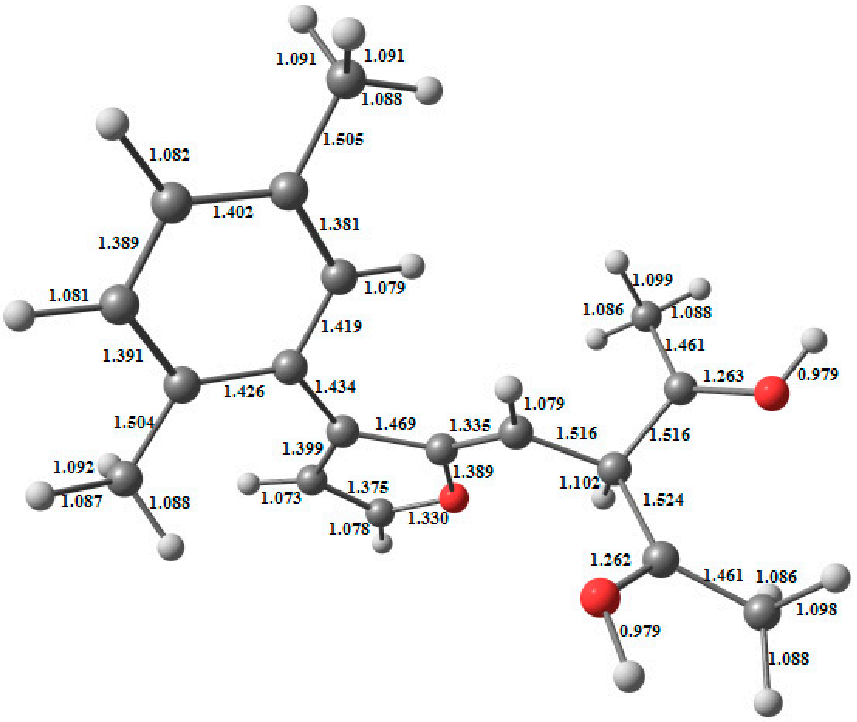
1.091
1.091
1.088
1.505
1.082
1.402
1.381
1.389
1.079
1.081
1.419
1.099
1.086
1.088
1.391
1.461
1.426
1.434
1.263
0.979
1.079
1.504
1.469
1.335
1.516
1.516
1.399
1.389
1.092
1.102
1.087
1.088
1.073
1.375
1.524
1.078
1.330
1.262
1.461
1.086
1.098
0.979
1.088

H_2O

Energy E(B3LYP) = -76.4689830023 h, G^{298} = -76.465378 h, μ=2.25 D

Cartesian coordinates, Å

N	atom	x	y	z
1	O	0.000000	0.000000	0.117683
2	H	0.000000	0.761687	-0.470732
3	H	0.000000	-0.761687	-0.470732

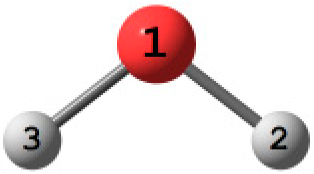

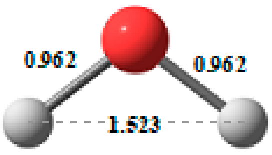

H_3O^+

Energy E(B3LYP) = -76.8576841255 h, G^{298} = -76.841757 h, μ=1.78 D

Cartesian coordinates, Å

N	atom	x	y	z
1	O	0.000000	-0.000024	-0.086324
2	H	-0.791493	-0.478470	0.230221
3	H	-0.018935	0.924355	0.230155
4	H	0.810429	-0.445693	0.230220

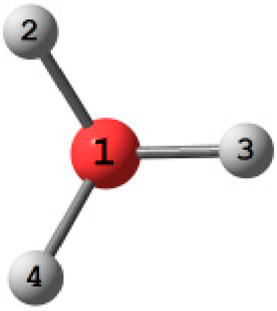

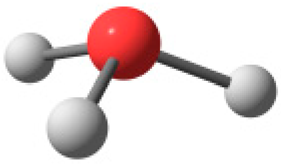
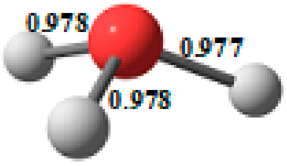

4. Study of biological activity

The antimicrobial activity of the test objects was determined at the following concentrations: 256, 128, 64, 32, 16, 8, 4, 2, and 1 μg/mL.

The following microbial strains were used as the test system: *Escherichia coli* ATCC 25922, *Staphylococcus aureus* ATCC 6538-P, *Candida albicans* ATCC 10231.

The study employed the following culture media:

- for cultivation of microorganisms (*Escherichia coli*, *Staphylococcus aureus*) — Trypticase Soy Agar (Sigma-Aldrich, USA);
- for fungi — Sabouraud Agar (NICF, Russia).

For the microplate assay, the following media were used:

- for microorganisms (*Escherichia coli*, *Staphylococcus aureus*) — Mueller-Hinton Broth (HIMEDIA, India);
- for fungi (*Candida albicans*) — RPMI-1640 (LLC NPP PanEco, Russia).

Culture media were prepared from commercially available dry media in accordance with the manufacturer's instructions.

Prior to the experiment, test microorganisms were activated by inoculation onto culture media. After obtaining microbial growth, solutions of the test objects were prepared. Stock solutions with a concentration of 1024 μg/mL were prepared by dissolving weighed portions of the test objects in dimethyl sulfoxide (DMSO) (Biolot, Russia). The study utilized a stock solution of 1024 μg/mL (containing 25% DMSO) to prepare working solutions in accordance with Table S3.

Table S3 — Scheme for preparing the working solutions of the test objects for the microplate method.

Concentration of the test object in the stock solution, μg/mL	Volume of the stock solution, mL	Volume of the culture broth, mL	The resulting concentration of the test object in the working solution, μg/mL	The final concentration of the test object in the microplate wells with the added microbial test strain, μg/mL	Final DMSO concentration in microplate wells, %
1024	1,0	1,0	512	256	6,25
512	1,0	1,0	256	128	3,13
256	0,25	0,25	128	64	1,56
256	0,5	1,5	64	32	0,78
64	0,5	0,5	32	16	0,39
64	0,5	1,5	16	8	0,20
16	0,5	0,5	8	4	0,10
16	0,5	1,5	4	2	0,05
4	0,5	0,5	2	1	0,02

To prepare the working solutions, culture broths (Mueller-Hinton Broth, RPMI-1640) were used, depending on the microbial strain employed. All working solutions of the test objects were prepared at double the required concentration, since, when adding the working solutions to the microplate wells (50 µL for bacteria and 100 µL for fungi), an equal volume (50 µL for bacteria and 100 µL for fungi) of inoculum containing the microbial strain was added to them. Four wells were used per concentration of the test object in the plate. Thus, two test objects were included in one plate.

An example of the distribution of the test object solutions in the microplate wells is presented in Table S4.

Table S4 — Distribution of the test object solutions in the microplate wells

	**1**	**2**	**3**	**4**	**5**	**6**	**7**	**8**	**9**	**10**	**11**	**12**
				Testobject №1								
**A**	C-	C+	-	256	128	64	32	16	8	4	2	1
**B**	C-	C+	-	256	128	64	32	16	8	4	2	1
**C**	C-	C+	-	256	128	64	32	16	8	4	2	1
**D**	C-	C+	-	256	128	64	32	16	8	4	2	1
				Testobject№2								
**E**	C-	C+	-	256	128	64	32	16	8	4	2	1
**F**	C-	C+	-	256	128	64	32	16	8	4	2	1
**G**	C-	C+	-	256	128	64	32	16	8	4	2	1
**H**	C-	C+	-	256	128	64	32	16	8	4	2	1

Notes:
C− — Control of the culture medium
C+ — Control of the test microorganism growth
«–» — Empty cells
256–1 — Final concentration of the test object in µg/mL

After adding the working concentrations of the test objects to the microplate wells, the microbial inocula were prepared. The inoculum was prepared by diluting a colony suspension. Several isolated colonies were picked up with a bacteriological loop and transferred to a sterile saline solution. The suspension was adjusted to achieve a turbidity equivalent to 0.5 McFarland units; turbidity was measured using a densitometer (DEN-1 McFarland Densitometer, SIA "BioSan", Latvia). A properly adjusted McFarland suspension contains approximately 1×10^8 CFU/mL for typical bacteria, and 10^6 to 5×10^6 CFU/mL for yeast fungi.The inoculum was diluted in broth to obtain a final cell concentration of 5×10^5 CFU/mL for bacteria (within the range of 2×10^5 to 8×10^5 CFU/mL). For fungi, the final cell concentration in the plate should be in the range of $0,5 \times 10^3$ to $2,5 \times 10^3$ CFU/mL.The plates were inoculated within 30 minutes after preparing the inoculum to maintain the concentration of viable cells. One row containing 100 or 200 µL of culture medium inoculated with the test microorganism was used as a growth

control for each test microorganism. A row of wells containing 100 or 200 µL of culture medium without the test object and not inoculated was used as a negative control.

To confirm the amount of microorganisms introduced into the microplate wells, ten-fold dilutions of the inoculum were prepared, followed by plating 100 µL onto Petri dishes. The Petri dishes were then incubated in an incubator at a temperature of +34 to +37 °C for (18 ± 2) hours for microorganisms, and for (22 ± 2) hours for fungi.

After adding all the agents, the plates were covered with lids and incubated in an incubator at a temperature of +34 to +37 °C for 18 hours.

The final DMSO concentrations were evaluated in separate microplates under identical conditions in the absence of the test compounds.

After incubation, the plates were examined in transmitted light for the presence of microbial growth in the wells. The minimum inhibitory concentration (MIC) of the test objects corresponded to the well with the lowest concentration of the test objects showing no signs of visible growth of the test microorganisms (clear broth).

After incubating the Petri dishes with the inoculum dose control in the incubator, the number of cells in the microplate wells was counted, taking into account the dilution with the test objects.

The number of bacterial cells in the plates was as follows: *Escherichia coli* = $3{,}2 \times 10^5$ CFU/mL, *Staphylococcus aureus* = $8{,}0 \times 10^5$ CFU/mL, and *Candida albicans* = $1{,}0 \times 10^3$ CFU/mL. Thus, the inoculum dose control confirmed the required number of cells introduced into the plate wells: for bacteria, within the range of 2×10^5 to 8×10^5 CFU/mL; for yeast-like microorganisms, within the range of $0{,}5 \times 10^3$ to $2{,}5 \times 10^3$ CFU/mL.

The results from the plates were evaluated visually in transmitted light, provided there was sufficient growth of the test microorganism (i.e., a distinct spot or definite turbidity in the wells with the growth control — «C+»), and no growth (turbidity) was observed in the non-inoculated wells (culture medium control — «C−»), in accordance with Figure S49.

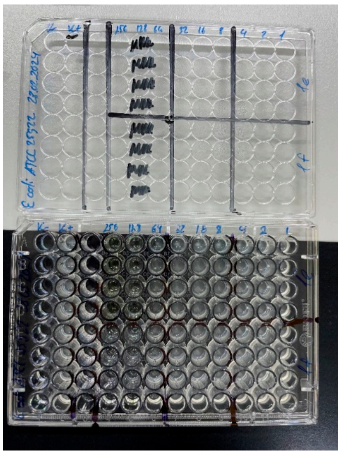

Figure S49 — Plate inoculated with *E. coli* and test objects No. 1e, No. 1f.

The testing of microplates with final DMSO concentrations ranging from 6,25% to 0,02% confirmed that the solvent had no effect on microbial growth within the wells (Figures S50, S51, S52). Microbial growth in these wells was consistent with that observed in the control wells.

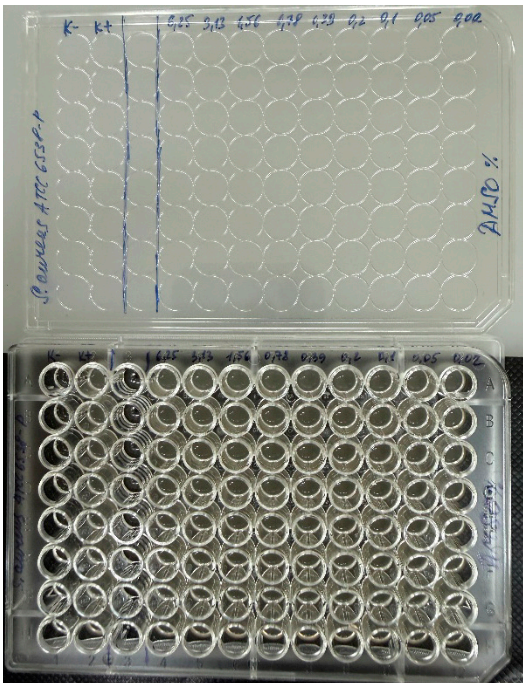

Figure S50 — Plate inoculated with *S. aureus* and DMSO

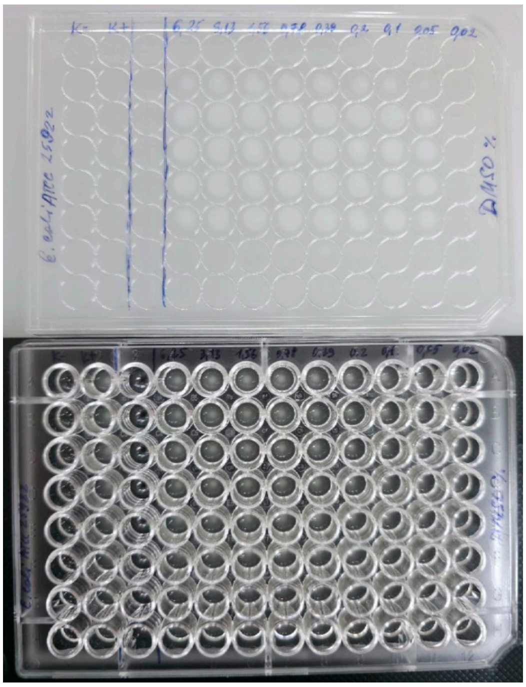

Figure S51 — Plate inoculated with *E. coli* and DMSO

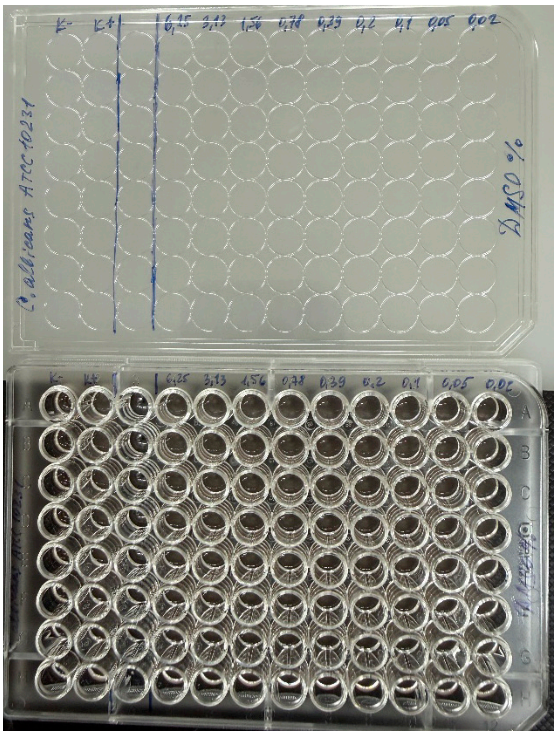

Figure S52 — Plate inoculated with *C. albicans* and DMSO
